# Extensive diversity of RNA viruses in ticks revealed by metagenomics in northeastern China

**DOI:** 10.1371/journal.pntd.0011017

**Published:** 2022-12-21

**Authors:** Ziyan Liu, Liang Li, Wenbo Xu, Yongxu Yuan, Xiaojie Liang, Li Zhang, Zhengkai Wei, Liyan Sui, Yinghua Zhao, Yanyan Cui, Qing Yin, Dajun Li, Qianxue Li, Zhijun Hou, Feng Wei, Quan Liu, Zedong Wang

**Affiliations:** 1 Department of Infectious Diseases, Center of Infectious Diseases and Pathogen Biology, Key Laboratory of Organ Regeneration and Transplantation of the Ministry of education, The First Hospital of Jilin University, State Key Laboratory of Zoonotic Diseases, Changchun, Jilin Province, People’s Republic of China; 2 Laboratory of Pathogen Microbiology and Immunology, College of Life Science, Jilin Agricultural University, Changchun, Jilin Province, People’s Republic of China; 3 Changchun Veterinary Research Institute, Chinese Academy of Agricultural Sciences, Changchun, Jilin Province, People’s Republic of China; 4 College of Food Science and Engineering, Jilin Agricultural University, Changchun, Jilin Province, People’s Republic of China; 5 School of Life Sciences and Engineering, Foshan University, Foshan, Guangdong Province, People’s Republic of China; 6 College of Food Science and Engineering, Tonghua Normal University, Tonghua, Jilin Province, People’s Republic of China; 7 College of Wildlife and Protected Area, Northeast Forestry University, Harbin, Heilongjiang Province, People’s Republic of China; Australian Red Cross Lifelood, AUSTRALIA

## Abstract

**Background:**

Ticks act as important vectors of infectious agents, and several emerging tick-borne viruses have recently been identified to be associated with human diseases in northeastern China. However, little is known about the tick virome in northeastern China.

**Methods:**

Ticks collected from April 2020 to July 2021 were pooled for metagenomic analysis to investigate the virome diversity in northeastern China.

**Results:**

In total, 22 RNA viruses were identified, including four each in the *Nairoviridae* and *Phenuiviridae* families, three each in the *Flaviviridae*, *Rhabdoviridae*, and *Solemoviridae* families, two in the *Chuviridae* family, and one each in the *Partitiviridae*, *Tombusviridae* families and an unclassified virus. Of these, eight viruses were of novel species, belonging to the *Nairoviridae* (Ji’an nairovirus and Yichun nairovirus), *Phenuiviridae* (Mudanjiang phlebovirus), *Rhabdoviridae* (Tahe rhabdovirus 1–3), *Chuviridae* (Yichun mivirus), and *Tombusviridae* (Yichun tombus-like virus) families, and five members were established human pathogens, including Alongshan virus, tick-borne encephalitis virus, Songling virus, Beiji nairovirus, and Nuomin virus. *I*. *persulcatus* ticks had significant higher number of viral species than *H*. *japonica*, *H*. *concinna*, and *D*. *silvarum* ticks. Significant differences in tick viromes were observed among Daxing’an, Xiaoxing’an and Changbai mountains.

**Conclusions:**

These findings showed an extensive diversity of RNA viruses in ticks in northeastern China, revealing potential public health threats from the emerging tick-borne viruses. Further studies are needed to explain the natural circulation and pathogenicity of these viruses.

## Author summary

Ticks are important vectors to transmit many infectious agents. In recent years, many novel tick-borne viruses associated with human illness, including Alongshan virus, Songling virus, and Beiji nairovirus, have been identified in northeastern China. It is necessary to conduct routine surveillance of tick-borne viruses in ticks, however, little is known about the tick virome in northeastern China. In this study, we used metatranscriptomics to investigate the virome diversity in four tick species collected from northeastern China. In total, we identified 22 RNA viruses belonging to eight virus families (exclude an unclassified virus). Of these, five members were established human pathogens, eight viruses were of novel species, and six viruses had close relationship with pathogenic tick-borne viruses with potential public health threats. Moreover, we found that the viromes were significantly affected by the tick species and geographical location in the study. These findings revealed an extensive diversity of RNA viruses in ticks in northeastern China, which will lay the foundation for the prevention and control of emerging tick-borne diseases.

## Introduction

Ticks are obligate haematophagous ectoparasites of wild and domestic animals as well as humans, and there are approximately 900 tick species worldwide, of which many can transmit pathogenic agents, including viruses, bacteria, and protozoa [1]. Several tick-borne viruses (TBVs) are associated with serious diseases in humans and animals, such as tick-borne encephalitis virus (TBEV), Crimean-Congo hemorrhagic fever virus (CCHFV) and Nairobi sheep disease virus (NSDV) [2]. In recent decades, the incidence and geographical distribution of tick-borne viruses have an increasing tendency, highlighting the public health importance of these arboviruses [3]. Due to the application of next generation sequencing (NGS) in recent years, many novel viruses have been identified in different tick species of different regions worldwide [4–7]. To our knowledge, the known identified TBVs include hundreds of viral members of at least 12 genera in 9 families of two orders as well as other unassigned members [8].

In China, tick-borne encephalitis virus (TBEV) in the *Flaviviridae* family and Crimean-Congo hemorrhagic fever virus (CCHFV) in the *Nairoviridae* family are the two causative agents of viral encephalitis and hemorrhagic fever in northeastern and northwestern regions, respectively, that are transmitted by different tick species [9–11]. Emerging TBVs, such as severe fever with thrombocytopenia virus (SFTSV) [12,13], Jingmen tick virus (JMTV) [14,15], Alongshan virus (ALSV) [16], Songling virus (SGLV) [17], Beiji nairovirus (BJNV) [18], Tacheng tick virus 1 and 2 [19,20], have been reported to be associated with human diseases. Nairobi sheep disease virus (NSDV), another member in the *Nairoviridae* family that can cause an acute hemorrhagic gastroenteritis in sheep and goats, has also been detected in ticks in China [21,22]. Therefore, it is necessary to conduct routine surveillance of tick-borne viruses. The tick viromes have been analyzed in several provinces, including Heilongjiang, Liaoning, Hebei, Henan, Yunnan and Guangdong in China [23–28], revealing a large number of novel RNA viruses of vertebrate and invertebrate hosts. These studies also suggest that the viromes are significantly affected by the tick species and geographical location.

The northeastern region has the richest forest resources in China, mainly concentrated in the Daxing’an mountain (DXAM), Xiaoxing’an mountain (XXAM) and Changbai mountain (CBM), resulting in abundant tick populations. In addition to TBEV, several emerging tick-borne viruses that may infect humans have been discovered in northeastern China, such as ALSV [16], SGLV [17], BJNV [18], and JMTV [15]. Here, using metagenomic analysis, we found extensive diversity of RNA viruses in ticks in northeastern China, revealing potential public threats from the emerging TBVs. Further studies are needed to explain the natural circulation and pathogenicity of these viruses.

## Methods

### Sample collection

**Fig 1:**
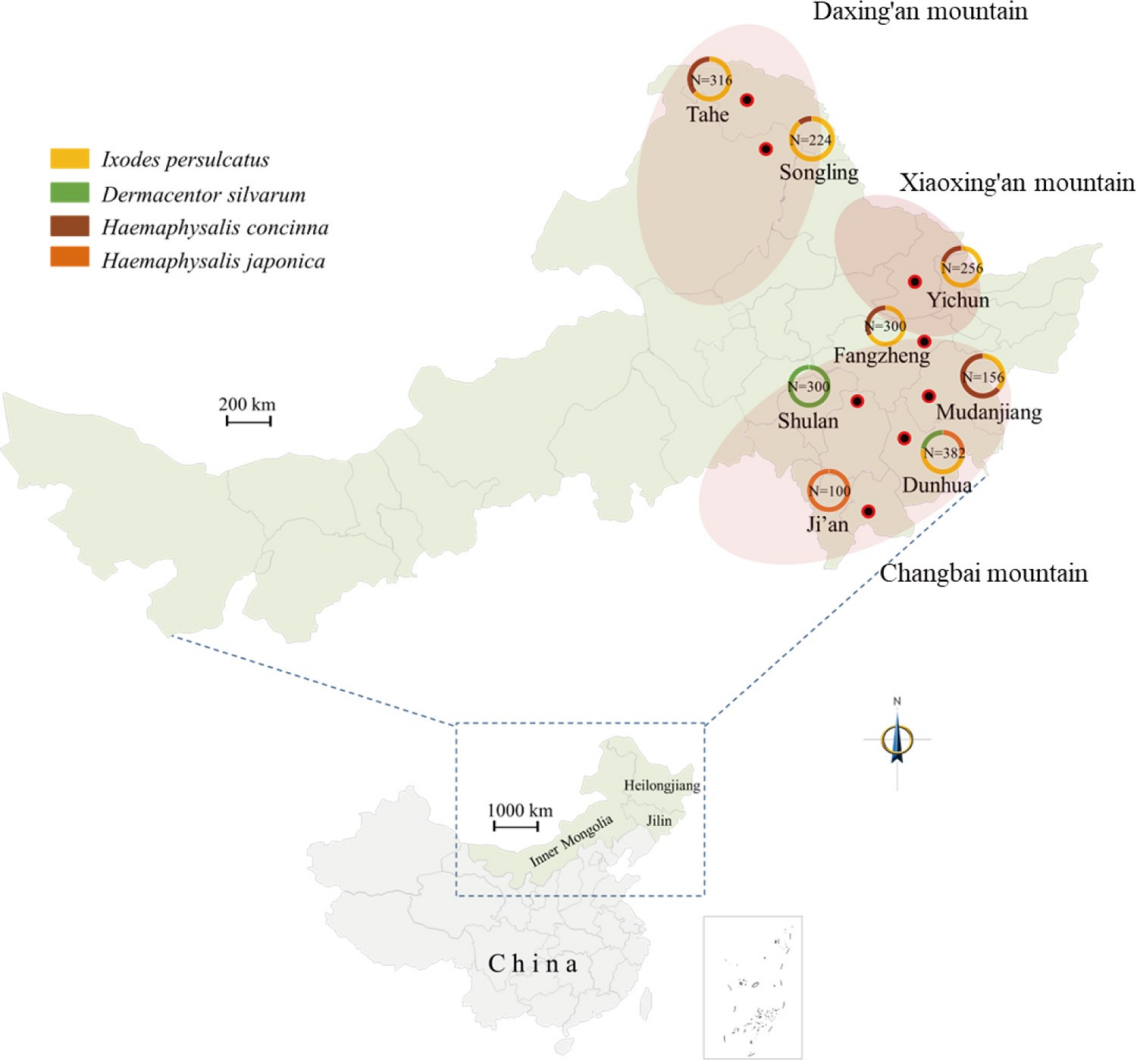
Sampling sites of ticks in northeastern China. The number of ticks collected at each site was marked, and species was shown by different colors. Yellow, *Ixodes persulcatus*; Green, *Dermacentor silvarum*; Brown, *Haemaphysalis concinna*; Orange, *Haemaphysalis japonica*. The link to the base layer of the map is http://bzdt.ch.mnr.gov.cn/index.html.

From April 2020 to July 2021, the questing ticks were collected by the flagging method in Northeastern China, and the blood-sucking ticks were only collected from cattle in Shulan (Fig 1). These ticks were identified to species as described elsewhere [29, 30]. The questing ticks were pooled to 10 ticks per tube based on the tick species and sampling sites, and *Dermacentor silvarum* ticks collected from cattle were grouped and pooled to 1, 5, or 10 ticks per tube based on the engorgement levels (fully engorged, partially engorged, or unengorged). All the ticks were stored at -80°C until use.

### RNA library construction and sequencing

After washing with 75% ethanol and RNA/DNA-free water, pooled ticks in tubes were added with 500 μL Dulbecco’s modified Eagle’s minimum essential medium (DMEM) and two stainless steel beads (3 mm diameter), and crushed using the Tissuelyser (Jingxin, Shanghai, China) at 70 Hz for 2 min. The lysates were centrifuged at 12000 rpm for 10 min at 4°C, and the supernatant was further pooled for library construction according to the collection sites and species (S1 Table). After digested with micrococcal nuclease (NEB, USA) in 37°C for 2 h, the pooled samples were used for viral RNA extraction with the TIANamp Virus RNA kit (TIANGEN, Beijing, China).

The extracted RNA was subjected to metagenomic sequencing at Tanpu Biological Technology (Shanghai, China). Briefly, the RNA from each pool was used for library preparation with the NEBNext Ultra RNA Library Prep Kit for Illumina (NEB, USA) according to the manufacturer’s instructions. After adapter ligation, 10 cycles of PCR amplification were performed for target enrichment. The libraries were pooled at equal molar ratio, denatured and diluted to optimal concentration, and sequenced with an Illumina NovaSeq 6000 System.

### Transcriptome analysis

Transcriptome analysis was conducted as described elsewhere [31]. Briefly, after trimming and removing low quality reads, the paired-raw reads were purified by removing ribosomal RNA, host contamination, and bacteria sequences using BBMap program (https://github.com/BioInfoTools/bbmap), and assembled into contigs with SPAdes v3.14.1 (https://github.com/ablab/spades) and SOAPdenovo v2.04 (https://github.com/aquaskyline/SOAPdenovo-Trans) [32,33]. After being compared with the nonredundant nucleotide (nt) and protein (nr) database downloaded from GenBank using BLAST+ v2.10.0, the assembled contigs were filtered to remove the host and bacterial sequences. The relative abundance of the identified viruses was determined by mapping the reads back to the assembled contigs using Bowtie2 v2.3.3.1.

### Viral genome confirmation and annotation

The assembled contigs were compared with NCBI nucleotide and viral refseq database using BLAST (V2.10.0+), and used as a reference for designing specific primers to confirm and analyze the sequences of terminal ends, using the nested reverse transcription-polymerase chain reaction (RT-PCR) and the rapid amplification of cDNA ends (RACE) as described elsewhere [17,18]. Potential open reading frames (ORFs) in the viral sequences were predicted using ORFfinder (https://www.ncbi.nlm.nih.gov/orffinder/).

### Virus classification

All the viruses identified in this study were classified according to the latest International Committee on Taxonomy of Viruses (ICTV) report of virus taxonomy (https://talk.ictvonline.org/ictvreports/ictv_online_report/). A novel viral species should be satisfied with one of the following conditions as described before [24], namely, (i) <80% nucleotide (nt) identity across the complete genome; or (ii) <90% amino acid (aa) identity of the RNA-dependent RNA polymerase (RdRp) domain with the known viruses. All novel viruses were named as the collection sites that the virus was first identified, followed by common viral names according to their taxonomy. All the viral strains would be marked with ‘Northeastern (NE)’ to distinguish them from the virus strains identified in other studies.

### Phylogenetic analyses

To confirm the phylogenetic relationships of the viruses discovered in this study, representative reference viral sequences were retrieved from the GenBank database (S2 Table), and aligned using ClustalW available within MEGA 7.0. Phylogenetic analyses were conducted with the aligned sequences using the maximum-likelihood method in MEGA version 7.0 with the best-fit substitution model for each alignment [34]. A bootstrapping analysis of 1000 replicates was conducted in the analysis, and the bootstrap values more than 70 were shown in the trees.

## Results

### Tick collection and identification

A total of 2,031 adult ticks, including 204 *Haemaphysalis japonica*, 393 *H*. *concinna*, 386 *Dermacentor silvarum* and 1048 *Ixodes persulcatus* ticks, were collected from NE China (Fig 1, S1 Table). The collection sites were distributed in Ji’an (n = 100), Dunhua (n = 382), and Shulan (n = 300) in Jilin Province, Fangzheng (n = 300), Mudanjiang (n = 156), Yichun (n = 253), and Tahe (n = 316) in Heilongjiang Province, and Songling (n = 224) in Inner Mongolia Autonomous Region (Fig 1, S1 Table). Of these, five were located in Changbai Mountain (CBM), two in Daxing’an Mountain (DXAM), and one in Xiaoxing’an Mountain (XXAM) (Fig 1, S1 Table).

### Identified RNA viruses

A total of 24 RNA libraries were constructed and sequenced, resulting in 245.8 GB clean data and ∼0.5 billion non-rRNA reads (S1 Table). Totally, 2,059 viral contigs were obtained by de novo assembly from ∼0.93 million viral reads that accounted for 0.2% of the total non-rRNA reads. Within each library, the viral reads ranged from 0.02% (library SL3) to 0.53% (library JA1) of the total non-rRNA reads. After being aligned by Blast and filtered by virus-host database, the viral contigs were finally annotated to 22 viruses, belonging to the 8 viral families, including *Flaviviridae*, *Rhabdoviridae*, *Nairoviridae*, *Phenuiviridae*, *Chuviridae*, *Partitiviridae*, *Tombusviridae*, and *Solemoviridae*, and one unclassified viral species (Table 1).

**Table 1:** Viruses identified in the present study.

Classification	Virus (abbreviation)	Genome (bp)	Closet relative (% nt identity)	No.^a^
*Flaviviridae*				
Jingmenvirus	Alongshan virus (ALSV)	3,066/2,813/2,809/2,721	Alongshan virus H3 (96.9, 98.3, 98.0, 98.6)	1
*Flavivirus*	Tick-borne encephalitis virus (TBEV)	11,048	Tick-borne encephalitis virus HLB-T74 (97.8)	3
Pestivirus-like	Bole tick virus 4 (BLTV-4)	16,281	Bole tick virus 4 Iasi20 (85.3)	2
*Nairoviridae*				
*Orthonariovirus*	Songling virus (SGLV)	12,030/4,525/2,132	Songling virus YC585 (95.9, 98.2, 98.5)	2
*Orthonariovirus*	Ji’an nairovirus (JANV)	11,993/4,404/1,802	Songling virus YC585 (73.7, 73.1, 71.9)	4
Norwavirus-like	Beiji nairovirus (BJNV)	14,899/3,767	Beiji nairovirus H160 (98.97, 98.6)	7
Norwavirus-like	Yichun nairovirus (YCNV)	14,813/3,326	Beiji nairovirus H1063 (83.0, 80.9)	3
*Phenuiviridae*				
*Phlebovirus*	Mukawa virus (MKWV)	6,422/3,309/1,887	Mukawa virus MKW73 (92.2, 92.2, 93.5)	6
*Phlebovirus*	Mudanjiang phlebovirus (MJPV)	6,428/3,330/1,882	Kuriyama virus CZCT80Q (77.5, 70.9, 72.5)	2
*Ixovirus*	Sara tick phlebovirus (STPV)	6,706/2,535	Sara tick phlebovirus Rus/Ix_persulcatus/Karelia/4/2018 (98.1, 98.8)	6
*Ixovirus*	Onega tick phlebovirus (OTPV)	6,689/1,935	Onega tick phlebovirus Rus/Ix_persulcatus/Karelia/3/2018 (98.3, 98.3)	6
*Rhabdoviridae*				
Alphanemrhavirus-like	Tahe rhabdovirus 1 (THRV1)	11,343	Manly virus (67.0)	8
Alphanemrhavirus-like	Tahe rhabdovirus 2 (THRV2)	11,486	Norway mononegavirus 1 NOR/H3/Skanevik/2014 (67.0)	2
Alphanemrhavirus-like	Tahe rhabdovirus 3 (THRV3)	10,375	Norway mononegavirus 1 NOR/H3/Skanevik/2014 (65.2)	1
*Chuviridae*				
*Mivirus*	Nuomin virus (NUMV)	10,900	Nuomin virus Rus/Ix_persulcatus/Karelia/5/2018 (97.5)	10
*Nigecruvirus*	Yichun mivirus (YCMV)	11,493	Blacklegged tick chuvirus-2 RTS126 (72.6)	2
*Tombusviridae*				
Tombusvirus-like	Yichun tombus-like virus (YTLV)	4,369	Upmeje virus OTU9. IU20 (74.39)	4
*Solemoviridae*				
*Sobemo-like*	Jilin luteo-like virus 2 (JLLV2)	2,731	Jilin luteo-like virus 2 JL-QG-2 (99.1)	6
*Sobemo-like*	Xinjiang tick associated virus 1 (XTAV1)	2,618	Xinjiang tick associated virus 1 14-YG (96.1)	4
*Sobemo-like*	*Ixodes scapularis* associated virus 1 (ISAV1)	2,658	Ixodes scapularis associated virus 1 ISE6 (82.5)	6
*Partitiviridae*				
Deltapartitivirus-like	Jilin partiti-like virus 1 (JPLV1)	1,717	Jilin partiti-like virus 1 JL/QG-4 (99.7)	7
*Unclassified*	*Ixodes scapularis* associated virus 3 (ISAV3)	1,523	Ixodes scapularis associated virus 3 ISE6 (84.6)	5

^a^ Number of RNA library with the indicated virus that was confirmed by RT-PCR and Sanger sequencing.

### Viral genomic organization and taxonomy

For each of the 22 RNA viruses, the whole genomes of the representative viral strains were further verified by Sanger sequencing (Table 1). In total, 150 viral sequences from the 22 identified RNA viruses were determined (S4 Table). Of them, 13 viruses were found in China for the first time, and eight viruses were proposed as novel viral species, as they were highly divergent to the previously identified viruses (nt identity < 80% or RdRp aa identity <90%), designated Tahe rhabdovirus 1–3 (THRV 1–3), Ji’an nairovirus (JANV), Yichun nairovirus (YCNV), Mudanjiang phlebovirus (MJPV), Yichun mivirus (YCMV), and Yichun tombus-like virus (YTLV), respectively (Table 1). Another eight viruses, including Alongshan virus (ALSV), Bole tick virus 4 (BLTV-4), Beiji nairovirus (BJNV), Jilin luteo-like virus 2 (JLLV2), Xinjiang tick associated virus 1 (XTAV1), *Ixodes scapularis* associated virus 1 (ISAV1), Jilin partiti-like virus 1 (JPLV1), and *Ixodes scapularis* associated virus 3 (ISAV3), showed the close relationships (nt identity > 80% and RdRp aa identity > 90%) with previously described tick-associated viruses that have not been approved by ICTV yet (Table 1). The remaining six viruses were all classified as known species, as they showed close relationships and identical genome organizations with ICTV-approved viruses, including tick-borne encephalitis virus (TBEV), Songling virus (SGLV), Mukawa virus (MKWV), Sara tick phlebovirus (STPV), Onega tick phlebovirus (OTPV), and Nuomin virus (NUMV) (Table 1).

### Virome composition and abundance

**Fig 2:**
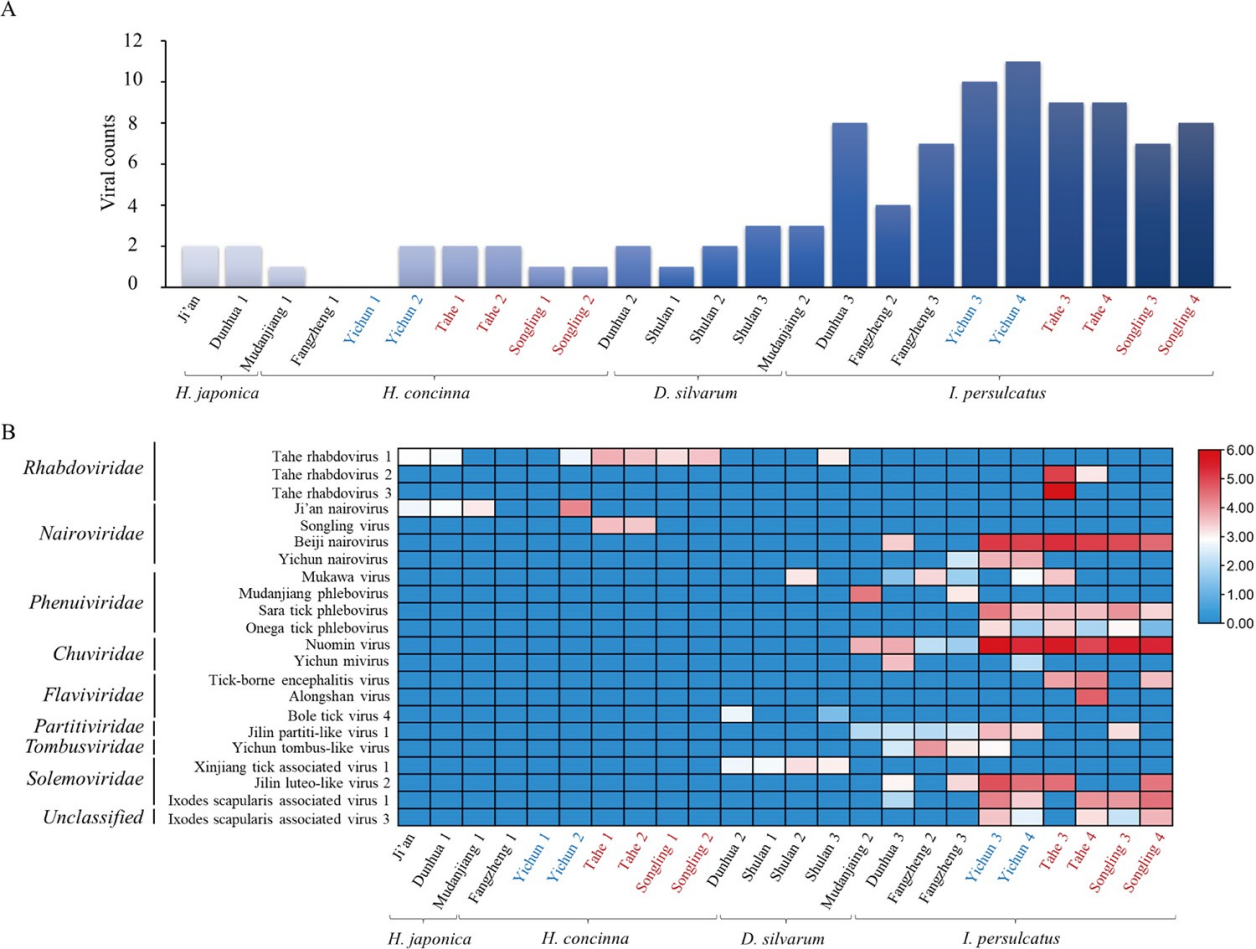
Viral presence and abundance across the libraries. (A) Viral number identified in each library. (B) Heatmap based on the normalized numbers of sequence reads for 8 viral families and one unclassified RNA virus in each library. Tick species and location information were listed at the bottom, and names of the viral families and species were indicated on the left. Log_10_ relative abundance of the viruses in each species in each location were indicated as a heat map ranging from low (blue) to high (red) based on the normalized average viral genome size and total sequencing reads in each library. The libraries from XXAM, DXAM, and CBM are marked with blue, red, and black, respectively.

Across the 24 libraries, 22 possessed 1–11 viral species, with the exception of the libraries FZ1 and YC1, which had no virus detectable (Fig 2A). The ten libraries of *I*. *persulcatus* ticks had 4–11 viral species, with 11 in the library YC4, which included BJNV, YCNV, MKWV, STPV, OTPV, NUMV, YCMV, JPLV1, JLLV2, ISAV1, and ISAV3. In contrast, *H*. *japonica*, *H*. *concinna*, and *D*. *silvarum* libraries only possessed 0–3 viral species. Of these 22 viral species, 17 species were identified in *I*. *persulcatus*, while only two, three, and four species were detected in *H*. *japonica*, *H*. *concinna*, and *D*. *silvarum* ticks, respectively (Fig 2B). Notably, most viruses were only identified in the specific tick species; however, Tahe rhabdovirus 1 was found in the *H*. *japonica*, *H*. *concinna*, and *D*. *silvarum* ticks, Ji’an nairovirus was detected in the *H*. *japonica* and *H*. *concinna* ticks, and Mukawa virus was identified in *D*. *silvarum* and *I*. *persulcatus* ticks. It should also be noted that both ALSV and THRV3 were detected in only one *I*. *persulcatus* library. Interestingly, some viral species, such as THRV2-3, SOLV, TBEV, and ALSV, were only detected in ticks from DXAM, while other viruses, including JANV, MDPV, YCNV, YCMV, BLTV4, YTLV, and XTAV1, were detected in ticks from XXAM and CBM. There were also some viruses detectable in the libraries of three regions, such as THRV1, BJNV, MKWV, NUMV, JPLV1, JLLV2, and ISAV1, showing the wide distribution of these viruses (Fig 2B). Of them, the libraries of *I*. *persulcatus* ticks had relatively higher viral reads than *H*. *japonica*, *H*. *concinna*, and *D*. *silvarum* ticks, and the libraries of XXAM (Yichun) and DXAM (Tahe and Songling) had relatively higher viral reads than that of CBM. Moreover, NUMV and BJNV had relatively higher viral reads than other viruses (Fig 2B).

### Virus diversity and evolution

#### Flaviviridae

**Fig 3:**
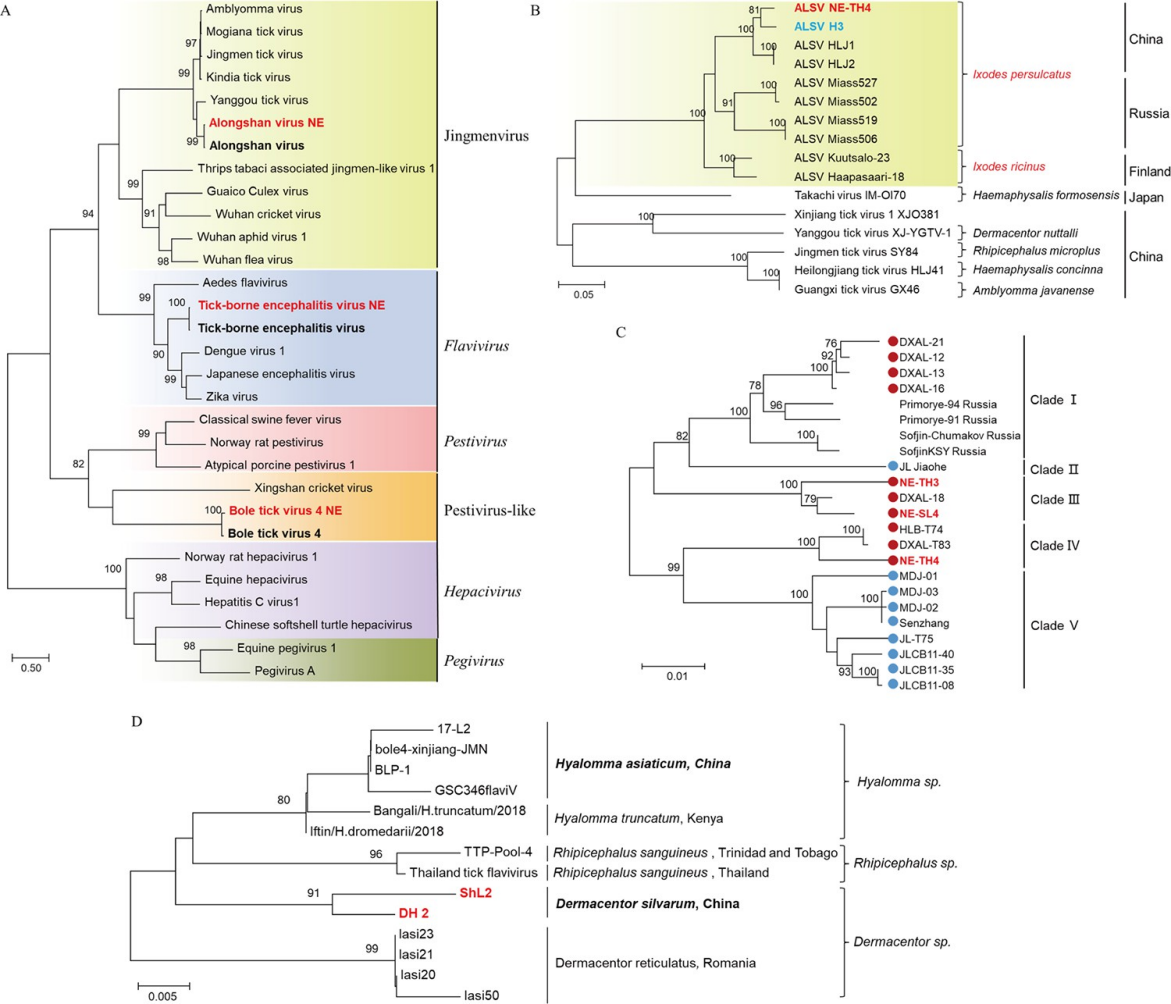
Phylogenetic analyses of flaviviruses. Phylogenetic trees were constructed based on the RdRp sequence of representative viruses in the family *Flaviviridae* (A), the segment 3 of ALSV and other representative viral strains in the Jingmenvirus group (B), the E protein of TBEV in NE China (C), and the RdRp of BLTV4 (D). All the viruses obtained in ticks here were highlighted in red. In panel A, closest referenced viruses were highlighted in bold font, and the virus genera or groups were marked with different colors of background. In panel B, ALSV isolated from humans were marked with blue. ALSV were highlighted with light green background. The host tick species of the viruses and the countries that the viruses discovered were also labeled. In panel C, TBEV isolated from CBM and XXAM were marked with blue-filled circles, while strains found in DXAM were marked with red-filled circles. In panel D, the strain names of BLTV4 were labeled. The host tick species and the countries that BLTV4 discovered were also marked. ALSV, Alongshan virus; TBEV, Tick-borne encephalitis virus; BLTV4, Bole tick virus 4. The accession numbers of the viral sequences are shown in S2 and S3 Tables.

According to the latest ICTV report of virus taxonomy, *Flavivirus*, *Hepacivirus*, *Pegivirus*, and *Pestivirus* are the approved genera in family *Flaviviridae*. While in recent years, a flavivirus-like group, the Jingmenvirus group, including a series of genetically related viruses, such as Jingmen tick virus [14] and ALSV [16], have been discovered (Fig 3A). In this study, ALSV was identified in the *I*. *persulcatus* library (TH4) from Tahe in DXAM, and clustered together with ALSV strain H3 isolated from tick-bitten patients in NE China (Fig 3B) [16], but different from the isolates detected in *I*. *persulcatus* in Russia and *I*. *ricinus* in Finland (Fig 3B), with nt identities of 97.0–98.5% (S4 and S5 Tables) [35,36].

Three libraries (TH3, TH4, and SL4) of *I*. *persulcatus* ticks from Tahe and Songling in DXAM were identified TBEV-positive, which formed a different clade from the TBEV strains in XXAM and CBM, with nt identities of 93.5–99.9% (Fig 3C, S6 Table).

Bole tick virus 4 (BLTV4)-NE strains were phylogenetically grouped into the pestivirus-like group, with nt identities of 77.5–83% and RdRp aa identities of 94.6–96.9% to other BLTV4 isolates (Fig 3A, S7 Table). BLTV4 was first identified in *Hyalomma asiaticum* ticks in Xinjiang Uygur Autonomous Region, China [37], and also discovered in Trinidad and Tobago [38], Kenya [39], Romania [40], and Thailand [41]. Phylogenetic analysis showed that BLTV4-NE was different from other strains identified in different tick species or regions (Fig 3D). In this study, the virus was only detected in two libraries of the *D*. *silvarum* ticks from CBM.

#### Nairoviridae

**Fig 4:**
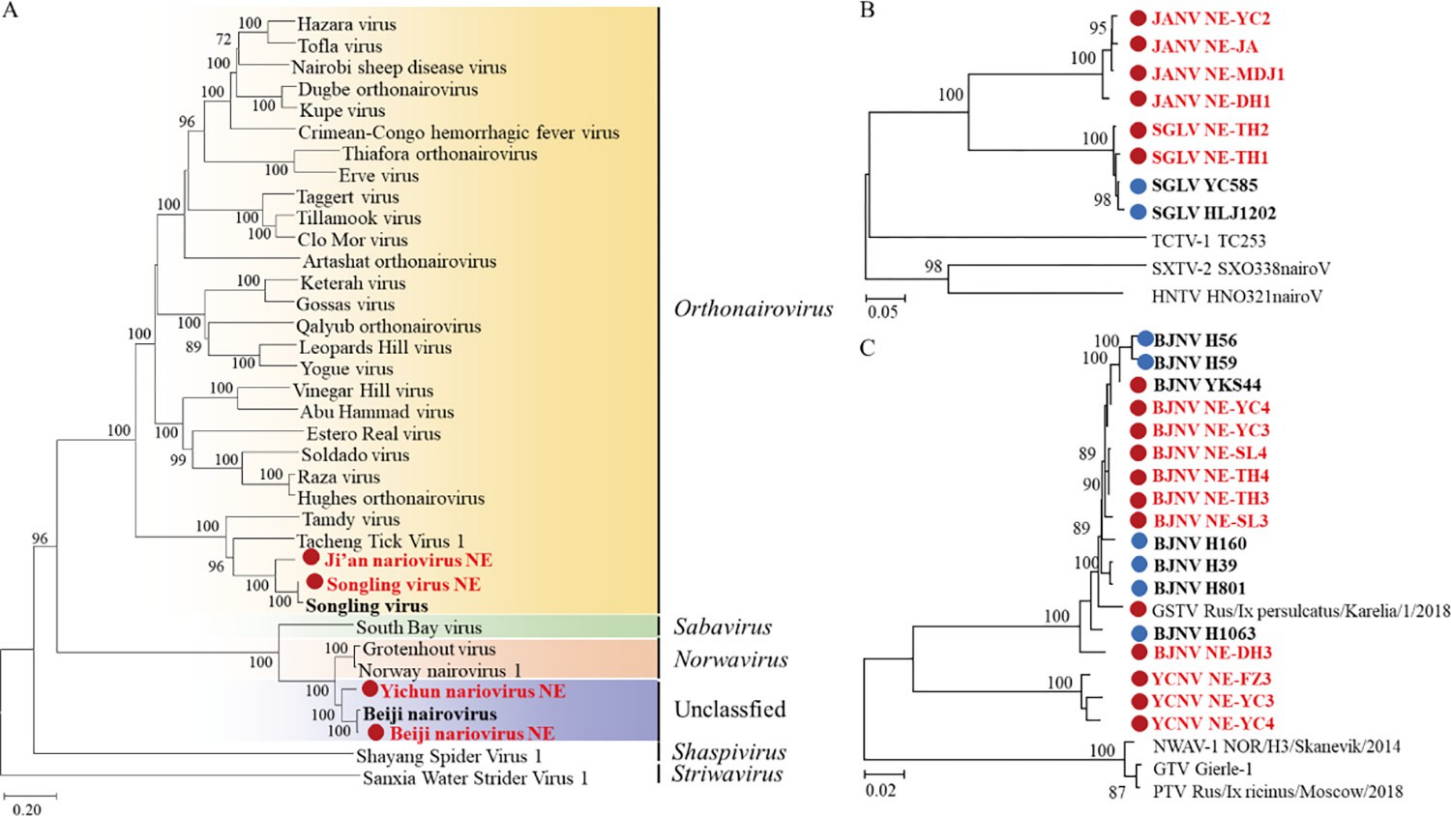
Phylogenetic analyses of nairoviruses. Phylogenetic trees were constructed based on the RdRp protein sequences of representative viruses in the *Nairoviridae* family (A), the S segment of JANV and SGLV (B), and the S segment of BJNV and YCNV (C). All the viruses obtained in ticks here were highlighted in red, and the closest referenced viruses were also highlighted in bold font. In panel B and C, viral strains identified in this study were marked with red-filled circles, while strains found in humans were marked with blue-filled circles. JANV, Ji’an nariovirus; SGLV, Songling virus; TCTV1, Tacheng tick virus 1; SXTV2, Shanxi tick virus 2; HNTV, Henan tick virus; YCNV, Yichun nariovirus; BJNV, Beiji nariovirus; GKTV, Gakugsa tick virus; PTV, Pustyn virus; NWNV1, Norway nairovirus 1; GTV, Grotenhout virus. The accession numbers of the viral sequences are shown in S2 and S3 Tables.

In the phylogenetic tree of the family *Nairoviridae*, SGLV-NE, together with SGLV, formed a separate clade from other viral members in the genus *Orthonariovirus*, including Ji’an nariovirus, Tacheng tick virus 1, and Tamdy virus (Fig 4A). SGLV-NE was identified in two libraries TH1 and TH2 of the *H*. *concinna* ticks from Tahe in DXAM, and clustered together with SGLV strains isolated from tick-bitten patients (Fig 4B), with nt identities of 92.4–99.2% (S8 and S9 Tables) [17].

JANV, genetically related to SGLV with nt identities of 70.7–73.5%, was a novel identified nairovirus (S8 and S9 Tables). Four libraries were detected JANV-positive, including DH1 and JA libraries of *H*. *japonica* ticks in CBM, and MDJ1 and YC2 of *H*. *concinna* ticks in XXAM (Fig 4B).

BJNV and YCNV, belonging to an unclassified Norwavirus-like group, showed close relationships to Norway nairovirus 1 and Grotenhout virus (Fig 4A), with nt identities of 75.4–79.6% (Segment L and S, S10 and S11 Tables). BJNV was identified in seven *I*. *persulcatus* tick libraries in all the three regions, suggesting the wide distribution of the virus in NE China. All the BJNV NE strains were clustered together with BJNV strains identified in tick-bitten patients and *I*. *persulcatus* tick in NE China and GSTV detected in Russia (Fig 4C), with nt identities of 96.2–100% (Segment L and S, S10 and S11 Tables).

YCNV was only detected in three libraries (FZ3, YC3, and YC4) of *I*. *persulcatus* ticks from Fangzheng and Yichun in CBM and XXAM. Although YCNV showed nt identities of 82.3–83.9 (>80) (Segment L and S, S10 and S11 Tables) with BJNV, the virus formed a separate clade, with aa (RdRp) identities of 87.7–88.5% (Fig 4C, S10 and S11 Tables), indicating that YCNV was a novel viral species that may be different from BJNV.

#### Phenuiviridae

**Fig 5:**
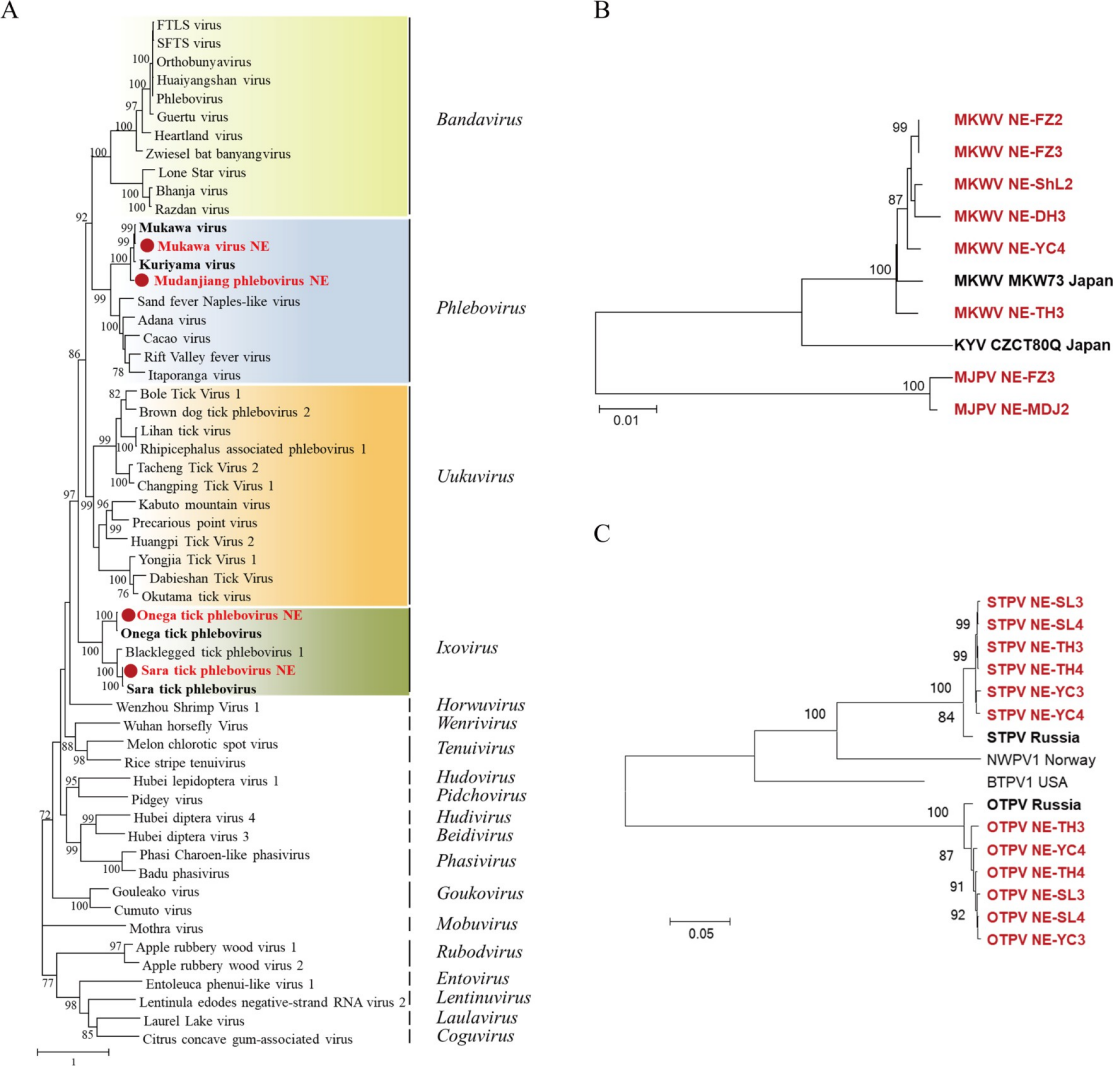
Phylogenetic analyses of phenuiviruses. Phylogenetic trees constructed based on the RdRp sequences of representative viruses in the family *Phenuiviridae* (A), the S segment of MKWV and MJPV (B), and the S segment of STPV and OTPV (C). All the viruses obtained in ticks here were highlighted in red, and the closest referenced viruses were highlighted in bold font. Abbreviations: MKWV, Mukawa virus; KYV, Kuriyama virus; MJPV, Mudanjiang phlebovirus; BTPV1, Blacklegged tick phlebovirus-1; NWPV1, Norway phlebovirus 1; STPV, Sara tick phlebovirus; OTPV, Onega tick phlebovirus. The accession numbers of the viral sequences are shown in S2 and S3 Tables.

MKWV and MJPV, together with STPV and OTPV identified here, fell within the genera *Phlebovirus* and *Ixovirus* of the family *Phenuiviridae*, respectively (Fig 5A). MKWV NE strains were clustered together with the strain MKW73 identified in *I*. *persulcatus* ticks in Japan, with nt identities of 92.4–100% for L segment, 90.7–100% for M segment, and 93.5–99.9% for S segment (Fig 5B, S12 and S13 Tables). Of the six MKWV NE strains, five were detected in the *I*. *persulcatus* ticks from Shuangzi, Dunhua, Yichun, and Tahe, while one was identified in the *D*. *silvarum* ticks collected from cattle in Shulan.

MJPV was only identified in two libraries in the *I*. *persulcatus* ticks from Fangzheng and Mudanjiang in CBM, which was distantly related to Kuriyama virus and Mukawa virus strains, with low nt identities of 75.8–77.4% for L segment, 67.6–68.7% for M segment, and 69.8–70.8% for S segment (Fig 5B, S12 and S13 Tables) [42].

STPV and OTPV NE strains found in this study were clustered with STPV and OTPV strains discovered in *I*. *persulcatus* ticks from Karelia in Russia, with nt identities of 98.0–99.2% and 98.6–99.1%, respectively (Fig 5C, S14 and S15 Tables). The two viruses formed separate clades from each other, with nt identities of 56.4–57.3%, and were identified in paired in *I*. *persulcatus* ticks in six libraries from three collection sites, including Yichun in XXAM, and Tahe and Songling in DXAM (Fig 5C, S14 and S15 Tables).

#### Rhabdoviridae

**Fig 6:**
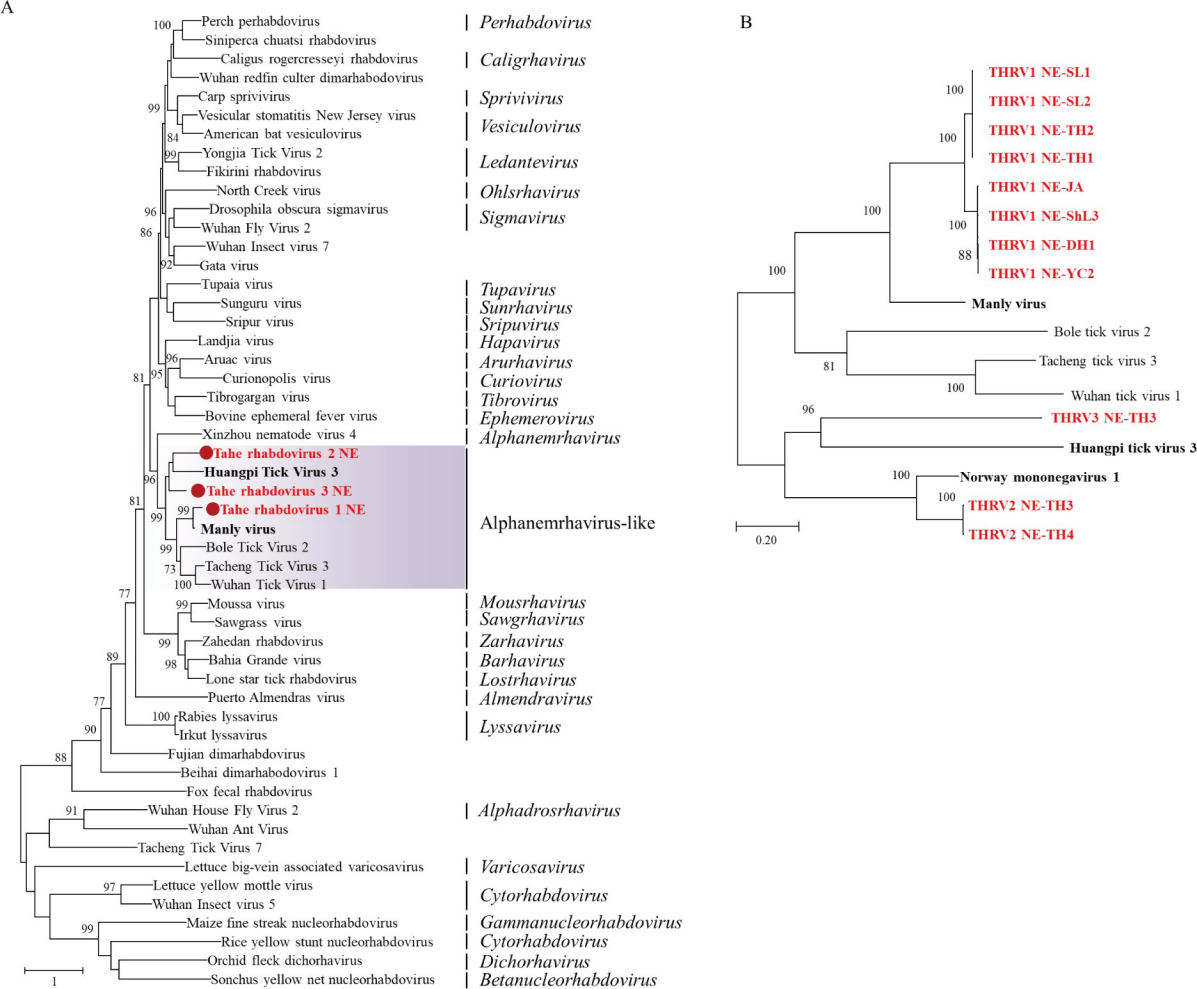
Phylogenetic analyses of rhabdoviruses. Phylogenetic trees constructed based on the RdRp sequences of representative viruses in the family *Rhabdoviridae* (A), the RdRp sequences of novel identified Tahe rhabdoviruses (B). All the viruses obtained in ticks here were highlighted in red, and the closest referenced viruses were also highlighted in bold font. Abbreviations: THRV1, Tahe rhabdovirus 1; THRV2, Tahe rhabdovirus 2; THRV3, Tahe rhabdovirus 3. The accession numbers of the viral sequences are shown in S2 and S3 Tables.

There were three novel rhabdoviruses identified in the study, namely, Tahe rhabdovirus 1 (THRV1), Tahe rhabdovirus 2 (THRV2), and Tahe rhabdovirus 3 (THRV3); they, together with Bole tick virus 2, Tacheng tick virus 3, Huangpi tick virus 3, and Wuhan tick virus 1, were genetically grouped into an unclassified Alphanemrhavirus-like group in the family *Rhabdoviridae* (Fig 6A). A total of eight libraries of *H*. *japonica*, *H*. *concinna*, and D. *silvarum* ticks were detected THRV1, which formed close relationship with Manly virus, with nt identities of 30.4–30.6% and RdRp aa identities of 70.2–70.7%, respectively (Fig 6B, S16 Table). However, THRV1 were divided into two different clades: one clade was identified from libraries of Ji’an, Dunhua, Shulan, and Yichun in CBM and XXAM, while the other clade found in libraries from Tahe and Songling in DXAM, with nt identities of 81.4% and RdRp aa identities of 93.6–93.7% (Fig 6B, S16 Table). Compared with THRV1, THRV2 and THRV3 were only identified in the *I*. *persulcatus* tick from Tahe in DXAM, with nt identities of 44.9–49.3% with THRV1 and 45.9–46.1% with each other (Fig 6B, S16 Table).

#### Chuviridae

**Fig 7:**
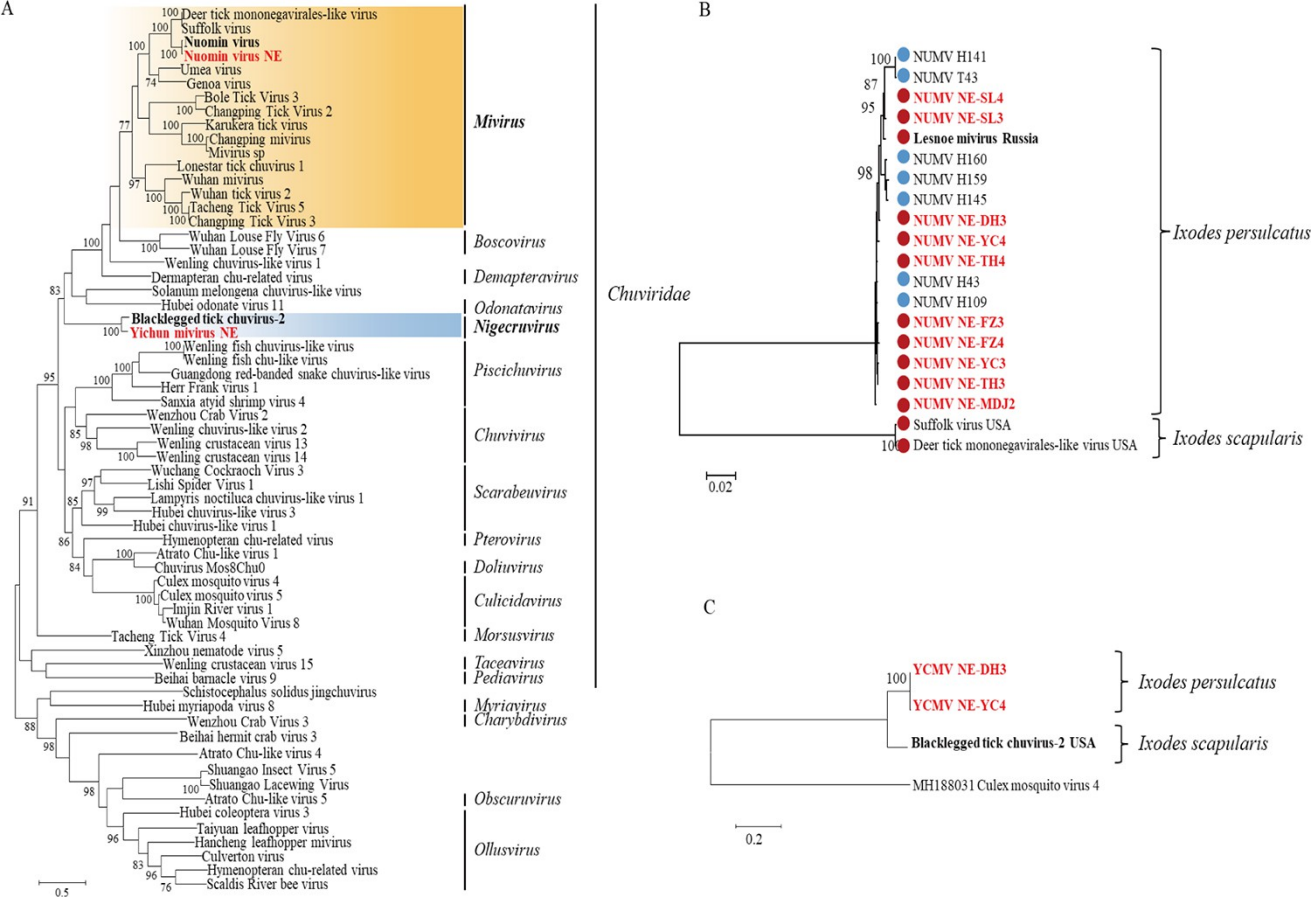
Phylogenetic analyses of chuviruses. Phylogenetic trees were constructed based on the RdRp sequences of representative viruses in the family *Chuviridae* (A), the RdRp sequences of NUMV (B) and JLCV (C). All the viruses obtained in ticks here were highlighted in red, and the closest referenced viruses were also highlighted in bold font. In panel B, NUMV viral strains discovered from ticks were marked with red-filled circles, while strains found in tick-bite patients were marked with blue-filled circles. Abbreviations: NUMV, Nuomin virus; YCMV, Yichun mivirus. The accession numbers of the viral sequences are shown in S2 and S3 Tables.

Nuomin virus (NUMV) and Yichun mivirus (YCMV) identified here fell within the *Mivirus* and *Nigecruvirus* genera in the *Chuviridae* family, respectively (Fig 7A). NUMV NE strains were detected in all the ten *I*. *persulcatus* libraries in the three regions in NE China, while no libraries from other tick species were identified NUMV positive, indicating the wide distribution and specificity of host tick species of the virus. The viral strains of NUMV found in this study were clustered with other NUMV strains discovered in humans from NE China and Lesnoe mivirus isolated from the *I*. *persulcatus* ticks in Russia, showing nucleotide identities of 96.6–99.5% with each other (S17 Table).

YCMV was a novel virus identified in this study, and clustered together with Blacklegged tick chuvirus 2 found in *I*. *scapularis* ticks from USA, with nt identities of 68.5%. YCMV was only detected in the *I*. *persulcatus* ticks from Dunhua and Yichun in CBM and XXAM (Fig 7C), and shared high nt identity of 99.2%.

#### Plant-related viruses

**Fig 8:**
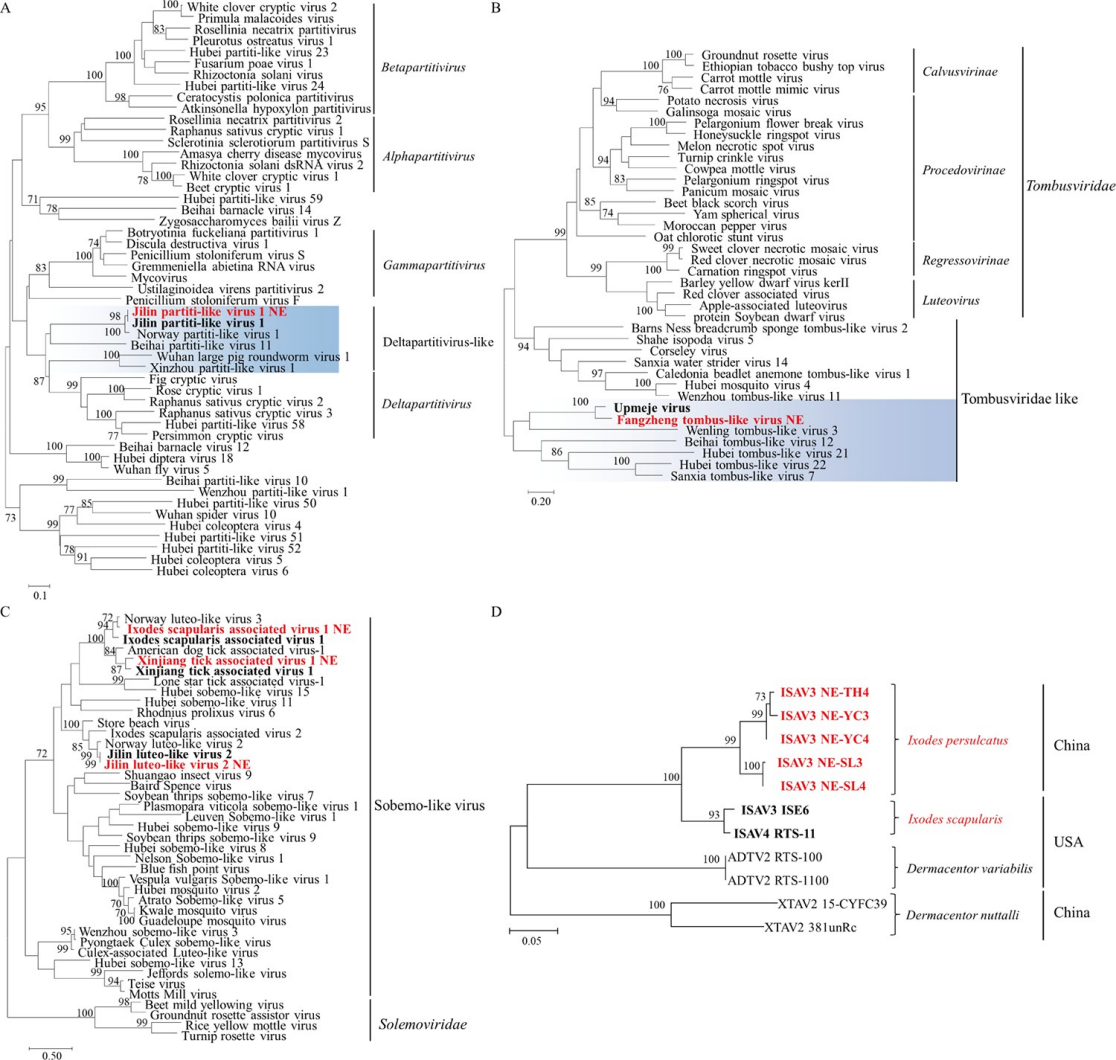
Phylogenetic analyses of plant-related viruses. Phylogenetic trees constructed based on the RdRp protein sequences of representative viruses in the family *Partitiviridae* (A), *Tombusviridae* (B), *Solemoviridae* (C), and ISAV3 (D). All the viruses obtained in ticks here were highlighted in red, and the closest referenced viruses were also highlighted in bold font. Abbreviations: JPLV1, Jilin partiti-like virus 1; NPLV1, Norway partiti-like virus 1; FTLV, Fangzheng tombus-like virus; ISAV3, Ixodes scapularis associated virus 3; ISAV4, Ixodes scapularis associated virus 4; XTAV2, Xinjiang tick associated virus 2; ADTV, American dog tick associated virus 2. The accession numbers of the viral sequences are shown in S2 and S3 Tables.

Several viral species closely related to plant viruses were found in this study. The Jilin partiti-like virus 1 (JPLV1) identified here fell within an unclassified clade, provisionally designated as Deltapartitivirus-like group related to the *Partitiviridae* family (Fig 8A), and was closely related to Norway partiti-like virus 1 strains in Norway with high identities of nt 91.3–91.9% (S18 Table). The newly discovered Fangzheng tombus-like virus (FTLV) fell within the Tombusviridae-like group associated with family *Tombusviridae* (Fig 8B), sharing a close relationship (identity: nt 73.4–73.6% and aa 80.6–81.3%) to Upmeje virus strain OTU9.IU20 that has been identified in *Ixodes uriae* in Sweden (S19 Table) [43].

Three *Solemoviridae*-like viral species, including *Ixodes scapularis* associated virus 1 (ISAV1), Xinjiang tick associated virus 1 (XTAV1), and Jilin luteo-like virus 2 (JLLV2), were found in this study, and fell within the Sobemo-like virus group associated with the family *Solemoviridae* (Fig 8C). In the aa RdRp phylogenetic tree, ISAV1-NE was clustered with Norway luteo-like virus 3 identified in *Ixodes Ricinus* ticks in Norway and *Ixodes scapularis* associated virus 1 identified in *I*. *scapularis* ticks in USA, with nt identities of 81.5–85.7% (S20 Table). The XTAV1-NE strains were clustered together with XTAV1 strains in Xinjiang with nt identities of 95.9–96.2% (S20 Table). The JLLV2 strains were detected in the *I*. *persulcatus* ticks and showed a close relationship with Norway luteo-like virus 2 identified in *Ixodes ricinus* ticks in Norway, with nt identities of 87.7–88.2% (S21 Table).

*Ixodes scapularis* associated virus 3 (ISAV3) is still unclassified to date, but the strains identified in this study showed close relationship with ISAV3 and ISAV4 discovered in *Ixodes scapularis* ticks from USA with nt identities of 86.7–88.2% (Fig 8D, S22 Table) [44, 45].

## Discussion

We show the metagenomic description of the RNA viruses in ticks in NE China. Analysis of the transcriptomes of *I*. *persulcatus*, *D*. *silvarum*, *H*. *concinna*, and *H*. *japonica* ticks demonstrated that these ticks harbored a wide diversity of RNA viruses, belonging to at least 8 families of *Flaviviridae*, *Nairoviridae*, *Phenuiviridae*, *Rhabdoviridae*, *Chuviridae*, *Partitiviridae*, *Tombusviridae*, and *Solemoviridae*. Abundant viruses of the families *Flaviviridae*, *Nairoviridae*, *Phenuiviridae*, and *Chuviridae* detected in the ticks were consistent with those of previous studies, suggesting that viruses of these families have a wide geographical distribution [4,37–39,45–51].

Previous metagenomic analysis of ticks in Heilongjiang has revealed viral contigs annotated to South Bay virus (SBV), blacklegged tick phlebovirus (BTPV), deer tick mononegavirales-like virus (DTMV), and Jingmen tick virus (JMTV) [23]. Due to lack of whole genome sequence of these viruses, it is difficult to accurately define their classification. In this study, we obtained the complete genome of 16 viral species, including 3 flaviviruses (ALSV, TBEV and BLTV-4), 4 nairoviruses (SGLV, JANV, BJNV, and YCNV), 4 phenuiviruses (MV, KV, STPV, and OPTV), 3 rhabdoviruses (THRV-1, 2, and 3), and 2 chuviruses (NUMV and YCMV), showing an extensive diversity of RNA viruses in ticks in northeastern China.

There were significantly differences in tick viromes among CBM, XXAM, and DXAM. In this study, ALSV, TBEV, and THRV2-3 were only detected in DXAL, while MJPV, YCNV, YCMV, and FTLV were identified in CBM and XXAL. Nairovirus SGLV was detected in DXAM, while its closely related virus JANV was detected in CBM and XXAM; Rhabdovirus THRV1 tended to evolve into two genotypes: one was distributed in DXAM, the other distributed in CBM and XXAM. Additionally, the genetic differences of TBEV also supports the occurrence of geographical barriers of tick-borne viruses in northeastern China.

Segmented flaviviruses have recently been reported as emerging tick-borne viruses, with a wide distribution in Asia, Africa, Europe, Central America, and South America [46,47]. Of them, Jingmen tick virus (JMTV) and ALSV are associated with the febrile illness in tick-bitten patients in northeastern China. JMTV has been found in various vertebrates, including cattle, sheep, rodents, and non-human primate, showing that the virus cocirculates between ticks and mammals [46]; JMTV and ALSV have also been detected in mosquitoes [14,16]; however, the transmission modes of these viruses remain to be investigated. In this study, only *I*. *persulcatus* tick was tested ALSV-positive in DXAM, where ALSV patients have been recently found [16], suggesting potential public health risk of ALSV infection and limited distribution of the emerging tick-borne virus in northeastern China. The pathogenicity of segmented flaviviruses in humans and animals needs to be further verified through animal infection models.

Interestingly, co-feeding transmission might affect the viromes in ticks collected from animals. In this study, two viral species, THRV1 and MKWV, identified in *D*. *silvarum* collected from cattle (Shulan), were mainly found in *H*. *japonica* and *H*. *concinna*, and *I*. *persulcatus* ticks, respectively. However, the two viruses were not detected in the questing *D*. *silvarum* ticks in Dunhua adjacent to Shulan. As cattle can act as hosts of ticks, such as *D*. *silvarum*, *H*. *japonica*, *H*. *concinna*, and *I*. *persulcatus* ticks, and co-feeding transmission may be an efficiency way of viral transmission in ticks [52–54]. *D*. *silvarum* collected from cattle here may contract the viruses from *Haemaphysalis sp*. or *I*. *persulcatus* ticks by co-feeding transmission, but not the actual vector of these viruses. Thus, it is suggested to investigate tick virome diversity using questing ticks instead of ticks collected from animals. Moreover, further studies should be focus on the vector competence of ticks for the transmission of the identified viruses, which may further confirm the roles of different tick species for virus transmission.

The Bunyavirales order includes important human pathogens, such as Crimean-Congo hemorrhagic fever virus in the *Nairoviridae* family, and SFTSV and Valley fever virus in the *Phenuiviridae* family, whose genome are negative single-stranded RNA of small (S), medium (M), and large (L) segments, encoding structural nucleoprotein (NP), glycoprotein precursor (GPC), and RNA-dependent RNA polymerase (L) proteins, respectively. Recently, several bi-segmented viruses without the M gene have been found in nairoviruses (such as Gakugsa tick virus and Norway nairovirus 1) and phleboviruses (such as Tacheng tick virus 2) [48–51]. We also identified four viruses, including YCNV and BJNV within the *Nairoviridae* family, and STPV together with OTPV in the *Phenuiviridae* family. However, we did not find the M gene encoding the glycoproteins. The possible reason may be the substantially genetical diversity of these viruses from the reference viruses [18,19].

We also found several viral species closely related to plant viruses, including JPLV1 in the *Partitiviridae* family, FTLV in the *Tombusviridae* family, and ISAV1, XTAV1, and JLLV2 in the *Solemoviridae* family. Compared with viruses in the *Flaviviridae*, *Nairoviridae* and *Phenuiviridae* families, plant-related viruses identified here may have low pathogenicity to humans or mammals.

There are some limitations to the present study. Although some tick-borne viruses associated with diseases in humans or mammals, including TBEV, ALSV, SGLV, BJNV, and NUMV, were detected in this study, some other pathogenic viruses, such as severe fever with thrombocytopenia syndrome virus (SFTSV) [55,56], lymphocytic choriomeningitis virus (LCMV) [57], Nairobi sheep disease virus (NSDV) [21], and Jingmen tick virus (JMTV) [15] identified in previous studies, were not detected here, indicating that larger tick samples and wider sampling sites are necessary to characterize the tick-borne viruses in NE China. Some novel viruses had close relationship with TBVs of public health significance. For example, JANV and YCNV were genetically related to SGLV and BJNV, respectively, which have been shown to be associated with febrile diseases, indicating the potentially pathogenic to humans and animals of these novel viruses. As in the *Phenuiviridae* family, however, although MKWV does not detected in tick-bitten patients, it can grow in a human-derived cells and mice, and its nonstructural proteins can suppress the anti-innate immune responses [42], suggesting the potential public health significance of MKWV and its closely related virus (MJPV), and the necessity of epidemiology studies on tick-bitten patients, livestock, and even wild animals.

## Conclusions

These findings showed an extensive diversity of RNA viruses in ticks in northeastern China, revealing potential public health threats from the emerging tick-borne viruses. Further studies are needed to explain the natural circulation and pathogenicity of these viruses.

## Supporting information

S1 Table Summary of sample collection and library, and RNA sequencing. (DOCX)

S2 Table Reference viruses used in the present study. (DOCX)

S3 Table Nucleotide sequences of the identified viruses in the present study. (DOCX)

S4 Table Nucleotide sequence similarity of S1 (upper right) and S2 (lower left) segments of ALSV. (DOCX)

S5 Table Nucleotide sequence similarity of S3 (upper right) and S4 (lower left) segments of ALSV. (DOCX)

S6 Table Nucleotide sequence similarity of TBEVs. (DOCX)

S7 Table Nucleotide sequence similarity of the complete genome (upper right) and amino acid sequence similarity of RdRp (lower left) of BLTV4. (DOCX)

S8 Table Nucleotide sequence similarity of L segment (upper right) and amino acid sequence similarity of RdRp (lower left) of JANV and SGLV. (DOCX)

S9 Table Nucleotide sequence similarity of M (upper right) and S (lower left) segments of JANV and SGLV. (DOCX)

S10 Table Nucleotide sequence similarity of the L segment (upper right) and amino acid sequence similarity of RdRp (lower left) of YCNV and BJNV. (DOCX)

S11 Table Nucleotide sequence similarity of the S segment (upper right) of YCNV and BJNV. (DOCX)

S12 Table Nucleotide sequence similarity of the L segment (upper right) and amino acid sequence similarity of the RdRp (lower left) of MKV and MJPV. (DOCX)

S13 Table Nucleotide sequence similarity of the S (upper right) and M segments (lower left) of MKV and MJPV. (DOCX)

S14 Table Nucleotide sequence similarity of the L segment (upper right) and amino acid sequence similarity of RdRp (lower left) of STPV and OTPV. (DOCX)

S15 Table Nucleotide sequence similarity of the S segment (upper right) of STPV and OTPV. (DOCX)

S16 Table Nucleotide sequence similarity of the complete genome (upper right) and amino acid sequence similarity of RdRp (lower left) of THRV1, THRV2, THRV3. (DOCX)

S17 Table Nucleotide sequence similarity of the complete genome (upper right) and amino acid sequence similarity of RdRp (lower left) of NUMV. (DOCX)

S18 Table Nucleotide sequence similarity of RdRp genome (upper right) and amino acid sequence similarity of RdRp (lower left) of JPLV1. (DOCX)

S19 Table Nucleotide sequence similarity of the complete CDS (upper right) and amino acid sequence similarity of RdRp (lower left) of FLTV. (DOCX)

S20 Table Nucleotide sequence identities of complete cds (upper right) and amino acid sequence identities of RdRp (lower left) of ISAV1 and XTAV1. (DOCX)

S21 Table Nucleotide sequence identities of complete cds (upper right) and amino acid sequence identities of RdRp (lower left) of JLLV2. (DOCX)

S22 Table Nucleotide sequence identities of complete genome (upper right) of ISAV3. (DOCX)

## References

[1] Mansfield KL, Jizhou L, Phipps LP, Johnson N. Emerging tick-borne viruses in the twenty-first century. *Front Cell Infect Microbiol*. 2017;7:298. doi: 10.3389/fcimb.2017.00298 28744449 PMC5504652

[2] Madison-Antenucci S, Kramer LD, Gebhardt LL, Kauffman E. Emerging tick-borne diseases. *Clin Microbiol Rev*. 2020;33(2). doi: 10.1128/CMR.00083-18 31896541 PMC6941843

[3] Medlock JM, Hansford KM, Bormane A, Derdakova M, Estrada-Pena A, George JC, et al. Driving forces for changes in geographical distribution of *Ixodes ricinus* ticks in Europe. *Parasit Vectors*. 2013;6:1. doi: 10.1186/1756-3305-6-1 23281838 PMC3549795

[4] Harvey E, Rose K, Eden JS, Lo N, Abeyasuriya T, Shi M, et al. Extensive diversity of RNA viruses in Australian ticks. *J Virol*. 2019;93(3). doi: 10.1128/JVI.01358-18 30404810 PMC6340049

[5] Damian D, Maghembe R, Damas M, Wensman JJ, Berg M. Application of viral metagenomics for study of emerging and reemerging tick-borne viruses. *Vector Borne Zoonotic Dis*. 2020;20(8):557–65. Epub 2020/04/09. doi: 10.1089/vbz.2019.2579 . 32267808

[6] Brinkmann A, Dincer E, Polat C, Hekimoglu O, Hacioglu S, Foldes K, et al. A metagenomic survey identifies Tamdy orthonairovirus as well as divergent phlebo-, rhabdo-, chu- and flavi-like viruses in Anatolia, Turkey. *Ticks Tick Borne Dis*. 2018;9(5):1173–83. doi: 10.1016/j.ttbdis.2018.04.017 . 29728337

[7] Souza WM, Fumagalli MJ, Torres Carrasco AO, Romeiro MF, Modha S, Seki MC, et al. Viral diversity of Rhipicephalus microplus parasitizing cattle in southern Brazil. *Sci Rep*. 2018;8(1):16315. doi: 10.1038/s41598-018-34630-1 30397237 PMC6218518

[8] Shi J, Hu Z, Deng F, Shen S. Tick-borne viruses. *Virol Sin*. 2018;33(1):21–43. doi: 10.1007/s12250-018-0019-0 29536246 PMC5866268

[9] Li X, Ji H, Wang D, Che L, Zhang L, Li L, et al. Molecular detection and phylogenetic analysis of tick-borne encephalitis virus in ticks in northeastern China. *J Med Virol*. 2022;94(2):507–13. doi: 10.1002/jmv.27303 . 34453752

[10] Xia H, Li P, Yang J, Pan L, Zhao J, Wang Z, et al. Epidemiological survey of Crimean-Congo hemorrhagic fever virus in Yunnan, China, 2008. *Int J Infect Dis*. 2011;15(7):e459–63. doi: 10.1016/j.ijid.2011.03.013 . 21546303

[11] Moming A, Yue X, Shen S, Chang C, Wang C, Luo T, et al. Prevalence and phylogenetic analysis of Crimean-Congo hemorrhagic fever virus in ticks from different ecosystems in Xinjiang, China. *Virol Sin*. 2018;33(1):67–73. doi: 10.1007/s12250-018-0016-3 29524182 PMC6178079

[12] Liu Q, He B, Huang SY, Wei F, Zhu XQ. Severe fever with thrombocytopenia syndrome, an emerging tick-borne zoonosis. *Lancet Infect Dis*. 2014;14(8):763–72. doi: 10.1016/S1473-3099(14)70718-2 . 24837566

[13] Li JC, Zhao J, Li H, Fang LQ, Liu W. Epidemiology, clinical characteristics, and treatment of severe fever with thrombocytopenia syndrome. *Infect Med*. 2022;1(1):40–9. doi: 10.1016/j.imj.2021.10.001 PMC10699716 38074982

[14] Qin XC, Shi M, Tian JH, Lin XD, Gao DY, He JR, et al. A tick-borne segmented RNA virus contains genome segments derived from unsegmented viral ancestors. *Proc Natl Acad Sci U S A*. 2014;111(18):6744–9. doi: 10.1073/pnas.1324194111 24753611 PMC4020047

[15] Jia N, Liu HB, Ni XB, Bell-Sakyi L, Zheng YC, Song JL, et al. Emergence of human infection with Jingmen tick virus in China: A retrospective study. *EBioMedicine*. 2019;43:317–24. doi: 10.1016/j.ebiom.2019.04.004 31003930 PMC6557783

[16] Wang ZD, Wang B, Wei F, Han SZ, Zhang L, Yang ZT, et al. A new segmented virus associated with human febrile illness in China. N Engl J Med. 2019;380(22):2116–25. doi: 10.1056/NEJMoa1805068 . 31141633

[17] Ma J, Lv XL, Zhang X, Han SZ, Wang ZD, Li L, et al. Identification of a new orthonairovirus associated with human febrile illness in China. Nat Med. 2021;27(3):434–9. doi: 10.1038/s41591-020-01228-y 33603240

[18] Wang YC, Wei Z, Lv X, Han S, Wang Z, Fan C, et al. A new nairo-like virus associated with human febrile illness in *China. Emerg Microbes Infect*. 2021;10(1):1200–8. doi: 10.1080/22221751.2021.1936197 34044749 PMC8212832

[19] Dong Z, Yang M, Wang Z, Zhao S, Xie S, Yang Y, et al. Human Tacheng tick virus 2 infection, China, 2019. *Emerg Infect Dis*. 2021;27(2):594–8. doi: 10.3201/eid2702.191486 33496245 PMC7853585

[20] Liu X, Zhang X, Wang Z, Dong Z, Xie S, Jiang M, et al. A tentative Tamdy orthonairovirus related to febrile illness in northwestern China. *Clin Infect Dis*. 2020;70(10):2155–60. doi: 10.1093/cid/ciz602 31260510

[21] Gong S, He B, Wang Z, Shang L, Wei F, Liu Q, et al. Nairobi sheep disease virus RNA in ixodid ticks, China, 2013. *Emerg Infect Dis*. 2015;21(4):718–20. doi: 10.3201/eid2104.141602 25811222 PMC4378503

[22] Yang L, Zhao Z, Hou G, Zhang C, Liu J, Xu L, et al. Genomes and seroprevalence of severe fever with thrombocytopenia syndrome virus and Nairobi sheep disease virus in *Haemaphysalis longicornis* ticks and goats in Hubei, China. *Virology*. 2019;529:234–45. doi: 10.1016/j.virol.2019.01.026 30738361 PMC7127444

[23] Meng F, Ding M, Tan Z, Zhao Z, Xu L, Wu J, et al. Virome analysis of tick-borne viruses in Heilongjiang Province, China. *Ticks Tick Borne Dis*. 2019;10(2):412–20. doi: 10.1016/j.ttbdis.2018.12.002 . 30583876

[24] Xu L, Guo M, Hu B, Zhou H, Yang W, Hui L, et al. Tick virome diversity in Hubei Province, China, and the influence of host ecology. *Virus Evol*. 2021;7(2):veab089. doi: 10.1093/ve/veab089 34804590 PMC8599308

[25] Yang Z, Zhang J, Yang S, Wang X, Shen Q, Sun G, et al. Virome analysis of ticks in a forest region of Liaoning, China: characterization of a novel hepe-like virus sequence. *Virol J*. 2021;18(1):163. doi: 10.1186/s12985-021-01632-x 34372876 PMC8351423

[26] Shi J, Shen S, Wu H, Zhang Y, Deng F. Metagenomic profiling of viruses associated with *Rhipicephalus microplus* ticks in Yunnan Province, China. *Virol Sin*. 2021;36(4):623–35. doi: 10.1007/s12250-020-00319-x 33400089 PMC8379324

[27] Zhao T, Gong H, Shen X, Zhang W, Shan T, Yu X, et al. Comparison of viromes in ticks from different domestic animals in China. *Virol Sin*. 2020;35(4):398–406. doi: 10.1007/s12250-020-00197-3 32157603 PMC7462941

[28] Guo L, Ma J, Lin J, Chen M, Liu W, Zha J, et al. Virome of *Rhipicephalus* ticks by metagenomic analysis in Guangdong, southern China. *Front Microbiol*. 2022;13:966735. doi: 10.3389/fmicb.2022.966735 36033874 PMC9403862

[29] Shao JW, Zhang XL, Li WJ, Huang HL, Yan J. Distribution and molecular characterization of rickettsiae in ticks in Harbin area of Northeastern China. *PLoS Negl Trop Dis*. 2020;14(6):e0008342. doi: 10.1371/journal.pntd.0008342 32497120 PMC7272007

[30] Deng G. Economic insect fauna of China. *Science Press*. 1978;15:1–174.

[31] Wu Q, Li JZ, Wang W, Zhou JZ, Wang DD, Fan BC, et al. Next-generation sequencing reveals four novel viruses associated with calf diarrhea. *Viruses*. 2021;13(10):1907. doi: 10.3390/v13101907 . 34696337 PMC8537473

[32] Prjibelski A, Antipov D, Meleshko D, Lapidus A, Korobeynikov A. Using SPAdes de novo assembler. *Curr Protoc Bioinformatics*. 2020;70(1):e102. doi: 10.1002/cpbi.102 32559359

[33] Xie Y, Wu G, Tang J, Luo R, Patterson J, Liu S, et al. SOAPdenovo-Trans: de novo transcriptome assembly with short RNA-Seq reads. *Bioinformatics*. 2014;30(12):1660–6. doi: 10.1093/bioinformatics/btu077 24532719

[34] Kumar S, Stecher G, Tamura K. MEGA7: Molecular evolutionary genetics analysis version 7.0 for bigger datasets. *Mol Biol Evol*. 2016;33(7):1870–4. doi: 10.1093/molbev/msw054 27004904 PMC8210823

[35] Kuivanen S, Levanov L, Kareinen L, Sironen T, Jaaskelainen AJ, Plyusnin I, et al. Detection of novel tick-borne pathogen, Alongshan virus, in *Ixodes ricinus* ticks, south-eastern Finland, 2019. *Euro Surveill*. 2019;24(27). doi: 10.2807/1560-7917.ES.2019.24.27.1900394 31290392 PMC6628756

[36] Kholodilov IS, Litov AG, Klimentov AS, Belova OA, Polienko AE, Nikitin NA, et al. Isolation and characterisation of Alongshan virus in Russia. *Viruses*. 2020;12(4). doi: 10.3390/v12040362 32224888 PMC7232203

[37] Shi M, Lin XD, Vasilakis N, Tian JH, Li CX, Chen LJ, et al. Divergent viruses discovered in arthropods and vertebrates revise the evolutionary history of the *Flaviviridae* and related viruses. *J Virol*. 2016;90(2):659–69. doi: 10.1128/JVI.02036-15 26491167 PMC4702705

[38] Sameroff S, Tokarz R, Charles RA, Jain K, Oleynik A, Che X, et al. Viral diversity of tick species parasitizing cattle and dogs in Trinidad and Tobago. *Sci Rep*. 2019;9(1):10421. doi: 10.1038/s41598-019-46914-1 31320705 PMC6639388

[39] Zhang Y, Hu B, Agwanda B, Fang Y, Wang J, Kuria S, et al. Viromes and surveys of RNA viruses in camel-derived ticks revealing transmission patterns of novel tick-borne viral pathogens in Kenya. *Emerg Microbes Infect*. 2021;10(1):1975–87. doi: 10.1080/22221751.2021.1986428 34570681 PMC8525980

[40] Bratuleanu BE, Temmam S, Chretien D, Regnault B, Perot P, Bouchier C, et al. The virome of *Rhipicephalus*, *Dermacentor* and *Haemaphysalis* ticks from Eastern Romania includes novel viruses with potential relevance for public health. *Transbound Emerg Dis*. 2021. doi: 10.1111/tbed.14105 33840161

[41] Temmam S, Chretien D, Bigot T, Dufour E, Petres S, Desquesnes M, et al. Monitoring silent spillovers before emergence: A pilot study at the tick/human interface in Thailand. *Front Microbiol*. 2019;10:2315. doi: 10.3389/fmicb.2019.02315 31681195 PMC6812269

[42] Matsuno K, Kajihara M, Nakao R, Nao N, Mori-Kajihara A, Muramatsu M, et al. The unique phylogenetic position of a novel tick-borne phlebovirus ensures an ixodid origin of the genus phlebovirus. *mSphere*. 2018;3(3). doi: 10.1128/mSphere.00239-18 29898985 PMC6001614

[43] Pettersson JH, Ellstrom P, Ling J, Nilsson I, Bergstrom S, Gonzalez-Acuna D, et al. Circumpolar diversification of the *Ixodes uriae* tick virome. *PLoS Pathog*. 2020;16(8):e1008759. doi: 10.1371/journal.ppat.1008759 32745135 PMC7425989

[44] Nakao R, Matsuno K, Qiu Y, Maruyama J, Eguchi N, Nao N, et al. Putative RNA viral sequences detected in an *Ixodes scapularis*-derived cell line. *Ticks Tick Borne Dis*. 2017;8(1):103–11. doi: 10.1016/j.ttbdis.2016.10.005 27769656

[45] Tokarz R, Sameroff S, Tagliafierro T, Jain K, Williams SH, Cucura DM, et al. Identification of novel viruses in *Amblyomma americanum*, *Dermacentor variabilis*, and *Ixodes scapularis* ticks. *mSphere*. 2018;3(2). doi: 10.1128/mSphere.00614-17 29564401 PMC5853492

[46] Guo JJ, Lin XD, Chen YM, Hao ZY, Wang ZX, Yu ZM, et al. Diversity and circulation of Jingmen tick virus in ticks and mammals. *Virus Evol*. 2020;6(2):veaa051. doi: 10.1093/ve/veaa051 33976906 PMC8097133

[47] Temmam S, Bigot T, Chretien D, Gondard M, Perot P, Pommelet V, et al. Insights into the host range, genetic diversity, and geographical distribution of Jingmenviruses. *mSphere*. 2019;4(6). doi: 10.1128/mSphere.00645-19 31694898 PMC6835211

[48] Klimentov AS, Belova OA, Kholodilov IS, Butenko AM, Bespyatova LA, Bugmyrin SV, et al. Phlebovirus sequences detected in ticks collected in Russia: Novel phleboviruses, distinguishing criteria and high tick specificity. *Infect Genet Evol*. 2020;85:104524. doi: 10.1016/j.meegid.2020.104524 32891876

[49] Pettersson JH, Shi M, Bohlin J, Eldholm V, Brynildsrud OB, Paulsen KM, et al. Characterizing the virome of *Ixodes ricinus* ticks from northern Europe. *Sci Rep*. 2017;7(1):10870. doi: 10.1038/s41598-017-11439-y 28883464 PMC5589870

[50] Sakamoto JM, Ng TFF, Suzuki Y, Tsujimoto H, Deng X, Delwart E, et al. Bunyaviruses are common in male and female *Ixodes scapularis* ticks in central Pennsylvania. *PeerJ*. 2016;4:e2324. doi: 10.7717/peerj.2324 27602290 PMC4991847

[51] Li CX, Shi M, Tian JH, Lin XD, Kang YJ, Chen LJ, et al. Unprecedented genomic diversity of RNA viruses in arthropods reveals the ancestry of negative-sense RNA viruses. *Elife*. 2015;4. doi: 10.7554/eLife.05378 25633976 PMC4384744

[52] Kazimirova M, Thangamani S, Bartikova P, Hermance M, Holikova V, Stibraniova I, et al. Tick-borne viruses and biological processes at the tick-host-virus interface. *Front Cell Infect Microbiol*. 2017;7:339. doi: 10.3389/fcimb.2017.00339 28798904 PMC5526847

[53] Gargili A, Estrada-Pena A, Spengler JR, Lukashev A, Nuttall PA, Bente DA. The role of ticks in the maintenance and transmission of Crimean-Congo hemorrhagic fever virus: A review of published field and laboratory studies. *Antiviral Res*. 2017;144:93–119. doi: 10.1016/j.antiviral.2017.05.010 28579441 PMC6047067

[54] Moraes-Filho J, Costa FB, Gerardi M, Soares HS, Labruna MB. *Rickettsia rickettsii* co-feeding transmission among *Amblyomma aureolatum* ticks. *Emerg Infect Dis*. 2018;24(11):2041–8. doi: 10.3201/eid2411.180451 30334709 PMC6200015

[55] Zhang X, Wang N, Wang Z, Che L, Chen C, Zhao WZ, et al. First fatal infection and phylodynamic analysis of severe fever with thrombocytopenia syndrome virus in Jilin Province, northeastern China. *Virol Sin*. 2021;36(2):329–32. doi: 10.1007/s12250-020-00228-z 32458298 PMC8087733

[56] Liu H, Li Z, Wang Z, He B, Wang S, Wei F, et al. The first molecular evidence of severe fever with thrombocytopenia syndrome virus in ticks in Jilin, Northeastern China. *Ticks Tick Borne Dis*. 2016;7(6):1280–3. doi: 10.1016/j.ttbdis.2016.06.007 27460903

[57] Zhang L, Li S, Huang SJ, Wang ZD, Wei F, Feng XM, et al. Isolation and genomic characterization of lymphocytic choriomeningitis virus in ticks from northeastern China. *Transbound Emerg Dis*. 2018;65(6):1733–9. doi: 10.1111/tbed.12946 29992783

