# Supplementary material for: Extensive diversity of RNA viruses in ticks revealed by metagenomics in northeastern China
Source: PLoS Negl Trop Dis. 2022 Dec 21;16(12):e0011017. doi: 10.1371/journal.pntd.0011017 (PMC9836300; doi:10.1371/journal.pntd.0011017)
Supplement: S1 Table — (DOCX) [file pntd.0011017.s001.docx]

S1 Table. Summary of sample collection and library, and RNA sequencing.

| Sampling of ticks | | | | |  |  | Library |  | NO. of reads by RNA-seq | |
| --- | --- | --- | --- | --- | --- | --- | --- | --- | --- | --- |
| Natural foci | **Location** | **Species** | **No. of ticks** | **Host** | **Sex** |  | **Library name** |  | **Total (no-rRNA, clean reads)** | **Viral** |
| Changbai mountain | Ji'an | *Haemaphysalis japonica* | 100 | free | mix |  | JA |  | 19,508,058 | 102,684 |
|  | Dunhua | *Haemaphysalis japonica* | 104 | free | mix |  | DH1 |  | 19,029,599 | 89,981 |
|  |  | *Dermacentor silvarum* | 86 | free | mix |  | DH2 |  | 33,462,658 | 70,723 |
|  |  | *Ixodes persulcatus* | 192 | free | mix |  | DH3 |  | 33,874,956 | 22,778 |
|  | Shulan | *Dermacentor silvarum* | 100 | cattle | Female |  | ShL1 |  | 17,980,248 | 14,487 |
|  |  |  | 100 | cattle | Male |  | ShL2 |  | 24,729,897 | 32,472 |
|  |  |  | 100 | cattle | Female |  | ShL3 |  | 29,225,664 | 15,631 |
|  | Fangzheng | *Ixodes persulcatus* | 100 | free | Female |  | FZ2 |  | 23,958,841 | 12,529 |
|  |  | *Ixodes persulcatus* | 100 | free | Male |  | FZ3 |  | 21,234,730 | 13,524 |
|  |  | *Haemaphysalis conicinna* | 100 | free | mix |  | FZ1 |  | 28,460,045 | 166,572 |
|  | Mudanjiang | *Ixodes persulcatus* | 56 | free | mix |  | MDJ2 |  | 16,505,918 | 12,861 |
|  |  | *Haemaphysalis conicinna* | 100 | free | mix |  | MDJ1 |  | 18,326,750 | 59,554 |
| Sub-total | | | **1,238** |  |  |  | **12** |  | **286,297,364** | **613,796** |
| Xiaoxing'an mountain | Yichun | *Haemaphysalis conicinna* | 30 | free | Male |  | YC1 |  | 7,593,327 | 28,443 |
|  |  | *Haemaphysalis conicinna* | 23 | free | Female |  | YC2 |  | 4,358,940 | 18,627 |
|  |  | *Ixodes persulcatus* | 100 | free | Male |  | YC3 |  | 27,918,817 | 18,667 |
|  |  | *Ixodes persulcatus* | 100 | free | Female |  | YC4 |  | 41,199,428 | 20,821 |
| Sub-total | | | **253** |  |  |  | **4** |  | **81,070,513** | **86,558** |
| Daxing'an mountain | Tahe | *Haemaphysalis conicinna* | 53 | free | Male |  | TH1 |  | 13,414,878 | 50,250 |
|  |  | *Haemaphysalis conicinna* | 63 | free | Female |  | TH2 |  | 11,939,706 | 51,022 |
|  |  | *Ixodes persulcatus* | 100 | free | Male |  | TH3 |  | 25,344,899 | 26,625 |
|  |  | *Ixodes persulcatus* | 100 | free | Female |  | TH4 |  | 37,612,741 | 67,194 |
|  | Songling | *Haemaphysalis conicinna* | 14 | free | Male |  | SL1 |  | 3,543,553 | 13,274 |
|  |  | *Haemaphysalis conicinna* | 10 | free | Female |  | SL2 |  | 1,895,191 | 8,099 |
|  |  | *Ixodes persulcatus* | 100 | free | Male |  | SL3 |  | 17,014,736 | 2,696 |
|  |  | *Ixodes persulcatus* | 100 | free | Female |  | SL4 |  | 37,646,572 | 10,513 |
| Sub-total | | | **540** |  |  |  | **8** |  | **148,412,276** | **229,672** |
| Total | | | **2,031** |  |  |  | **24** |  | **515,780,153** | **930,026** |
