# Supplementary material for: Extensive diversity of RNA viruses in ticks revealed by metagenomics in northeastern China
Source: PLoS Negl Trop Dis. 2022 Dec 21;16(12):e0011017. doi: 10.1371/journal.pntd.0011017 (PMC9836300; doi:10.1371/journal.pntd.0011017)
Supplement: S2 Table — (DOCX) [file pntd.0011017.s002.docx]

S2 Table. Reference viruses used in the present study.

| Family | | Genus | Virus | Strain | GenBank accession No. |
| --- | --- | --- | --- | --- | --- |
| *Flaviviridae* | | | | | |
|  | *Flaviviridae* | *Unclassified* | Amblyomma virus | GXTV108 | AVL26136 |
|  | *Flaviviridae* | *Unclassified* | Mogiana tick virus | Yunnan2016 | QJQ38165 |
|  | *Flaviviridae* | *Unclassified* | Jingmen tick virus | SY84 | YP_009029999 |
|  | *Flaviviridae* | *Unclassified* | Kindia tick virus | 2017/1 | QCW07567 |
|  | *Flaviviridae* | *Unclassified* | Yanggou tick virus | YG | QBQ65056 |
|  | *Flaviviridae* | *Unclassified* | Alongshan virus | H3 | AXE71873 |
|  | *Flaviviridae* | *Unclassified* | Thrips tabaci associated jingmen like virus 1 | THR-E_DN19401 | QNS31061 |
|  | *Flaviviridae* | *Unclassified* | Guaico Culex virus | LO35 | AKL90460 |
|  | *Flaviviridae* | *Unclassified* | Wuhan cricket virus | WHXS-1 | YP_009179405 |
|  | *Flaviviridae* | *Unclassified* | Wuhan aphid virus 1 | WHYC-1 | YP_009179388 |
|  | *Flaviviridae* | *Unclassified* | Wuhan flea virus | WHZM | YP_009179403 |
|  | *Flaviviridae* | *Flavivirus* | Aedes flavivirus | Narita-21 | YP_003029843 |
|  | *Flaviviridae* | *Flavivirus* | Tick-born encephalitis virus | QLL99543 |  |
|  | *Flaviviridae* | *Flavivirus* | Dengue virus 1 | 45AZ5 | NP_059433 |
|  | *Flaviviridae* | *Flavivirus* | Japanese encephalitis virus | - | NP_059434 |
|  | *Flaviviridae* | *Flavivirus* | Zika virus | MR 766 | YP_002790881 |
|  | *Flaviviridae* | *Pestivirus* | Classical swine fever virus | Eystrup | NP_075354 |
|  | *Flaviviridae* | *Pestivirus* | Norway rat pestivirus | NrPV/NYC-D23 | YP_009109567 |
|  | *Flaviviridae* | *Pestivirus* | Atypical porcine pestivirus 1 | Bavaria S5/9 | YP_009268709 |
|  | *Flaviviridae* | *Unclassified* | Xingshan cricket virus | XSXS-2 | YP_009179220 |
|  | *Flaviviridae* | *Unclassified* | Bole tick virus 4 | BLP-1 | YP_009179221 |
|  | *Flaviviridae* | *Hepacivirus* | Norway rat hepacivirus 1 | NrHV-1/NYC-C12 | YP_009109557 |
|  | *Flaviviridae* | *Hepacivirus* | Equine hepacivirus | JPN3 | YP_009058898 |
|  | *Flaviviridae* | *Hepacivirus* | Hepatitis C virus | H77 | NP_671491 |
|  | *Flaviviridae* | *Hepacivirus* | Chinese softshell turtle hepacivirus | WHJYGF75270 | AVM87613 |
|  | *Flaviviridae* | *Pegivirus* | Equine Pegivirus 1 | C0035 | YP_007697649 |
|  | *Flaviviridae* | *Pegivirus* | Pegivirus A | - | NP_045010 |
|  | *Flaviviridae* | *Unclassified* | Alongshan virus | H3 | MH158417 |
|  | *Flaviviridae* | *Unclassified* | Alongshan virus | HLJ1 | MT246198 |
|  | *Flaviviridae* | *Unclassified* | Alongshan virus | HLJ2 | MT246199 |
|  | *Flaviviridae* | *Unclassified* | Alongshan virus | Miass527 | MN648771 |
|  | *Flaviviridae* | *Unclassified* | Alongshan virus | Miass502 | MW525316 |
|  | *Flaviviridae* | *Unclassified* | Alongshan virus | Miass519 | MN648775 |
|  | *Flaviviridae* | *Unclassified* | Alongshan virus | Miass506 | MW525320 |
|  | *Flaviviridae* | *Unclassified* | Alongshan virus | Kuutsalo-23 | MN107155 |
|  | *Flaviviridae* | *Unclassified* | Alongshan virus | Haapasaari-18 | MN107159 |
|  | *Flaviviridae* | *Unclassified* | Takachi virus | IM-OI70 | LC628194 |
|  | *Flaviviridae* | *Unclassified* | Xinjiang tick virus 1 | XJO381 | MZ244282 |
|  | *Flaviviridae* | *Unclassified* | Yanggou tick virus | XJ-YGTV-1 | MT248420 |
|  | *Flaviviridae* | *Unclassified* | Jingmen Tick Virus | SY84 | NC_024114 |
|  | *Flaviviridae* | *Unclassified* | Heilongjiang tick virus | HLJ41 | MK721862 |
|  | *Flaviviridae* | *Unclassified* | Guangxi tick virus | GX46 | MK721858 |
|  | *Flaviviridae* | *Flavivirus* | Tick-born encephalitis virus | DXAL-21 | EU089980 |
|  | *Flaviviridae* | *Flavivirus* | Tick-born encephalitis virus | DXAL-12 | EU089977 |
|  | *Flaviviridae* | *Flavivirus* | Tick-born encephalitis virus | DXAL-13 | EU089976 |
|  | *Flaviviridae* | *Flavivirus* | Tick-born encephalitis virus | DXAL-16 | EU089978 |
|  | *Flaviviridae* | *Flavivirus* | Tick-born encephalitis virus | Primorye-94 | EU816454 |
|  | *Flaviviridae* | *Flavivirus* | Tick-born encephalitis virus | Primorye-91 | JQ825150 |
|  | *Flaviviridae* | *Flavivirus* | Tick-born encephalitis virus | Sofjin-Chumakov | KC806252 |
|  | *Flaviviridae* | *Flavivirus* | Tick-born encephalitis virus | SofjinKSY | JF819648 |
|  | *Flaviviridae* | *Flavivirus* | Tick-born encephalitis virus | JL jaohe | MT246197 |
|  | *Flaviviridae* | *Flavivirus* | Tick-born encephalitis virus | DXAL-18 | JQ650522 |
|  | *Flaviviridae* | *Flavivirus* | Tick-born encephalitis virus | HLB-T74 | MN615727 |
|  | *Flaviviridae* | *Flavivirus* | Tick-born encephalitis virus | DXAL-T83 | MN615728 |
|  | *Flaviviridae* | *Flavivirus* | Tick-born encephalitis virus | MDJ-01 | JQ650522 |
|  | *Flaviviridae* | *Flavivirus* | Tick-born encephalitis virus | MDJ-03 | JF316708 |
|  | *Flaviviridae* | *Flavivirus* | Tick-born encephalitis virus | MDJ-02 | JF316707 |
|  | *Flaviviridae* | *Flavivirus* | Tick-born encephalitis virus | Senzhang | JQ650523 |
|  | *Flaviviridae* | *Flavivirus* | Tick-born encephalitis virus | JL-T75 | MN615726 |
|  | *Flaviviridae* | *Flavivirus* | Tick-born encephalitis virus | JLCB11-40 | MF398820 |
|  | *Flaviviridae* | *Flavivirus* | Tick-born encephalitis virus | JLCB11-35 | MF398819 |
|  | *Flaviviridae* | *Flavivirus* | Tick-born encephalitis virus | JLCB11-08 | MF398818 |
|  | *Flaviviridae* | *Unclassified* | Bole tick virus 4 | 17-L2 | QBQ65091 |
|  | *Flaviviridae* | *Unclassified* | Bole tick virus 4 | bole4-xinjiang-JMN | QFR54187 |
|  | *Flaviviridae* | *Unclassified* | Bole tick virus 4 | BLP-1 | YP_009179221 |
|  | *Flaviviridae* | *Unclassified* | Bole tick virus 4 | GSC346flaviV | QYW06822 |
|  | *Flaviviridae* | *Unclassified* | Bole tick virus 4 | Bangali/H.truncatum/2018 | QSR83619 |
|  | *Flaviviridae* | *Unclassified* | Bole tick virus 4 | Iftin/H.dromedarii/2018 | QSR83621 |
|  | *Flaviviridae* | *Unclassified* | Bole tick virus 4 | TTP-Pool-4 | QDW81037 |
|  | *Flaviviridae* | *Unclassified* | Bole tick virus 4 | Thailand_tick_flavivirus | QFR36180 |
|  | *Flaviviridae* | *Unclassified* | Bole tick virus 4 | Iasi23 | QUJ17982 |
|  | *Flaviviridae* | *Unclassified* | Bole tick virus 4 | Iasi21 | QUJ17981 |
|  | *Flaviviridae* | *Unclassified* | Bole tick virus 4 | Iasi20 | QUJ17980 |
|  | *Flaviviridae* | *Unclassified* | Bole tick virus 4 | Iasi50 | QUJ17979 |
| *Nairoviridae* | | | | | |
|  | *Nairoviridae* | *Orthonairovirus* | Hazara virus | JC 280 | AAQ93049 |
|  | *Nairoviridae* | *Orthonairovirus* | Tofla virus | Toku_Hfla_2013 | YP_009227122 |
|  | *Nairoviridae* | *Orthonairovirus* | Nairobi sheep disease virus | Hubei | AYI99257 |
|  | *Nairoviridae* | *Orthonairovirus* | Dugbe virus | DUGV_DGT707 | ABY60378 |
|  | *Nairoviridae* | *Orthonairovirus* | Kupe virus | Kupe_225 | ACJ26836 |
|  | *Nairoviridae* | *Orthonairovirus* | Crimean Congo hemorrhagic fever virus | 813042 UAE | ASW20659 |
|  | *Nairoviridae* | *Orthonairovirus* | Thiafora virus | AnD 11411 | YP_009513191 |
|  | *Nairoviridae* | *Orthonairovirus* | Erve virus | Brest/An 221 (TVP21049) | AMT75395 |
|  | *Nairoviridae* | *Orthonairovirus* | Taggert virus | OTU36.IU7 | QKK82908 |
|  | *Nairoviridae* | *Orthonairovirus* | Tillamook virus | RML 86 | AMT75431 |
|  | *Nairoviridae* | *Orthonairovirus* | Clo Mor virus | SCOT Ar7 | AMT75386 |
|  | *Nairoviridae* | *Orthonairovirus* | Artashat virus | LEIV-10898Az | YP_009666119 |
|  | *Nairoviridae* | *Orthonairovirus* | Keterrah virus | P61361 | YP_009361838 |
|  | *Nairoviridae* | *Orthonairovirus* | Gossas virus | DakAnD 401 | ALD83626 |
|  | *Nairoviridae* | *Orthonairovirus* | Qalyub virus | EgAr 370 | AKC89319 |
|  | *Nairoviridae* | *Orthonairovirus* | Leopards’ Hill virus | 11SB17 | YP_009111284 |
|  | *Nairoviridae* | *Orthonairovirus* | Yogue virus | DakAnD 56 | YP_009246486 |
|  | *Nairoviridae* | *Orthonairovirus* | Vinegar Hill virus | CS1499 | AUD40046 |
|  | *Nairoviridae* | *Orthonairovirus* | Abu Hammad virus | Art 1194 | AMT75371 |
|  | *Nairoviridae* | *Orthonairovirus* | Estero Real virus | K329 | AXP33563 |
|  | *Nairoviridae* | *Orthonairovirus* | Soldado virus | TRVL 52214 | AMT75425 |
|  | *Nairoviridae* | *Orthonairovirus* | Raza virus | 829 | AMT75416 |
|  | *Nairoviridae* | *Orthonairovirus* | Hughes orthonairovirus | G2126 | AMT75407 |
|  | *Nairoviridae* | *Orthonairovirus* | Tamdy virus | XJ01 | QFU19352 |
|  | *Nairoviridae* | *Orthonairovirus* | Tacheng Tick Virus 1 | TC253 | YP_009304986 |
|  | *Nairoviridae* | *Orthonairovirus* | Songling virus | HLJ1202 | QPO14991 |
|  | *Nairoviridae* | *Sabavirus* | South Bay virus | SBV-H-1 | AII01810 |
|  | *Nairoviridae* | *Norwavirus* | Grotenhout virus | Gierle-1 | ARB16032 |
|  | *Nairoviridae* | *Norwavirus* | Norway nairovirus 1 | NOR/B1V/Tofte/2014 | ASY03236 |
|  | *Nairoviridae* | *Unclassified* | Beiji nariovirus | H59 | UFP37782 |
|  | *Nairoviridae* | *Shaspivirus* | Shayang Spider Virus 1 | SYZZ-4 | YP_009300680 |
|  | *Nairoviridae* | *Striwavirus* | Sanxia Water Strider Virus 1 | SXSSP08 | YP_009293594 |
|  | *Nairoviridae* | *Orthonairovirus* | Songling virus | YC585 | MT328780 |
|  | *Nairoviridae* | *Orthonairovirus* | Songling virus | HLJ1202 | MT328777 |
|  | *Nairoviridae* | *Orthonairovirus* | Tacheng tick virus 1 | TC253 | NC_031286 |
|  | *Nairoviridae* | *Unclassified* | Shanxi tick virus 2 | SXO338nairoV | MZ244237 |
|  | *Nairoviridae* | *Unclassified* | Henan tick virus | HNO321nairoV | MZ244225 |
|  | *Nairoviridae* | *Orthonairovirus* | Beiji nariovirus | H56 | MW315111 |
|  | *Nairoviridae* | *Orthonairovirus* | Beiji nariovirus | H59 | MW315110 |
|  | *Nairoviridae* | *Orthonairovirus* | Beiji nariovirus | YKS44 | MN122080 |
|  | *Nairoviridae* | *Orthonairovirus* | Beiji nariovirus | H160 | MW315107 |
|  | *Nairoviridae* | *Orthonairovirus* | Beiji nariovirus | H39 | MW315109 |
|  | *Nairoviridae* | *Orthonairovirus* | Beiji nariovirus | H801 | MW315108 |
|  | *Nairoviridae* | *Orthonairovirus* | Gakugsa tick virus | Rus/lx persulcatus/Karelia/1/2018 | MN542363 |
|  | *Nairoviridae* | *Orthonairovirus* | Beiji nariovirus | H1063 | MW315112 |
|  | *Nairoviridae* | *Orthonairovirus* | Norway nairovirus 1 | NOR/H3/Skanevik/2014 | MF141041 |
|  | *Nairoviridae* | *Norwavirus* | Grotenhout virus | Gierle-1 | KY700683 |
|  | *Nairoviridae* | *Orthonairovirus* | Pustyn virus | Rus/lx ricinus/Moscow/2018 | MN542361 |
| *Phenuiviridae* | | | | | |
|  | *Phenuiviridae* | *Bandavirus* | FTLS virus | 2011YXX9 | AHE38327 |
|  | *Phenuiviridae* | *Bandavirus* | Dabie bandavirus | HB2012-177 | QQZ00259 |
|  | *Phenuiviridae* | *Bandavirus* | Orthobunyavirus BX-2010/Henan/CHN | 69 | AEO51773 |
|  | *Phenuiviridae* | *Bandavirus* | Huaiyangshan virus | XCQ-A112L | AFB82724 |
|  | *Phenuiviridae* | *Bandavirus* | Phlebovirus XLL/China/2009 | S1 | ADZ95575 |
|  | *Phenuiviridae* | *Bandavirus* | Guertu virus | DXM | ALQ33265 |
|  | *Phenuiviridae* | *Bandavirus* | Heartland virus | Patient1 | YP_009047242 |
|  | *Phenuiviridae* | *Bandavirus* | Zwiesel bat banyangvirus | ZV2011 | QHU78994 |
|  | *Phenuiviridae* | *Bandavirus* | Lone Star virus | TMA 1381 | YP_008003507 |
|  | *Phenuiviridae* | *Bandavirus* | Bhanja virus | ibAr2709 | YP_009141013 |
|  | *Phenuiviridae* | *Bandavirus* | Razdan bandavirus | LEIV-Arm2741 | YP_008719916 |
|  | *Phenuiviridae* | *Phlebovirus* | Mukawa phlebovirus | MKW73 | LC063770 |
|  | *Phenuiviridae* | *Phlebovirus* | Kuriyama virus | CZCT80Q | LC133178 |
|  | *Phenuiviridae* | *Phlebovirus* | Sand fever Naples-like virus | Poona | AEL29673 |
|  | *Phenuiviridae* | *Phlebovirus* | Adana virus | 195 | YP_009227127 |
|  | *Phenuiviridae* | *Phlebovirus* | Cacao virus | VP-437R | QCI62731 |
|  | *Phenuiviridae* | *Phlebovirus* | Rift Valley fever virus | ZH-548 | YP_003848704 |
|  | *Phenuiviridae* | *Phlebovirus* | Itaporanga virus | original | QCI62749 |
|  | *Phenuiviridae* | *Uukuvirus* | Bole Tick Virus 1 | BL075 | AJG39234 |
|  | *Phenuiviridae* | *Uukuvirus* | Brown dog tick phlebovirus 2 | TTP-Pool-5 | QDW81040 |
|  | *Phenuiviridae* | *Uukuvirus* | Lihan tick virus | LH-1 | AJG39242 |
|  | *Phenuiviridae* | *Uukuvirus* | Rhipicephalus associated phlebovirus 1 | YNTV3 | QCB64646 |
|  | *Phenuiviridae* | *Uukuvirus* | Tacheng Tick Virus 2 | TC252 | YP_010086229 |
|  | *Phenuiviridae* | *Uukuvirus* | Changping Tick Virus 1 | CP1-2 | AJG39235 |
|  | *Phenuiviridae* | *Uukuvirus* | Kabuto mountain virus | T32 | YP_009449450 |
|  | *Phenuiviridae* | *Uukuvirus* | Precarious point virus | - | AEL29680 |
|  | *Phenuiviridae* | *Uukuvirus* | Huangpi Tick Virus 2 | H114-17 | YP_009293590 |
|  | *Phenuiviridae* | *Uukuvirus* | Dabieshan Tick Virus | D3 | AJG39236 |
|  | *Phenuiviridae* | *Uukuvirus* | Yongjia Tick Virus 1 | YJ1-1 | AJG39274 |
|  | *Phenuiviridae* | *Uukuvirus* | Okutama tick virus | 17ISK-T8 | BBK20268 |
|  | *Phenuiviridae* | *Ixovirus* | Onega tick phlebovirus | Rus/Ix_persulcatus/Karelia/3/2018 | MN542366 |
|  | *Phenuiviridae* | *Ixovirus* | Blacklegged tick phlebovirus 1 | SC2 | ANT80544 |
|  | *Phenuiviridae* | *Ixovirus* | Sara tick phlebovirus | Rus/Ix_persulcatus/Karelia/4/2018 | MN542367 |
|  | *Phenuiviridae* | *Horwuvirus* | Whenzhou Shrimp Virus 1 | BJDX-5 | YP_009304989 |
|  | *Phenuiviridae* | *Wenrivirus* | Wuhan horsefly Virus | JJ2-1 | YP_009305136 |
|  | *Phenuiviridae* | *Tenuivirus* | Melon chlorotic spot virus | E11-018 | YP_009551587 |
|  | *Phenuiviridae* | *Tenuivirus* | Rice stripe virus | T | NP_620522 |
|  | *Phenuiviridae* | *Hudovirus* | Hubei lepidoptera virus 1 | LCM141331 | YP_009330283 |
|  | *Phenuiviridae* | *Pidchovirus* | Pidgey bunyavirus M6 | M6 | YP_009666272 |
|  | *Phenuiviridae* | *Hudivirus* | Hubei diptera virus 4 | SCM94992 | YP_009330281 |
|  | *Phenuiviridae* | *Beidivirus* | Hubei diptera virus 3 | SCM17647 | YP_009329894 |
|  | *Phenuiviridae* | *Phasivirus* | Phasi Charoen-like virus | Rio | YP_009505332 |
|  | *Phenuiviridae* | *Phasivirus* | Badu virus | TS6347 | YP_009505327 |
|  | *Phenuiviridae* | *Goukovirus* | Gouleako virus | A5/CI/2004 | YP_009664621 |
|  | *Phenuiviridae* | *Goukovirus* | Cumuto virus | TR7904 | YP_009664615 |
|  | *Phenuiviridae* | *Mobuvirus* | Mothra bunyavirus | JG1 | YP_009666266 |
|  | *Phenuiviridae* | *Rubodvirus* | Apple rubbery wood virus 1 | 982-11 | AWC67511 |
|  | *Phenuiviridae* | *Rubodvirus* | Apple rubbery wood virus 2 | 982-11 | AWC67514 |
|  | *Phenuiviridae* | *Entovirus* | Entoleuca phenui like virus 1 | E115-5 | AVD68666 |
|  | *Phenuiviridae* | *Lentinuvirus* | Lentinula edodes negative-strand RNA virus 2 | HG3 | BBI93118 |
|  | *Phenuiviridae* | *Laulavirus* | Laurel Lake virus | RTS65 | YP_009667028 |
|  | *Phenuiviridae* | *Coguvirus* | Citrus concave gum associated virus | CGW2 | YP_009422199 |
|  | *Peribunyaviridae* | *Unclassified* | Kuriyama virus | CZCT80Q Japan | LC133180 |
|  | *Phenuiviridae* | *Ixovirus* | Sara tick phlebovirus Russia | Rus/Ix_persulcatus/Karelia/4/2018 | MN542367 |
|  | *Phenuiviridae* | *Ixovirus* | Norway phlebovirus 1 Norway | NOR/A2/Bronnoya/2014 | NC_055433 |
|  | *Phenuiviridae* | *Ixovirus* | Blacklegged tick phlebovirus-1 USA | H12 | KM048314 |
|  | *Phenuiviridae* | *Ixovirus* | Onega tick phlebovirus Russia | Rus/Ix_persulcatus/Karelia/3/2018 | MN542365 |
| *Rhabdoviridae* | | | | | |
|  | *Rhabdoviridae* | *Perhabdovirus* | Perch rhabdovirus | PRV | YP_007641367 |
|  | *Rhabdoviridae* | *Unclassified* | Siniperca chuatsi rhabdovirus | - | YP_802942 |
|  | *Rhabdoviridae* | *Caligrhavirus* | Caligus rogercresseyi rhabdovirus | CrRV-Ch01 | YP_009666516 |
|  | *Rhabdoviridae* | *Unclassified* | Wuhan redfin culter dimarhabodovirus | DSYS6218 | AVM87289 |
|  | *Rhabdoviridae* | *Sprivivirus* | Carp sprivivirus | isolate=ADC-SVC2016-1 | AXA12039 |
|  | *Rhabdoviridae* | *Vesiculovirus* | Vesicular stomatitis New Jersey virus | NJ03CPB | QCF24424 |
|  | *Rhabdoviridae* | *Vesiculovirus* | American bat vesiculovirus | liver2008 | YP_008767243 |
|  | *Rhabdoviridae* | *Ledantevirus* | Yongjia Tick Virus 2 | YJ1-2 | YP_009305122 |
|  | *Rhabdoviridae* | *Ledantevirus* | Fikirini bat rhabdovirus | KEN352 | YP_009094022 |
|  | *Rhabdoviridae* | *Ohlsrhavirus* | North Creek Virus | 954 | AGY80343 |
|  | *Rhabdoviridae* | *Sigmavirus* | Drosophila obscura sigmavirus | 10A | YP_008686601 |
|  | *Rhabdoviridae* | *Unclassified* | Wuhan Fly Virus 2 | SYY1-3 | YP_009304656 |
|  | *Rhabdoviridae* | *Unclassified* | Wuhan Insect virus 7 | WHYC02 | YP_009301743 |
|  | *Rhabdoviridae* | *Unclassified* | Gata virus | M4 | YP_009315878 |
|  | *Rhabdoviridae* | *Tupavirus* | Tupaia virus | - | YP_238534 |
|  | *Rhabdoviridae* | *Sunrhavirus* | Sunguru virus | Ug#41 | YP_009094441 |
|  | *Rhabdoviridae* | *Sripuvirus* | Sripur virus | 733646 | YP_009362218 |
|  | *Rhabdoviridae* | *Hapavirus* | Landjia virus | DakAnB769d | YP_009362143 |
|  | *Rhabdoviridae* | *Arurhavirus* | Aruac virus | TRVL9223 | AJR28310 |
|  | *Rhabdoviridae* | *Curiovirus* | Curionopolis virus | BeAr440009 | YP_009512988 |
|  | *Rhabdoviridae* | *Tibrovirus* | Tibrogargan virus | CS132 | YP_007641376 |
|  | *Rhabdoviridae* | *Ephemerovirus* | Bovine ephemeral fever virus | - | NP_065409 |
|  | *Rhabdoviridae* | *Alphanemrhavirus* | Xinzhou nematode virus 4 | XZSJSC65771 | YP_009344989 |
|  | *Rhabdoviridae* | *Unclassified* | Huangpi Tick Virus 3 | H124-2 | YP_009288322 |
|  | *Rhabdoviridae* | *Unclassified* | Manly virus | - | AYP67529 |
|  | *Rhabdoviridae* | *Unclassified* | Bole Tick Virus 2 | BL076 | YP_009287864 |
|  | *Rhabdoviridae* | *Unclassified* | Tacheng Tick Virus 3 | TC255 | YP_009304331 |
|  | *Rhabdoviridae* | *Unclassified* | Wuhan Tick Virus 1 | X78-2 | YP_009305117 |
|  | *Rhabdoviridae* | *Mousrhavirus* | Moussa virus | C23 | YP_009094143 |
|  | *Rhabdoviridae* | *Sawgrhavirus* | Sawgrass virus | 64A-1247 | AJR28510 |
|  | *Rhabdoviridae* | *Zarhavirus* | Zahedan rhabdovirus | Ar Teh 157764 | YP_009552805 |
|  | *Rhabdoviridae* | *Barhavirus* | Bahia Grande virus | TB4-1054 | AJR28545 |
|  | *Rhabdoviridae* | *Lostrhavirus* | Lone star tick rhabdovirus | TickAa42 | ALO28655 |
|  | *Rhabdoviridae* | *Almendravirus* | Puerto Almendras virus | LO-39 | YP_009094394 |
|  | *Rhabdoviridae* | *Lyssavirus* | Rabies lyssavirus | 02050CHI | APD77380 |
|  | *Rhabdoviridae* | *Lyssavirus* | Irkut lyssavirus | - | YP_007641401 |
|  | *Rhabdoviridae* | *Unclassified* | Fox fecal rhabdovirus | S40 | YP_009204560 |
|  | *Rhabdoviridae* | *Unclassified* | Beihai dimarhabodovirus 1 | BHFishS58819 | AVM87284 |
|  | *Rhabdoviridae* | *Unclassified* | Fujian dimarhabdovirus | BHNC4885 | AVM87298 |
|  | *Rhabdoviridae* | *Unclassified* | Wuhan House Fly Virus 2 | SYY4-5 | YP_009304985 |
|  | *Rhabdoviridae* | *Unclassified* | Wuhan Ant Virus | WHMY02 | YP_009304559 |
|  | *Rhabdoviridae* | *Unclassified* | Tacheng Tick Virus 7 | TCRP-3 | YP_009304476 |
|  | *Rhabdoviridae* | *Varicosavirus* | Lettuce big vein associated virus | - | YP_002308576 |
|  | *Rhabdoviridae* | *Cytorhabdovirus* | Lettuce yellow mottle virus | - | YP_002308376 |
|  | *Rhabdoviridae* | *Cytorhabdovirus* | Wuhan Insect virus 5 | YCYC02 | YP_009300875 |
|  | *Rhabdoviridae* | *Alphanucleorhabdovirus* | Rice yellow stunt virus | - | NP_620502 |
|  | *Rhabdoviridae* | *Dichorhavirus* | Orchid fleck dichorhavirus | So | YP_001294929 |
|  | *Rhabdoviridae* | *Gammanucleorhabdovirus* | Maize fine streak virus | - | YP_052849 |
|  | *Rhabdoviridae* | *Betanucleorhabdovirus* | Sonchus yellow net virus | DSMZ PV-0052 | QTC11065 |
|  | *Rhabdoviridae* | *Unclassified* | Manly virus | - | AYP67529 |
|  | *Rhabdoviridae* | *Unclassified* | Bole Tick Virus 2 | BL076 | YP009287864 |
|  | *Rhabdoviridae* | *Unclassified* | Tacheng Tick Virus 3 | TC255 | YP_009304331 |
|  | *Rhabdoviridae* | *Unclassified* | Wuhan Tick Virus 1 | X78-2 | YP_009305117 |
|  | *Rhabdoviridae* | *Unclassified* | Huangpi Tick Virus 3 | H124-2 | YP_009288322 |
|  | *Rhabdoviridae* | *Unclassified* | Norway mononegavirus 1 | NOR/H3/Skanevik/2014 | ASY03261 |
| *Chuviridae* | | | | | |
|  | *Chuviridae* | *Mivirus* | Deer tick mononegavirales-like virus | DTM1 | [AIE42676](https://www.ncbi.nlm.nih.gov/protein/667843479) |
|  | *Chuviridae* | *Mivirus* | Suffolk virus | FI3 | [AIY53910](https://www.ncbi.nlm.nih.gov/protein/726973166) |
|  | *Chuviridae* | *Mivirus* | Umea virus | OTU2.IU18 | [QKK82916](https://www.ncbi.nlm.nih.gov/protein/1851267830) |
|  | *Chuviridae* | *Mivirus* | Genoa virus | - | [AYP67566](https://www.ncbi.nlm.nih.gov/protein/1503327968) |
|  | *Chuviridae* | *Mivirus* | Bole Tick Virus 3 | 15-XJL | [QBQ65109](https://www.ncbi.nlm.nih.gov/protein/1603483498) |
|  | *Chuviridae* | *Mivirus* | Changping Tick Virus 2 | CP1-4 | [AJG39044](https://www.ncbi.nlm.nih.gov/protein/752455496) |
|  | *Chuviridae* | *Mivirus* | Karukera tick virus | GM | [QGW51122](https://www.ncbi.nlm.nih.gov/protein/1783519034) |
|  | *Chuviridae* | *Mivirus* | Changping mivirus | Thailand tick chuvirus 2 | [QFR36194](https://www.ncbi.nlm.nih.gov/protein/1768400164) |
|  | *Chuviridae* | *Mivirus* | Mivirus sp | TTP-Pool-7 | [QDW81054](https://www.ncbi.nlm.nih.gov/protein/1714017572) |
|  | *Chuviridae* | *Mivirus* | Lonestar tick chuvirus 1 | RTS21 | [ANC97697](https://www.ncbi.nlm.nih.gov/protein/1026473212) |
|  | *Chuviridae* | *Mivirus* | Wuhan mivirus | Thailand tick chuvirus 1 | [QFR36197](https://www.ncbi.nlm.nih.gov/protein/1768400168) |
|  | *Chuviridae* | *Mivirus* | Wuhan tick virus 2 | WTV2_9 | [AYV61054](https://www.ncbi.nlm.nih.gov/Taxonomy/Browser/wwwtax.cgi?id=1608138) |
|  | *Chuviridae* | *Mivirus* | Tacheng Tick Virus 5 | TC254 | [AJG39058](https://www.ncbi.nlm.nih.gov/protein/752455516) |
|  | *Chuviridae* | *Mivirus* | Changping Tick Virus 3 | CP1-3 | [AJG39047](https://www.ncbi.nlm.nih.gov/protein/752455500) |
|  | *Chuviridae* | *Boscovirus* | Wuhan Louse Fly Virus 6 | BFJSC-2 | [AJG39070](https://www.ncbi.nlm.nih.gov/protein/752455534) |
|  | *Chuviridae* | *Boscovirus* | Wuhan Louse Fly Virus 7 | BFJSC-3 | [AJG39073](https://www.ncbi.nlm.nih.gov/protein/752455540) |
|  | *Chuviridae* | *Unclassified* | Wenling chuvirus-like virus 1 | WLJQ101487 | [APG78818](https://www.ncbi.nlm.nih.gov/protein/1110867207) |
|  | *Chuviridae* | *Demapteravirus* | Dermapteran chu-related virus | OKIAV142 | [QMP82301](https://www.ncbi.nlm.nih.gov/protein/1882567525) |
|  | *Chuviridae* | *Unclassified* | Solanum melongena chuvirus-like virus_ | pt065-lat-7-plant_65_ | QKI28861 |
|  | *Chuviridae* | *Odonatavirus* | Hubei odonate virus 11 | DLQTFY32 | [UHK03003](https://www.ncbi.nlm.nih.gov/protein/2168954766) |
|  | *Chuviridae* | *Nigecruvirus* | Blacklegged tick chuvirus-2 | RTS126 | AUW34382 |
|  | *Chuviridae* | *Piscichuvirus* | Wenling fish chuvirus-like virus | XYXMG8676 | AVM87278 |
|  | *Chuviridae* | *Piscichuvirus* | Wenling fish chu-like virus | XQTMS36511 | AVM87275 |
|  | *Chuviridae* | *Piscichuvirus* | Guangdong red-banded snake chuvirus-like virus | LPSC27055 | AVM87272 |
|  | *Chuviridae* | *Piscichuvirus* | Herr Frank virus 1 | 481-18_HFrV-1 | QHX39772 |
|  | *Chuviridae* | *Piscichuvirus* | Sanxia atyid shrimp virus 4 | SXXX37205 | APG78770 |
|  | *Chuviridae* | *Chuvivirus* | Wengzhou Crab virus 2 | ZCX13 | AJG39060 |
|  | *Chuviridae* | *Chuvivirus* | Wenling chuvirus-like virus 2 | WLJQ104274 | APG78831 |
|  | *Chuviridae* | *Chuvivirus* | Wenling Crustacean virus 13 | WLJQ104251 | APG78828 |
|  | *Chuviridae* | *Chuvivirus* | Wenling Crustacean virus 14 | WLJQ104130 | APG78824 |
|  | *Chuviridae* | *Scarabeuvirus* | Wuchang Cockroach virus 3 | WCZL-1 | AJG39067 |
|  | *Chuviridae* | *Scarabeuvirus* | Lishi Spider virus 1 | LSZZ11 | AJG39051 |
|  | *Chuviridae* | *Scarabeuvirus* | Lampryis noctiluca chuvirus-like virus 1 | 17FIN7 | QBP37027 |
|  | *Chuviridae* | *Scarabeuvirus* | Hubei chuvirus-like virus 3 | QTM26698 | APG78724 |
|  | *Chuviridae* | *Scarabeuvirus* | Hubei chuvirus-like virus 1 | QTM26249 | APG78716 |
|  | *Chuviridae* | *Pterovirus* | Hymenopteran chu-related virus | OKIAV147 | QPB73971 |
|  | *Chuviridae* | *Unclassified* | Atrato Chu-like virus 1 | Cqvz_1753-8 | QHA33917 |
|  | *Chuviridae* | *Doliuvirus* | Chuvirus Mos8Chu0 | Mos8Chu0 | API61887 |
|  | *Chuviridae* | *Culicidavirus* | Culex mosquito virus 4 | OTU50_ | [QGA70925](https://www.ncbi.nlm.nih.gov/protein/1773036533) |
|  | *Chuviridae* | *Culicidavirus* | Culex mosquito virus 5 | CMosV5Santa | AXQ04841 |
|  | *Chuviridae* | *Culicidavirus* | Imjin River virus 1 | A12.2496ROK2012 | ALP32028 |
|  | *Chuviridae* | *Culicidavirus* | Wuhan Mosquito virus 8 | XC2-7 | AJG39074 |
|  | *Chuviridae* | *Morsusvirus* | Tacheng Tick Virus 4 | TCRP-1 | AJG39057 |
|  | *Chuviridae* | *Unclassified* | Xinzhou nematode virus 5 | XZSJSC65765 | APG78852 |
|  | *Chuviridae* | *Taceavirus* | Wenling crustacean virus 15 | WLJQ91782 | APG78840 |
|  | *Chuviridae* | *Pediavirus* | Beihai barnacle virus 9 | BHTH10927 | APG78655 |
|  | *Unclassified* | *Unclassified* | Schistocephalus solidus jingchuvirus | SsJV-SsAE | QJD26154 |
|  | *Myriaviridae* | *Myriavirus* | Hubei myriapoda virus 8 | WGML66308 | APG78798 |
|  | *Natareviridae* | *Charybdivirus* | Wenzhou Crab Virus 3 | RBX9 | AJG39066 |
|  | *Chuviridae* | *Mivirus* | Beihai hermit crab virus 3 | BHJJX21702 | APG78635 |
|  | *Chuviridae* | *Unclassified* | Atrato Chu-like virus 4 | Wy_1731-1 | QHA33906 |
|  | *Unclassified* | *Unclassified* | Shuangao Insect Virus 5 | QSA09 | QHA33675 |
|  | *Chuviridae* | *Mivirus* | Shuangao Lacewing Virus | QSA01 | AJG39082 |
|  | *Aliusviridae* | *Obscuruvirus* | Atrato Chu-like virus 5 | Psal_1739-1 | AJG39083 |
|  | *Aliusviridae* | *Ollusvirus* | Hubei coleoptera virus 3 | QCM109726 | APG78687 |
|  | *Aliusviridae* | *Ollusvirus* | Taiyuan leafhopper virus | TY1 | AYN64867 |
|  | *Aliusviridae* | *Ollusvirus* | Hancheng leafhopper mivirus | Hancheng | QIH31161 |
|  | *Aliusviridae* | *Ollusvirus* | Culverton virus | AFV17 | QED21529 |
|  | *Aliusviridae* | *Ollusvirus* | Hymenopteran chu-related virus | OKIAV126 | QPB73970 |
|  | *Aliusviridae* | *Ollusvirus* | Scaldis River bee virus | S33-1 | APT68159 |
|  | *Chuviridae* | *Mivirus* | Nuomin virus | H141 | UKS70424 |
|  | *Chuviridae* | *Mivirus* | Nuomin virus | T43 | UKS70440 |
|  | *Chuviridae* | *Mivirus* | Lesnoe mivirus | Rus/Ix_persulcatus/Karelia/5/2018 | QPD01622 |
|  | *Chuviridae* | *Mivirus* | Nuomin virus | H160 | UKS70436 |
|  | *Chuviridae* | *Mivirus* | Nuomin virus | H159 | UKS70432 |
|  | *Chuviridae* | *Mivirus* | Nuomin virus | H145 | UKS70428 |
|  | *Chuviridae* | *Mivirus* | Nuomin virus | H43 | UKS70416 |
|  | *Chuviridae* | *Mivirus* | Nuomin virus | H109 | UKS70420 |
| *Partitiviridae* | | | | | |
|  | *Partitiviridae* | *Betapartitivirus* | White clover cryptic virus 2 | IPP_Lirepa | YP_007889821 |
|  | *Partitiviridae* | *Betapartitivirus* | Primula malacoides virus | China-Mar2007 | YP_003104768 |
|  | *Partitiviridae* | *Betapartitivirus* | Rosellinia necatrix partitivirus | 1-W8 | YP_392480 |
|  | *Partitiviridae* | *Betapartitivirus* | Pleurotus ostreatus virus 1 | - | YP_227355 |
|  | *Partitiviridae* | *Betapartitivirus* | Hubei partiti-like virus 23 | NS20201025 | QTM24142 |
|  | *Partitiviridae* | *Betapartitivirus* | Fusarium poae virus 1 | A11 | NP_624349 |
|  | *Partitiviridae* | *Betapartitivirus* | Rhizoctonia solani virus 717 | Rhizoctonia solani 717 partitivirus | NP_620659 |
|  | *Partitiviridae* | *Betapartitivirus* | Hubei partiti-like virus 24 | WHYY17134 | APG78329 |
|  | *Partitiviridae* | *Betapartitivirus* | Ceratocystis polonica partitivirus | CpPV-CMW7151 | YP_001911122 |
|  | *Partitiviridae* | *Betapartitivirus* | Atkinsonella hypoxylon partitivirus | 2H | NP_604475 |
|  | *Partitiviridae* | *Alphapartitivirus* | Rosellinia necatrix partitivirus 2 | W57 | YP_007419077 |
|  | *Partitiviridae* | *Alphapartitivirus* | Raphanus sativus cryptic virus 1 | - | YP_656506 |
|  | *Partitiviridae* | *Alphapartitivirus* | Sclerotinia sclerotiorum partitivirus S | - | YP_003082248 |
|  | *Partitiviridae* | *Alphapartitivirus* | Amasya cherry disease-associated mycovirus | - | YP_138537 |
|  | *Partitiviridae* | *Alphapartitivirus* | Rhizoctonia solani dsRNA virus 2 | GD11 | YP_009011230 |
|  | *Partitiviridae* | *Alphapartitivirus* | White clover cryptic virus 1 | - | YP_086754 |
|  | *Partitiviridae* | *Alphapartitivirus* | Beet cryptic virus 1 | - | YP_002308574 |
|  | *Partitiviridae* | *Unclassified* | Hubei partiti-like virus 59 | SCM33193 | APG78262 |
|  | *Partitiviridae* | *Unclassified* | Beihai barnacle virus 14 | BHTH35712 | APG78182. |
|  | *Partitiviridae* | *Unclassified* | Zygosaccharomyces bailii virus Z | - | NP_624325 |
|  | *Partitiviridae* | *Gammapartitivirus* | Botryotinia fuckeliana partitivirus 1 | - | YP_001686789 |
|  | *Partitiviridae* | *Gammapartitivirus* | Discula destructiva virus_1 | 247 | NP_116716 |
|  | *Partitiviridae* | *Gammapartitivirus* | Penicillium stoloniferum virus_S | - | YP_052856 |
|  | *Partitiviridae* | *Gammapartitivirus* | Gremmeniella abietina RNA virus | C5 | NP_659027 |
|  | *Partitiviridae* | *Gammapartitivirus* | Mycovirus | - | NP_624350 |
|  | *Partitiviridae* | *Gammapartitivirus* | Ustilaginoidea virens partitivirus 2 | Uv0901 | YP_008327312 |
|  | *Partitiviridae* | *Gammapartitivirus* | Penicillium stoloniferum virus F | - | YP_271922 |
|  | *Partitiviridae* | *Unclassified* | Jilin partiti-like virus 1 | JL/QG-4 | QTZ96786 |
|  | *Partitiviridae* | *Unclassified* | Norway partiti-like virus 1 | NOR/H3/Skanevik/2014 | ASY03271 |
|  | *Partitiviridae* | *Unclassified* | Beihai partiti-like virus 11 | BHWZXX14306 | APG78185 |
|  | *Partitiviridae* | *Unclassified* | Wuhan large pig roundworm virus 1 | WHZHC73278 | YP_009329866 |
|  | *Partitiviridae* | *Unclassified* | Xinzhou partiti-like virus 1 | XZSJSC65291 | YP_009329867 |
|  | *Partitiviridae* | *Deltapartitivirus* | Fig cryptic virus | BN13 | YP_004429258 |
|  | *Partitiviridae* | *Deltapartitivirus* | Rose cryptic virus 1 | ShB-1 | YP_001686786 |
|  | *Partitiviridae* | *Deltapartitivirus* | Raphanus sativus cryptic virus 2 | - | YP_001686783 |
|  | *Partitiviridae* | *Deltapartitivirus* | Raphanus sativus cryptic virus 3 | RasR7 | YP_002364401 |
|  | *Partitiviridae* | *Deltapartitivirus* | Hubei partiti-like virus 58 | 135 | QCM38725 |
|  | *Partitiviridae* | *Deltapartitivirus* | Persimmon cryptic virus | SSPI | YP_006390091 |
|  | *Partitiviridae* | *Unclassified* | Beihai barnacle virus 12 | BHTH16091 | YP_009333370 |
|  | *Partitiviridae* | *Unclassified* | Hubei diptera virus 18 | SCM37169 | YP_009329892 |
|  | *Partitiviridae* | *Unclassified* | Wuhan fly virus 5 | fly34516 | YP_009342458 |
|  | *Partitiviridae* | *Unclassified* | Beihai partiti-like virus 10 | YYSZX26933 | APG78357 |
|  | *Partitiviridae* | *Unclassified* | Wenzhou partiti-like virus 1 | WZRBX37312 | APG78352 |
|  | *Partitiviridae* | *Unclassified* | Hubei partiti-like virus 50 | spider58532 | APG78284 |
|  | *Partitiviridae* | *Unclassified* | Wuhan spider virus 10 | spider133290 | APG78279 |
|  | *Partitiviridae* | *Unclassified* | Hubei coleoptera virus 4 | QCM136227 | APG78220 |
|  | *Partitiviridae* | *Unclassified* | Hubei partiti-like virus 51 | WHWN52466 | APG78321 |
|  | *Partitiviridae* | *Unclassified* | Hubei partiti-like virus 52 | spider134151 | APG78280 |
|  | *Partitiviridae* | *Unclassified* | Hubei coleoptera virus 5 | QCM105830 | APG78219 |
|  | *Partitiviridae* | *Unclassified* | Hubei coleoptera virus 6 | QCM143720 | APG78221 |
|  | *Partitiviridae* | *Unclassified* | Jilin partiti-like virus 1 | JL/QG-4 | QTZ96786 |
|  | *Partitiviridae* | *Unclassified* | Jilin partiti-like virus 1 | JL/QG-1 | QTZ96785 |
|  | *Partitiviridae* | *Unclassified* | Jilin partiti-like virus 1 | JL/QG-2 | QTZ96784 |
|  | *Partitiviridae* | *Unclassified* | Norway partiti-like virus 1 | NOR/S5/Kilen/2014 | ASY03273 |
|  | *Partitiviridae* | *Unclassified* | Norway partiti-like virus 2 | NOR/H3/Skanevik/2014 | ASY03271 |
| *Tombusviridae* | | | | | |
|  | *Tombusviridae* | *Calvsuvirinae* | Groundut rosette virus | SRF57 | AVL26134 |
|  | *Tombusviridae* | *Calvsuvirinae* | Ethiopian tobacco bushy top virus | 18-2 | YP_009056849 |
|  | *Tombusviridae* | *Calvsuvirinae* | Carrot mottle virus | Weddel | YP_002302259 |
|  | *Tombusviridae* | *Calvsuvirinae* | Carrot mottle mimic virus | California | ACJ03572 |
|  | *Tombusviridae* | *Procedovirinae* | Potato necrosis virus | QV323 | ALF95240 |
|  | *Tombusviridae* | *Procedovirinae* | Galinsoga mosaic virus | - | CAA73863 |
|  | *Tombusviridae* | *Procedovirinae* | Pelargonium flower break virus | MZ10 | NP_945123 |
|  | *Tombusviridae* | *Procedovirinae* | Melon necrotic spot virus | Kochi | BAG13035 |
|  | *Tombusviridae* | *Procedovirinae* | Honeysuckle ringspot virus | California | YP_004191789 |
|  | *Tombusviridae* | *Procedovirinae* | Turnip crinkle virus | Helmstedt EPV_18_002 | QBG64840 |
|  | *Tombusviridae* | *Procedovirinae* | Cowpea mottle virus | - | NP_619521 |
|  | *Tombusviridae* | *Procedovirinae* | Pelargonium ringspot virus | DSMZ-PV-0304 | YP_009116639 |
|  | *Tombusviridae* | *Procedovirinae* | Panicum mosaic virus | Nebraska | AYG96552 |
|  | *Tombusviridae* | *Procedovirinae* | Beet black scorch virus | - | NP_758810 |
|  | *Tombusviridae* | *Procedovirinae* | Yam spherical virus | - | AHA53610 |
|  | *Tombusviridae* | *Procedovirinae* | Moroccan pepper virus | MPV-Cl | AXY96388 |
|  | *Tombusviridae* | *Procedovirinae* | Oat chlorotic stunt virus | - | NP_619751 |
|  | *Tombusviridae* | *Regressovirinae* | Sweet clover necrotic mosaic virus | 59 | NP_620674 |
|  | *Tombusviridae* | *Regressovirinae* | Red clover necrotic mosaic virus | Australia | NP_620523 |
|  | *Tombusviridae* | *Regressovirinae* | Carnation ringspot virus | PV-21 | BBI41284 |
|  | *Tombusviridae* | *Luteovirus* | Barley yellow dwarf virus kerII | K439 | YP_008083724 |
|  | *Tombusviridae* | *Luteovirus* | Red clover associated virus | HZ3 | AVX32322 |
|  | *Tombusviridae* | *Luteovirus* | Apple-associated luteovirus | A68 | YP_009551922 |
|  | *Tombusviridae* | *Luteovirus* | protein Soybean dwarf virus | RG24 | BDD37277 |
|  | *Tombusviridae* | *Unclassified* | Barns Ness breadcrumb sponge tombus-like virus2 | H89 | ASM94016 |
|  | *Tombusviridae* | *Unclassified* | Shahe isopoda virus 5 | SHWC0209c16708 | YP_009333221 |
|  | *Tombusviridae* | *Unclassified* | Corseley virus | Dsub_PoolSeq3 | AMO03229 |
|  | *Tombusviridae* | *Unclassified* | Sanxia water strider virus 14 | SXSSP2565 | YP_009337267 |
|  | *Tombusviridae* | *Unclassified* | Caledonia beadlet anemone tombus-like virus 1 | D78 | ASM94000 |
|  | *Tombusviridae* | *Unclassified* | Hubei mosquito virus 4 | mosHB232766 | APG76308 |
|  | *Tombusviridae* | *Unclassified* | Wenzhou tombus-like virus 11 | mosZJ33874 | YP_009342051 |
|  | *Tombusviridae* | *Unclassified* | Upmeje virus | OTU9.IU20 | QKK82929 |
|  | *Tombusviridae* | *Unclassified* | Wenling tombus-like virus 3 | WLJQ76752 | YP_009337225 |
|  | *Tombusviridae* | *Unclassified* | Beihai_tombus-like_virus 12 | BHTSS17936 | YP_009336526 |
|  | *Tombusviridae* | *Unclassified* | Hubei tombus-like virus 21 | spider121926 | YP_009336978 |
|  | *Tombusviridae* | *Unclassified* | Hubei tombus-like virus 22 | QTM26784 | YP_009336644 |
|  | *Tombusviridae* | *Unclassified* | Sanxia tombus-like virus 7 | SXSSP2567 | YP_009333294 |
|  | *Tombusviridae* | *Unclassified* | Upmeje virus OTU9.IU20 | OTU9.IU20 | MN830246 |
|  | *Tombusviridae* | *Unclassified* | Soybean thrips tombus-like virus 3 | STN1 | QQP18784 |
| *Solemoviridae* | | | | | |
|  | *Solemoviridae* | *Unclassified* | Norway luteo-like virus 3 | NOR/A2/Bronnoya/2014 | ASY03257 |
|  | *Solemoviridae* | *Unclassified* | Ixodes scapularris associated virus 1 | ISE6 | BBD75429 |
|  | *Solemoviridae* | *Unclassified* | American dog tick associated virus-1 | RTS-109 | AUX13126 |
|  | *Solemoviridae* | *Unclassified* | Xinjiang tick associated virus 1 | 14-YG | QBQ65103 |
|  | *Solemoviridae* | *Unclassified* | Lone star tick associated virus-1 | RTS-10 | AUX13123 |
|  | *Solemoviridae* | *Unclassified* | Hubei sobemo-like virus 15 | tick96090 | YP_009330030 |
|  | *Solemoviridae* | *Unclassified* | Hubei sobemo-like virus 11 | spider125434 | YP_009329992 |
|  | *Solemoviridae* | *Unclassified* | Rhodnius prolixus virus 6 | ICB_RJ | QYC92637 |
|  | *Solemoviridae* | *Unclassified* | Store beach virus | - | AYP67537 |
|  | *Solemoviridae* | *Unclassified* | Ixodes scapularis associated virus 2 | A1 | AII01812 |
|  | *Solemoviridae* | *Unclassified* | Norway luteo-like virus 2 | NOR/H3/Skanevik/2014 | ASY03255 |
|  | *Solemoviridae* | *Unclassified* | Jilin luteo-like virus 2 | JL-QG-2 | QTZ96787 |
|  | *Solemoviridae* | *Unclassified* | Shuangao insect virus 9 | insectZJ96499 | YP_009337273 |
|  | *Solemoviridae* | *Unclassified* | Baird Spence virus | UW | AOX15243 |
|  | *Solemoviridae* | *Unclassified* | Soybean thrips sobemo-like virus 7 | STN1SV7 | QQO81415 |
|  | *Solemoviridae* | *Unclassified* | Plasmopara viticola lesion associated sobemo-like 1 | DMS9-DN5046 | QHD64767 |
|  | *Solemoviridae* | *Unclassified* | Leuven Sobemo-like virus 1 | Vvul_virus_26 | QZZ63345 |
|  | *Solemoviridae* | *Unclassified* | Hubei sobemo-like virus 9 | spider112041 | YP_009329983 |
|  | *Solemoviridae* | *Unclassified* | Soybean thrips sobemo-like virus 9 | STN1SV9 | QQO81419 |
|  | *Solemoviridae* | *Unclassified* | Hubei sobemo-like virus 8 | QTM19779 | YP_009330088 |
|  | *Solemoviridae* | *Unclassified* | Nelson Sobemo-like virus 1 | Vvul_virus_62 | QZZ63405 |
|  | *Solemoviridae* | *Unclassified* | Blue fish point virus | Ixodes holocyclus | AYP67542 |
|  | *Solemoviridae* | *Unclassified* | Vespula vulgaris Sobemo-like virus 1 | Vvul_virus_03 | QZZ63305 |
|  | *Solemoviridae* | *Unclassified* | Hubei mosquito virus 2 | mosZJ35453 | YP_009337877 |
|  | *Solemoviridae* | *Unclassified* | Atrato Sobemo-like virus 5 | Cqvz 1772-143 | QHA33869 |
|  | *Solemoviridae* | *Unclassified* | Kwale mosquito virus | 18-Kenya-1 | QOI91445 |
|  | *Solemoviridae* | *Unclassified* | Guadeloupe mosquito virus | Ab-AAF-1-1 | QEM39253 |
|  | *Solemoviridae* | *Unclassified* | Wenzhou sobemo-like virus 3 | 17CxNGK-Ctr148 | BBQ04491 |
|  | *Solemoviridae* | *Unclassified* | Pyongtaek Culex sobemo-like virus | A18.2268 | UGO57111 |
|  | *Solemoviridae* | *Unclassified* | Culex-associated Luteo-like virus | CMS002_041a_COAV | QRW42557 |
|  | *Solemoviridae* | *Unclassified* | Hubei sobemo-like virus 13 | WHYY18840 | YP_009330098 |
|  | *Solemoviridae* | *Unclassified* | Jeffords solemo-like virus | GudgUn_DN37174-10 | QIJ70118 |
|  | *Solemoviridae* | *Unclassified* | Teise virus | UK1 | AWA82273 |
|  | *Solemoviridae* | *Unclassified* | Motts Mill virus | MEL216M | AWY11139 |
|  | *Solemoviridae* | *Solemoviridae* | Beet mild yellowing virus | DSMZ PV-1210 | QYA72324 |
|  | *Solemoviridae* | *Solemoviridae* | Groundnut rosette assistor virus | SC7.2 | QGY99249 |
|  | *Solemoviridae* | *Solemoviridae* | Rice yellow mottle virus | Ng18 | CBA11880 |
|  | *Solemoviridae* | *Solemoviridae* | Turnip rosette virus | - | AAO24320 |
|  | *Solemoviridae* | *Unclassified* | Norway luteo-like virus 3 | RTS-109 | AUX13126 |
|  | *Solemoviridae* | *Unclassified* | Ixodes scapularis associated virus 1 | ISE6 | BBD75429 |
|  | *Solemoviridae* | *Unclassified* | American dog tick associated virus-1 | NOR/A2/Bronnoya/2014 | ASY03257 |
|  | *Solemoviridae* | *Unclassified* | Xinjiang tick associated virus 1 | 14-YG | MH688544 |
|  | *Solemoviridae* | *Unclassified* | Xinjiang tick associated virus 1 | 16-T2 | MH688545 |
|  | *Solemoviridae* | *Unclassified* | Jilin luteo-like virus 2 | JL-QG-3 | MT316413 |
|  | *Solemoviridae* | *Unclassified* | Jilin luteo-like virus 3 | JL-QG-2 | MT316412 |
|  | *Solemoviridae* | *Unclassified* | Norway luteo-like virus 2 | NOR/A2/Bronnoya/2014 | MF141068 |
|  | *Solemoviridae* | *Unclassified* | Norway luteo-like virus 2 | NOR/H3/Skanevik/2014 | MF141067 |
| *Unclassified* | | | | | |
|  | *Unclassified* | *Unclassified* | Ixodes scapularis associated virus 3 | ISE6 | LC094965 |
|  | *Unclassified* | *Unclassified* | Ixodes scapularis associated virus 4 | RTS-11 | MF962656 |
|  | *Unclassified* | *Unclassified* | American dog tick associated virus 2 | RTS-100 | MF962662 |
|  | *Unclassified* | *Unclassified* | American dog tick associated virus 3 | RTS-1100 | MF962657 |
|  | *Unclassified* | *Unclassified* | Xinjiang tick associated virus 2 | 15-CYFC39 | MH688546 |
|  | *Unclassified* | *Unclassified* | Xinjiang tick associated virus 2 | 381 unRc | MZ244342 |
