# Supplementary material for: Extensive diversity of RNA viruses in ticks revealed by metagenomics in northeastern China
Source: PLoS Negl Trop Dis. 2022 Dec 21;16(12):e0011017. doi: 10.1371/journal.pntd.0011017 (PMC9836300; doi:10.1371/journal.pntd.0011017)
Supplement: S3 Table — (DOCX) [file pntd.0011017.s003.docx]

S3 Table. Nucleotide sequences of the identified viruses in the present study^*^

| Viral species | Segments/Gene | Collection sites | Host species | Viral strain | GenBank accession NO. |
| --- | --- | --- | --- | --- | --- |
| Alongshan virus | 1 | China: Tahe, Heilongjiang | *Ixodes persulcatus* | NE-TH4 | ON408067 |
| Alongshan virus | 2 | China: Tahe, Heilongjiang | *Ixodes persulcatus* | NE-TH4 | ON408068 |
| Alongshan virus | 3 | China: Tahe, Heilongjiang | *Ixodes persulcatus* | NE-TH4 | ON408069 |
| Alongshan virus | 4 | China: Tahe, Heilongjiang | *Ixodes persulcatus* | NE-TH4 | ON408070 |
| Tick-borne encephalitis virus | Complete genome | China: Tahe, Heilongjiang | *Ixodes persulcatus* | NE-TH3 | ON408071 |
| Tick-borne encephalitis virus | Complete genome | China: Tahe, Heilongjiang | *Ixodes persulcatus* | NE-TH4 | ON408072 |
| Tick-borne encephalitis virus | Complete genome | China: Songling, Inner Mongolia | *Ixodes persulcatus* | NE-SL4 | ON408073 |
| Bole tick virus 4 | Complete genome | China: Dunhua, Jilin | *Dermacentor silvarum* | NE-DH2 | ON408074 |
| Bole tick virus 4 | Complete genome | China: Shulan, Jilin | *Dermacentor silvarum* | NE-ShL2 | ON408075 |
| Songling virus | L | China: Tahe, Heilongjiang | *Haemaphysalis conicinna* | NE-TH1 | ON408076 |
| Songling virus | M | China: Tahe, Heilongjiang | *Haemaphysalis conicinna* | NE-TH1 | ON408077 |
| Songling virus | S | China: Tahe, Heilongjiang | *Haemaphysalis conicinna* | NE-TH1 | ON408078 |
| Songling virus | L | China: Tahe, Heilongjiang | *Haemaphysalis conicinna* | NE-TH2 | ON408079 |
| Songling virus | M | China: Tahe, Heilongjiang | *Haemaphysalis conicinna* | NE-TH2 | ON408080 |
| Songling virus | S | China: Tahe, Heilongjiang | *Haemaphysalis conicinna* | NE-TH2 | ON408081 |
| Ji’an nariovirus | L | China: Mudanjiang, Heilongjiang | *Haemaphysalis conicinna* | NE-MDJ1 | ON408082 |
| Ji’an nariovirus | M | China: Mudanjiang, Heilongjiang | *Haemaphysalis conicinna* | NE-MDJ1 | ON408083 |
| Ji’an nariovirus | S | China: Mudanjiang, Heilongjiang | *Haemaphysalis conicinna* | NE-MDJ1 | ON408084 |
| Ji’an nariovirus | L | China: Yichun, Heilongjiang | *Haemaphysalis conicinna* | NE-YC2 | ON408085 |
| Ji’an nariovirus | M | China: Yichun, Heilongjiang | *Haemaphysalis conicinna* | NE-YC2 | ON408086 |
| Ji’an nariovirus | S | China: Yichun, Heilongjiang | *Haemaphysalis conicinna* | NE-YC2 | ON408087 |
| Ji’an nariovirus | L | China: Ji'an, Jilin | *Haemaphysalis japonica* | NE-JA | ON408088 |
| Ji’an nariovirus | M | China: Ji'an, Jilin | *Haemaphysalis japonica* | NE-JA | ON408089 |
| Ji’an nariovirus | S | China: Ji'an, Jilin | *Haemaphysalis japonica* | NE-JA | ON408090 |
| Ji’an nariovirus | L | China: Dunhua, Jilin | *Haemaphysalis japonica* | NE-DH1 | ON408091 |
| Ji’an nariovirus | M | China: Dunhua, Jilin | *Haemaphysalis japonica* | NE-DH1 | ON408092 |
| Ji’an nariovirus | S | China: Dunhua, Jilin | *Haemaphysalis japonica* | NE-DH1 | ON408093 |
| Beiji nariovirus | L | China: Songling, Heilongjiang | *Ixodes persulcatus* | NE-SL4 | ON408094 |
| Beiji nariovirus | S | China: Songling, Heilongjiang | *Ixodes persulcatus* | NE-SL4 | ON408095 |
| Beiji nariovirus | L | China: Songling, Heilongjiang | *Ixodes persulcatus* | NE-SL3 | ON408096 |
| Beiji nariovirus | S | China: Songling, Heilongjiang | *Ixodes persulcatus* | NE-SL3 | ON408097 |
| Beiji nariovirus | L | China: Tahe, Heilongjiang | *Ixodes persulcatus* | NE-TH3 | ON408098 |
| Beiji nariovirus | S | China: Tahe, Heilongjiang | *Ixodes persulcatus* | NE-TH3 | ON408099 |
| Beiji nariovirus | L | China: Tahe, Heilongjiang | *Ixodes persulcatus* | NE-TH4 | ON408100 |
| Beiji nariovirus | S | China: Tahe, Heilongjiang | *Ixodes persulcatus* | NE-TH4 | ON408101 |
| Beiji nariovirus | L | China: Yichun, Heilongjiang | *Ixodes persulcatus* | NE-YC4 | ON408102 |
| Beiji nariovirus | S | China: Yichun, Heilongjiang | *Ixodes persulcatus* | NE-YC4 | ON408103 |
| Beiji nariovirus | L | China: Yichun, Heilongjiang | *Ixodes persulcatus* | NE-YC3 | ON408104 |
| Beiji nariovirus | S | China: Yichun, Heilongjiang | *Ixodes persulcatus* | NE-YC3 | ON408105 |
| Beiji nariovirus | L | China: Dunhua, Jilin | *Ixodes persulcatus* | NE-DH3 | ON408106 |
| Beiji nariovirus | S | China: Dunhua, Jilin | *Ixodes persulcatus* | NE-DH3 | ON408107 |
| Yichun nariovirus | L | China: Yichun, Heilongjiang | *Ixodes persulcatus* | NE-YC4 | ON408108 |
| Yichun nariovirus | S | China: Yichun, Heilongjiang | *Ixodes persulcatus* | NE-YC4 | ON408109 |
| Yichun nariovirus | L | China: Yichun, Heilongjiang | *Ixodes persulcatus* | NE-YC3 | ON408110 |
| Yichun nariovirus | S | China: Yichun, Heilongjiang | *Ixodes persulcatus* | NE-YC3 | ON408111 |
| Yichun nariovirus | L | China: Fangzheng, Heilongjiang | *Ixodes persulcatus* | NE-FZ3 | ON408112 |
| Yichun nariovirus | S | China: Fangzheng, Heilongjiang | *Ixodes persulcatus* | NE-FZ3 | ON408113 |
| Mukawa virus | L | China: Tahe, Heilongjiang | *Ixodes persulcatus* | NE-TH3 | ON408114 |
| Mukawa virus | M | China: Tahe, Heilongjiang | *Ixodes persulcatus* | NE-TH3 | ON408115 |
| Mukawa virus | S | China: Tahe, Heilongjiang | *Ixodes persulcatus* | NE-TH3 | ON408116 |
| Mukawa virus | L | China: Yichun, Heilongjiang | *Ixodes persulcatus* | NE-YC4 | ON408117 |
| Mukawa virus | M | China: Yichun, Heilongjiang | *Ixodes persulcatus* | NE-YC4 | ON408118 |
| Mukawa virus | S | China: Yichun, Heilongjiang | *Ixodes persulcatus* | NE-YC4 | ON408119 |
| Mukawa virus | L | China: Fangzheng, Heilongjiang | *Ixodes persulcatus* | NE-FZ2 | ON408120 |
| Mukawa virus | M | China: Fangzheng, Heilongjiang | *Ixodes persulcatus* | NE-FZ2 | ON408121 |
| Mukawa virus | S | China: Fangzheng, Heilongjiang | *Ixodes persulcatus* | NE-FZ2 | ON408122 |
| Mukawa virus | L | China: Fangzheng, Heilongjiang | *Ixodes persulcatus* | NE-FZ3 | ON408123 |
| Mukawa virus | M | China: Fangzheng, Heilongjiang | *Ixodes persulcatus* | NE-FZ3 | ON408124 |
| Mukawa virus | S | China: Fangzheng, Heilongjiang | *Ixodes persulcatus* | NE-FZ3 | ON408125 |
| Mukawa virus | L | China: Shulan, Jilin | *Dermacentor silvarum* | NE-ShL2 | ON408126 |
| Mukawa virus | M | China: Shulan, Jilin | *Dermacentor silvarum* | NE-ShL2 | ON408127 |
| Mukawa virus | S | China: Shulan, Jilin | *Dermacentor silvarum* | NE-ShL2 | ON408128 |
| Mukawa virus | L | China: Dunhua, Jilin | *Ixodes persulcatus* | NE-DH3 | ON408129 |
| Mukawa virus | M | China: Dunhua, Jilin | *Ixodes persulcatus* | NE-DH3 | ON408130 |
| Mukawa virus | S | China: Dunhua, Jilin | *Ixodes persulcatus* | NE-DH3 | ON408131 |
| Mudanjiang phlebovirus | L | China: Fangzheng, Heilongjiang | *Ixodes persulcatus* | NE-FZ3 | ON408132 |
| Mudanjiang phlebovirus | M | China: Fangzheng, Heilongjiang | *Ixodes persulcatus* | NE-FZ3 | ON408133 |
| Mudanjiang phlebovirus | S | China: Fangzheng, Heilongjiang | *Ixodes persulcatus* | NE-FZ3 | ON408134 |
| Mudanjiang phlebovirus | L | China: Mudanjiang, Heilongjiang | *Ixodes persulcatus* | NE-MDJ2 | ON408135 |
| Mudanjiang phlebovirus | M | China: Mudanjiang, Heilongjiang | *Ixodes persulcatus* | NE-MDJ2 | ON408136 |
| Mudanjiang phlebovirus | S | China: Mudanjiang, Heilongjiang | *Ixodes persulcatus* | NE-MDJ2 | ON408137 |
| Sara tick phlebovirus | L | China: Tahe, Heilongjiang | *Ixodes persulcatus* | NE-TH3 | ON408138 |
| Sara tick phlebovirus | S | China: Tahe, Heilongjiang | *Ixodes persulcatus* | NE-TH3 | ON408139 |
| Sara tick phlebovirus | L | China: Tahe, Heilongjiang | *Ixodes persulcatus* | NE-TH4 | ON408140 |
| Sara tick phlebovirus | S | China: Tahe, Heilongjiang | *Ixodes persulcatus* | NE-TH4 | ON408141 |
| Sara tick phlebovirus | L | China: Songling, Heilongjiang | *Ixodes persulcatus* | NE-SL3 | ON408142 |
| Sara tick phlebovirus | S | China: Songling, Heilongjiang | *Ixodes persulcatus* | NE-SL3 | ON408143 |
| Sara tick phlebovirus | L | China: Songling, Heilongjiang | *Ixodes persulcatus* | NE-SL4 | ON408144 |
| Sara tick phlebovirus | S | China: Songling, Heilongjiang | *Ixodes persulcatus* | NE-SL4 | ON408145 |
| Sara tick phlebovirus | L | China: Yichun, Heilongjiang | *Ixodes persulcatus* | NE-YC3 | ON408146 |
| Sara tick phlebovirus | S | China: Yichun, Heilongjiang | *Ixodes persulcatus* | NE-YC3 | ON408147 |
| Sara tick phlebovirus | L | China: Yichun, Heilongjiang | *Ixodes persulcatus* | NE-YC4 | ON408148 |
| Sara tick phlebovirus | S | China: Yichun, Heilongjiang | *Ixodes persulcatus* | NE-YC4 | ON408149 |
| Onega tick phlebovirus | L | China: Tahe, Heilongjiang | *Ixodes persulcatus* | NE-TH3 | ON408150 |
| Onega tick phlebovirus | S | China: Tahe, Heilongjiang | *Ixodes persulcatus* | NE-TH3 | ON408151 |
| Onega tick phlebovirus | L | China: Tahe, Heilongjiang | *Ixodes persulcatus* | NE-TH4 | ON408152 |
| Onega tick phlebovirus | S | China: Tahe, Heilongjiang | *Ixodes persulcatus* | NE-TH4 | ON408153 |
| Onega tick phlebovirus | L | China: Songling, Heilongjiang | *Ixodes persulcatus* | NE-SL3 | ON408154 |
| Onega tick phlebovirus | S | China: Songling, Heilongjiang | *Ixodes persulcatus* | NE-SL3 | ON408155 |
| Onega tick phlebovirus | L | China: Songling, Heilongjiang | *Ixodes persulcatus* | NE-SL4 | ON408156 |
| Onega tick phlebovirus | S | China: Songling, Heilongjiang | *Ixodes persulcatus* | NE-SL4 | ON408157 |
| Onega tick phlebovirus | L | China: Yichun, Heilongjiang | *Ixodes persulcatus* | NE-YC3 | ON408158 |
| Onega tick phlebovirus | S | China: Yichun, Heilongjiang | *Ixodes persulcatus* | NE-YC3 | ON408159 |
| Onega tick phlebovirus | L | China: Yichun, Heilongjiang | *Ixodes persulcatus* | NE-YC4 | ON408160 |
| Onega tick phlebovirus | S | China: Yichun, Heilongjiang | *Ixodes persulcatus* | NE-YC4 | ON408161 |
| Tahe rhabdovirus 1 | Complete genome | China: Tahe, Heilongjiang | *Haemaphysalis conicinna* | NE-TH1 | ON408162 |
| Tahe rhabdovirus 1 | Complete genome | China: Tahe, Heilongjiang | *Haemaphysalis conicinna* | NE-TH2 | ON408163 |
| Tahe rhabdovirus 1 | Complete genome | China: Songling, Heilongjiang | *Haemaphysalis conicinna* | NE-SL1 | ON408164 |
| Tahe rhabdovirus 1 | Complete genome | China: Songling, Heilongjiang | *Haemaphysalis conicinna* | NE-SL2 | ON408165 |
| Tahe rhabdovirus 1 | Complete genome | China: Dunhua, Jilin | *Haemaphysalis japonica* | NE-DH1 | ON408166 |
| Tahe rhabdovirus 1 | Complete genome | China: Yichun, Heilongjiang | *Haemaphysalis conicinna* | NE-YC2 | ON408167 |
| Tahe rhabdovirus 1 | Complete genome | China: Shulan, Jilin | *Dermacentor silvarum* | NE-ShL3 | ON408168 |
| Tahe rhabdovirus 1 | Complete genome | China: Ji'an, Jilin | *Haemaphysalis japonica* | NE-JA | ON408169 |
| Tahe rhabdovirus 2 | Complete genome | China: Tahe, Heilongjiang | *Ixodes persulcatus* | NE-TH3 | ON408170 |
| Tahe rhabdovirus 2 | Complete genome | China: Tahe, Heilongjiang | *Ixodes persulcatus* | NE-TH4 | ON408171 |
| Tahe rhabdovirus 3 | Complete genome | China: Tahe, Heilongjiang | *Ixodes persulcatus* | NE-TH3 | ON408172 |
| Nuomin virus | Complete genome | China: Mudanjiang, Heilongjiang | *Ixodes persulcatus* | MDJ2 | ON408173 |
| Nuomin virus | Complete genome | China: Fangzheng, Heilongjiang | *Ixodes persulcatus* | FZ2 | ON408174 |
| Nuomin virus | Complete genome | China: Fangzheng, Heilongjiang | *Ixodes persulcatus* | FZ3 | ON408175 |
| Nuomin virus | Complete genome | China: Yichun, Heilongjiang | *Ixodes persulcatus* | YC4 | ON408176 |
| Nuomin virus | Complete genome | China: Yichun, Heilongjiang | *Ixodes persulcatus* | YC3 | ON408177 |
| Nuomin virus | Complete genome | China: Songling, Heilongjiang | *Ixodes persulcatus* | SL3 | ON408178 |
| Nuomin virus | Complete genome | China: Songling, Heilongjiang | *Ixodes persulcatus* | SL4 | ON408179 |
| Nuomin virus | Complete genome | China: Tahe, Heilongjiang | *Ixodes persulcatus* | TH3 | ON408180 |
| Nuomin virus | Complete genome | China: Tahe, Heilongjiang | *Ixodes persulcatus* | TH4 | ON408181 |
| Nuomin virus | Complete genome | China: Dunhua, Jilin | *Ixodes persulcatus* | DH3 | ON408182 |
| Yichun mivirus | Complete genome | China: Yichun, Heilongjiang | *Ixodes persulcatus* | YC4 | ON408183 |
| Yichun mivirus | Complete genome | China: Dunhua, Jilin | *Ixodes persulcatus* | DH3 | ON408184 |
| Jilin partiti-like virus 1 | Complete cds | China: Yichun, Heilongjiang | *Ixodes persulcatus* | YC3 | ON408185 |
| Jilin partiti-like virus 1 | Complete cds | China: Yichun, Heilongjiang | *Ixodes persulcatus* | YC4 | ON408186 |
| Jilin partiti-like virus 1 | Complete cds | China: Songling, Heilongjiang | *Ixodes persulcatus* | SL3 | ON408187 |
| Jilin partiti-like virus 1 | Complete cds | China: Dunhua, Jilin | *Ixodes persulcatus* | DH3 | ON408188 |
| Jilin partiti-like virus 1 | Complete cds | China: Mudanjiang, Heilongjiang | *Ixodes persulcatus* | MDJ2 | ON408189 |
| Jilin partiti-like virus 1 | Complete cds | China: Fangzheng, Heilongjiang | *Ixodes persulcatus* | FZ3 | ON408190 |
| Jilin partiti-like virus 1 | Complete cds | China: Fangzheng, Heilongjiang | *Ixodes persulcatus* | FZ2 | ON408191 |
| Fangzheng tombus-like virus | Complete cds | China: Yichun, Heilongjiang | *Ixodes persulcatus* | YC3 | ON408192 |
| Fangzheng tombus-like virus | Complete cds | China: Fangzheng, Heilongjiang | *Ixodes persulcatus* | FZ3 | ON408193 |
| Fangzheng tombus-like virus | Complete cds | China: Fangzheng, Heilongjiang | *Ixodes persulcatus* | FZ2 | ON408194 |
| Fangzheng tombus-like virus | Complete cds | China: Dunhua, Jilin | *Ixodes persulcatus* | DH3 | ON408195 |
| Ixodes scapularis associated virus 1 | Complete cds | China: Yichun, Heilongjiang | *Ixodes persulcatus* | TH4 | ON408196 |
| Ixodes scapularis associated virus 1 | Complete cds | China: Songling, Heilongjiang | *Ixodes persulcatus* | SL3 | ON408197 |
| Ixodes scapularis associated virus 1 | Complete cds | China: Songling, Heilongjiang | *Ixodes persulcatus* | SL4 | ON408198 |
| Ixodes scapularis associated virus 1 | Complete cds | China: Yichun, Heilongjiang | *Ixodes persulcatus* | YC3 | ON408199 |
| Ixodes scapularis associated virus 1 | Complete cds | China: Yichun, Heilongjiang | *Ixodes persulcatus* | YC4 | ON408200 |
| Ixodes scapularis associated virus 1 | Complete cds | China: Dunhua, Jilin | *Ixodes persulcatus* | DH3 | ON408201 |
| Xinjiang tick associated virus 1 | Complete cds | China: Dunhua, Jilin | *Dermacentor silvarum* | DH2 | ON408202 |
| Xinjiang tick associated virus 1 | Complete cds | China: Shulan, Jilin | *Dermacentor silvarum* | ShL1 | ON408203 |
| Xinjiang tick associated virus 1 | Complete cds | China: Shulan, Jilin | *Dermacentor silvarum* | ShL2 | ON408204 |
| Xinjiang tick associated virus 1 | Complete cds | China: Shulan, Jilin | *Dermacentor silvarum* | ShL3 | ON408205 |
| Jilin luteo-like virus 2 | Complete cds | China: Tahe, Heilongjiang | *Ixodes persulcatus* | TH3 | ON408206 |
| Jilin luteo-like virus 2 | Complete cds | China: Songling, Heilongjiang | *Ixodes persulcatus* | SL4 | ON408207 |
| Jilin luteo-like virus 2 | Complete cds | China: Yichun, Heilongjiang | *Ixodes persulcatus* | YC3 | ON408208 |
| Jilin luteo-like virus 2 | Complete cds | China: Yichun, Heilongjiang | *Ixodes persulcatus* | YC4 | ON408209 |
| Jilin luteo-like virus 2 | Complete cds | China: Fangzheng, Heilongjiang | *Ixodes persulcatus* | FZ3 | ON408210 |
| Jilin luteo-like virus 2 | Complete cds | China: Dunhua, Jilin | *Ixodes persulcatus* | DH3 | ON408211 |
| *Ixodes scapularis* associated virus 3 | Complete cds | China: Tahe, Heilongjiang | *Ixodes persulcatus* | TH4 | ON408212 |
| *Ixodes scapularis* associated virus 3 | Complete cds | China: Songling, Heilongjiang | *Ixodes persulcatus* | SL3 | ON408213 |
| *Ixodes scapularis* associated virus 3 | Complete cds | China: Songling, Heilongjiang | *Ixodes persulcatus* | SL4 | ON408214 |
| *Ixodes scapularis* associated virus 3 | Complete cds | China: Yichun, Heilongjiang | *Ixodes persulcatus* | YC3 | ON408215 |
| *Ixodes scapularis* associated virus 3 | Complete cds | China: Yichun, Heilongjiang | *Ixodes persulcatus* | YC4 | ON408216 |

^*^cds, coding sequence.
