# Supplementary material for: Extensive diversity of RNA viruses in ticks revealed by metagenomics in northeastern China
Source: PLoS Negl Trop Dis. 2022 Dec 21;16(12):e0011017. doi: 10.1371/journal.pntd.0011017 (PMC9836300; doi:10.1371/journal.pntd.0011017)
Supplement: S4 Table — (DOCX) [file pntd.0011017.s004.docx]

S4 Table. Nucleotide sequence similarity of S1 (upper right) and S2 (lower left) segments of ALSV^*^

|  | ALSV TH4 | ALSV H3 | ALSV HLJ1 | ALSV HLJ2 | ALSV Miass527 | ALSV Miass502 | ALSV Miass519 | ALSV Miass506 | ALSV Kuutsalo-23 | ALSV Haapasaari-18 | TKCV IM-OI70 | XJTV1 XJO381 | YGTV XJ-YGTV-1 | HLJTV HLJ41 | JMTV HLJ41 | GXTV GX46 |
| --- | --- | --- | --- | --- | --- | --- | --- | --- | --- | --- | --- | --- | --- | --- | --- | --- |
| ALSV TH4 | *** | 97 | 95.8 | 95.8 | 89.6 | 89.4 | 88.9 | 88.9 | 89.2 | 89 | 73.6 | 72.8 | 72.1 | 71.8 | 71.9 | 71.8 |
| ALSV H3 | 98.2 | *** | 95.5 | 95.5 | 89.6 | 89.4 | 89 | 89 | 89.6 | 89.5 | 74.2 | 73.1 | 72.3 | 72.2 | 72 | 72.2 |
| ALSV HLJ1 | 94.2 | 94.8 | *** | 100 | 89.3 | 89.2 | 88 | 88 | 89.4 | 89.2 | 73.5 | 72.9 | 71.7 | 71.2 | 71.2 | 71.2 |
| ALSV HLJ2 | 94.2 | 94.8 | 100 | *** | 89.3 | 89.2 | 88 | 88 | 89.4 | 89.2 | 73.5 | 72.9 | 71.7 | 71.2 | 71.2 | 71.2 |
| ALSV Miass527 | 93.9 | 94.2 | 96.7 | 96.7 | *** | 99.5 | 90.7 | 90.7 | 89.4 | 88.8 | 73.8 | 72.2 | 71.8 | 71.1 | 71 | 71.1 |
| ALSV Miass502 | 93.8 | 94.1 | 96.5 | 96.5 | 99.5 | *** | 90.5 | 90.5 | 89.2 | 88.7 | 73.6 | 72.1 | 71.8 | 71 | 70.9 | 71 |
| ALSV Miass519 | 92.1 | 91.8 | 92.2 | 92.2 | 92.4 | 92.3 | *** | 100 | 89.1 | 88.8 | 73.8 | 72.3 | 71.6 | 71.9 | 71.6 | 71.9 |
| ALSV Miass506 | 92.1 | 91.8 | 92.2 | 92.2 | 92.4 | 92.3 | 100 | *** | 89.1 | 88.8 | 73.8 | 72.3 | 71.6 | 71.9 | 71.6 | 71.9 |
| ALSV Kuutsalo-23 | 91.3 | 91.1 | 91.2 | 91.2 | 92 | 91.9 | 93.6 | 93.6 | *** | 95.8 | 74.2 | 71.8 | 72.1 | 71.3 | 71.1 | 71.3 |
| ALSV Haapasaari-18 | 91.3 | 91 | 90.8 | 90.8 | 91.7 | 91.6 | 93.9 | 93.9 | 98.4 | *** | 74.1 | 71.3 | 72 | 71.7 | 71.1 | 71.7 |
| TKCV IM-OI70 | 69.1 | 68.8 | 68.9 | 68.9 | 69.1 | 69.1 | 67.4 | 67.4 | 68.5 | 68.4 | *** | 72.2 | 72.1 | 71.6 | 71.1 | 71.6 |
| XJTV1 XJO381 | 64.9 | 64.8 | 64.9 | 64.9 | 65.7 | 65.7 | 65.1 | 65.1 | 65.1 | 65 | 62.6 | *** | 77.2 | 70.3 | 69.8 | 70.3 |
| YGTV XJ-YGTV-1 | 64.6 | 64.5 | 65.2 | 65.2 | 65.6 | 65.4 | 65.6 | 65.6 | 66 | 66 | 64.6 | 76.2 | *** | 69.7 | 71 | 69.7 |
| HLJTV HLJ41 | 59.7 | 59.6 | 60.4 | 60.4 | 59.8 | 59.6 | 59.2 | 59.2 | 59.8 | 59.7 | 58.7 | 58.6 | 58.6 | *** | 93 | 100 |
| JMTV HLJ41 | 58.8 | 58.7 | 59.8 | 59.8 | 58.8 | 58.8 | 58.7 | 58.7 | 59.6 | 59.6 | 59 | 57.8 | 57.5 | 94.3 | *** | 93 |
| GXTV GX46 | 59.7 | 59.6 | 60.4 | 60.4 | 59.8 | 59.6 | 59.2 | 59.2 | 59.8 | 59.7 | 58.7 | 58.6 | 58.6 | 100 | 94.3 | *** |

^*^Abbreviations: ALSV, Alongshan virus; TKCV, Takachi virus; XJTV, Xinjiang tick virus; YGTV, Yanggou tick virus; HLJTV, Heilongjiang tick virus; JMTV, Jingmen tick virus; GXTV, Guangxi tick virus.
