# Supplementary material for: Extensive diversity of RNA viruses in ticks revealed by metagenomics in northeastern China
Source: PLoS Negl Trop Dis. 2022 Dec 21;16(12):e0011017. doi: 10.1371/journal.pntd.0011017 (PMC9836300; doi:10.1371/journal.pntd.0011017)
Supplement: S5 Table — (DOCX) [file pntd.0011017.s005.docx]

S5 Table. Nucleotide sequence similarity of S3 (upper right) and S4 (lower left) segments of ALSV^*^

|  | ALSV TH4 | ALSV H3 | ALSV HLJ1 | ALSV HLJ2 | ALSV Miass527 | ALSV Miass502 | ALSV Miass519 | ALSV Miass506 | ALSV Kuutsalo-23 | ALSV Haapasaari-18 | TKCV IM-OI70 | XJTV1 XJO381 | YGTV XJ-YGTV-1 | HLJTV HLJ41 | JMTV HLJ41 | GXTV GX46 |
| --- | --- | --- | --- | --- | --- | --- | --- | --- | --- | --- | --- | --- | --- | --- | --- | --- |
| ALSV TH4 | *** | 97.8 | 93.2 | 93.2 | 91 | 91.1 | 90.9 | 90.9 | 90 | 90.5 | 75.6 | 73.2 | 72.8 | 72.3 | 72 | 72.3 |
| ALSV H3 | 98.5 | *** | 93.1 | 93.1 | 90.1 | 90.2 | 90.5 | 90.5 | 90.3 | 90.3 | 75.4 | 73.4 | 72.9 | 72.2 | 72.2 | 72.2 |
| ALSV HLJ1 | 97.3 | 97 | *** | 100 | 90.3 | 90.5 | 91.1 | 91.1 | 90.2 | 90.3 | 76 | 73.4 | 73.5 | 72.2 | 72.1 | 72.2 |
| ALSV HLJ2 | 97.3 | 97 | 100 | *** | 90.3 | 90.5 | 91.1 | 91.1 | 90.2 | 90.3 | 76 | 73.4 | 73.5 | 72.2 | 72.1 | 72.2 |
| ALSV Miass527 | 89.6 | 89.6 | 89.5 | 89.5 | *** | 99.8 | 96.7 | 96.7 | 90.1 | 89.3 | 76 | 73.6 | 73.9 | 72.1 | 72 | 72.1 |
| ALSV Miass502 | 89.4 | 89.4 | 89.2 | 89.2 | 99.5 | *** | 96.9 | 96.9 | 90.1 | 89.4 | 76 | 73.5 | 74 | 72.1 | 72 | 72.1 |
| ALSV Miass519 | 89.5 | 89.5 | 89.1 | 89.1 | 98.5 | 98.1 | *** | 100 | 90.3 | 89.4 | 76 | 73.2 | 73.7 | 72.4 | 72.4 | 72.4 |
| ALSV Miass506 | 89.5 | 89.5 | 89.1 | 89.1 | 98.5 | 98.2 | 100 | *** | 90.3 | 89.4 | 76 | 73.2 | 73.7 | 72.4 | 72.4 | 72.4 |
| ALSV Kuutsalo-23 | 90.1 | 90.1 | 89.6 | 89.6 | 90.1 | 89.8 | 90.1 | 90.1 | *** | 94.4 | 75.6 | 72.8 | 73.2 | 71.2 | 70.9 | 71.2 |
| ALSV Haapasaari-18 | 89.6 | 89.7 | 89 | 89 | 90.2 | 89.9 | 90 | 90 | 94 | *** | 75.8 | 72.6 | 73.7 | 71.9 | 71.6 | 71.9 |
| TKCV IM-OI70 | 72.8 | 73 | 72.9 | 72.9 | 72.6 | 72.3 | 73 | 73 | 72.6 | 72.8 | *** | 73 | 73.8 | 71.7 | 70.9 | 71.7 |
| XJTV1 XJO381 | 69.3 | 69.4 | 68.8 | 68.8 | 69.3 | 68.9 | 69.3 | 69.2 | 70 | 68.8 | 68.4 | *** | 77.6 | 70.8 | 70.6 | 70.8 |
| YGTV XJ-YGTV-1 | 68.4 | 68.3 | 68.5 | 68.5 | 68.1 | 67.7 | 68.1 | 68 | 68.6 | 68.1 | 68.2 | 76.1 | *** | 70.2 | 69.7 | 70.2 |
| HLJTV HLJ41 | 64.9 | 64.9 | 64.8 | 64.8 | 65.4 | 65.1 | 65 | 64.9 | 65.1 | 65.3 | 65.9 | 63.1 | 62.2 | *** | 94.7 | 100 |
| JMTV HLJ41 | 64.5 | 64.5 | 64.4 | 64.4 | 65.2 | 64.9 | 65.1 | 65.1 | 65.5 | 65.4 | 65.2 | 62.8 | 62 | 92.7 | *** | 94.7 |
| GXTV GX46 | 64.9 | 64.9 | 64.8 | 64.8 | 65.4 | 65.1 | 65 | 64.9 | 65.1 | 65.3 | 65.9 | 63.1 | 62.2 | 100 | 92.7 | *** |

^*^Abbreviations: ALSV, Alongshan virus; TKCV, Takachi virus; XJTV, Xinjiang tick virus; YGTV, Yanggou tick virus; HLJTV, Heilongjiang tick virus; JMTV, Jingmen tick virus; GXTV, Guangxi tick virus.
