# Supplementary material for: Extensive diversity of RNA viruses in ticks revealed by metagenomics in northeastern China
Source: PLoS Negl Trop Dis. 2022 Dec 21;16(12):e0011017. doi: 10.1371/journal.pntd.0011017 (PMC9836300; doi:10.1371/journal.pntd.0011017)
Supplement: S6 Table — (DOCX) [file pntd.0011017.s006.docx]

S6 Table. Nucleotide sequence similarity of TBEVs^*^

|  | NE-TH3 | NE-TH4 | SL-TH4 | DXAL_T83 | HLB-T74 | JL_Jiaohe | JL-T75 | Senzhang | JLCB11-08 | JLCB11-35 | JLCB11-40 | MDJ01 | MDJ-03 | MDJ-02 |
| --- | --- | --- | --- | --- | --- | --- | --- | --- | --- | --- | --- | --- | --- | --- |
| NE-TH3 | *** | 93.5 | 97.7 | 93.5 | 93.5 | 94.3 | 93.8 | 93.5 | 93.9 | 93.9 | 93.9 | 93.5 | 93.6 | 93.6 |
| NE-TH4 | *** | *** | 93.9 | 98 | 98 | 93.7 | 95 | 94.8 | 95 | 95 | 95 | 95 | 94.8 | 94.8 |
| SL-TH4 | *** | *** | *** | 93.8 | 93.8 | 94.6 | 94.2 | 93.9 | 94.1 | 94.2 | 94.2 | 93.9 | 94 | 94 |
| DXAL_T83 | *** | *** | *** | *** | 99.7 | 93.5 | 94.7 | 94.4 | 94.7 | 94.7 | 94.7 | 94.6 | 94.5 | 94.5 |
| HLB-T74 | *** | *** | *** | *** | *** | 93.5 | 94.8 | 94.4 | 94.7 | 94.8 | 94.7 | 94.6 | 94.5 | 94.6 |
| JL_Jiaohe | *** | *** | *** | *** | *** | *** | 94.1 | 93.9 | 93.9 | 93.9 | 94 | 93.9 | 93.9 | 94 |
| JL-T75 | *** | *** | *** | *** | *** | *** | *** | 98.1 | 98.3 | 98.3 | 98.3 | 98.1 | 98.1 | 98.1 |
| Senzhang | *** | *** | *** | *** | *** | *** | *** | *** | 98.1 | 98.1 | 98.2 | 98 | 99.6 | 99.6 |
| JLCB11-08 | *** | *** | *** | *** | *** | *** | *** | *** | *** | 99.9 | 99.3 | 97.9 | 98.1 | 98.1 |
| JLCB11-35 | *** | *** | *** | *** | *** | *** | *** | *** | *** | *** | 99.3 | 98 | 98.1 | 98.1 |
| JLCB11-40 | *** | *** | *** | *** | *** | *** | *** | *** | *** | *** | *** | 98 | 98.2 | 98.2 |
| MDJ01 | *** | *** | *** | *** | *** | *** | *** | *** | *** | *** | *** | *** | 98.2 | 98.2 |
| MDJ-03 | *** | *** | *** | *** | *** | *** | *** | *** | *** | *** | *** | *** | *** | 99.9 |
| MDJ-02 | *** | *** | *** | *** | *** | *** | *** | *** | *** | *** | *** | *** | *** | *** |
