# Supplementary material for: Extensive diversity of RNA viruses in ticks revealed by metagenomics in northeastern China
Source: PLoS Negl Trop Dis. 2022 Dec 21;16(12):e0011017. doi: 10.1371/journal.pntd.0011017 (PMC9836300; doi:10.1371/journal.pntd.0011017)
Supplement: S7 Table — (DOCX) [file pntd.0011017.s007.docx]

S7 Table. Nucleotide sequence similarity of the complete genome (upper right) and amino acid sequence similarity of RdRp (lower left) of BLTV4

|  | ShL3 | DH2 | Iasi23 | Iasi21 | Iasi20 | Iasi50 | 17-L2 | bole4-xinjiang-JMN | GSC346flaviV | BLP-1 | Bangali/H.truncatum/2018 | Iftin/H.dromedarii/2018 | TTP-Pool-4 | Thailand tick flavivirus |
| --- | --- | --- | --- | --- | --- | --- | --- | --- | --- | --- | --- | --- | --- | --- |
| ShL3 | *** | 98.1 | 82.7 | 82.5 | 82.7 | 82.8 | 78.9 | 78.9 | 78.9 | 78.8 | 78.5 | 78.8 | 78 | 77.5 |
| DH2 | 98.5 | *** | 82.9 | 82.7 | 82.9 | 83 | 79 | 79.1 | 79.1 | 79 | 78.7 | 79 | 78.2 | 77.6 |
| Iasi23 | 95.4 | 95.4 | *** | 99.5 | 99.8 | 97.3 | 80.4 | 80.4 | 80.4 | 80.3 | 80.3 | 80.3 | 79.2 | 79.4 |
| Iasi21 | 95 | 95 | 99.6 | *** | 99.5 | 97 | 80.2 | 80.2 | 80.2 | 80.1 | 80.1 | 80.1 | 79 | 79.2 |
| Iasi20 | 95 | 95 | 99.6 | 99.2 | *** | 97.3 | 80.3 | 80.4 | 80.4 | 80.3 | 80.3 | 80.3 | 79.2 | 79.4 |
| Iasi50 | 94.6 | 94.6 | 99.2 | 99.6 | 98.8 | *** | 80.3 | 80.3 | 80.3 | 80.2 | 80.2 | 80.2 | 79.2 | 79.3 |
| 17-L2 | 95.4 | 95.8 | 93.8 | 93.5 | 94.2 | 93.1 | *** | 98.9 | 98.5 | 98.6 | 88.1 | 91.5 | 79.9 | 80.4 |
| bole4-xinjiang-JMN | 95.8 | 96.2 | 94.2 | 93.8 | 94.6 | 93.5 | 99.6 | *** | 98.5 | 98.8 | 88.1 | 91.5 | 80 | 80.5 |
| GSC346flaviV | 96.2 | 96.2 | 94.2 | 93.8 | 93.8 | 93.5 | 98.8 | 99.2 | *** | 98.2 | 88 | 91.3 | 79.9 | 80.4 |
| BLP-1 | 96.2 | 96.5 | 94.6 | 94.2 | 94.2 | 93.8 | 99.2 | 99.6 | 99.6 | *** | 88.1 | 91.4 | 79.9 | 80.4 |
| Bangali/H.truncatum/2018 | 96.2 | 96.2 | 95.4 | 95 | 95 | 94.6 | 96.9 | 97.3 | 97.3 | 97.7 | *** | 92.6 | 84.5 | 83.7 |
| Iftin/H.dromedarii/2018 | 96.9 | 96.9 | 96.2 | 95.8 | 95.8 | 95.4 | 97.7 | 98.1 | 98.1 | 98.5 | 99.2 | *** | 81.7 | 81.4 |
| TTP-Pool-4 | 95 | 95 | 95.4 | 95 | 95 | 94.6 | 95 | 95.4 | 95.4 | 95.8 | 96.5 | 97.3 | *** | 89.7 |
| Thailand tick flavivirus | 95.8 | 95.8 | 95 | 95.4 | 94.6 | 95 | 95.8 | 96.2 | 96.2 | 96.5 | 96.5 | 97.3 | 98.5 | *** |
