# Supplementary material for: Extensive diversity of RNA viruses in ticks revealed by metagenomics in northeastern China
Source: PLoS Negl Trop Dis. 2022 Dec 21;16(12):e0011017. doi: 10.1371/journal.pntd.0011017 (PMC9836300; doi:10.1371/journal.pntd.0011017)
Supplement: S8 Table — (DOCX) [file pntd.0011017.s008.docx]

S8 Table. Nucleotide sequence similarity of L segment (upper right) and amino acid sequence similarity of RdRp (lower left) of JANV and SGLV^*^

|  | JANV MDJ1 | JANV YC1 | JANV JA | JANV DH1 | SGLV TH3 | SGLV TH4 | SGLV YC585 | SGLV HLJ1202 | TCTV1 TC253 | SXTV2 SXO338nairoV | HNTV HNO321nairoV |
| --- | --- | --- | --- | --- | --- | --- | --- | --- | --- | --- | --- |
| JANV MDJ1 | *** | 99.2 | 99.1 | 98.4 | 73.3 | 73.4 | 73.4 | 73.1 | 61.6 | 63.2 | 64.6 |
| JANV YC1 | 99.7 | *** | 99.2 | 98.5 | 73.4 | 73.4 | 73.5 | 73.2 | 61.6 | 63.2 | 64.7 |
| JANV JA | 99.4 | 99.6 | *** | 98.7 | 73.3 | 73.4 | 73.4 | 73.1 | 61.5 | 63.2 | 64.6 |
| JANV DH1 | 99.6 | 99.7 | 99.6 | *** | 73.4 | 73.4 | 73.5 | 73.2 | 61.7 | 63.4 | 64.6 |
| SGLV TH3 | 85.1 | 85.2 | 85 | 85.1 | *** | 96.5 | 95.9 | 95.7 | 61.8 | 63.3 | 64 |
| SGLV TH4 | 85 | 85.1 | 84.9 | 85 | 99.4 | *** | 97 | 96.5 | 61.9 | 63.4 | 63.8 |
| SGLV YC585 | 84.8 | 84.9 | 84.7 | 84.8 | 99.1 | 99.2 | *** | 98.4 | 61.8 | 63.4 | 63.6 |
| SGLV HLJ1202 | 84.1 | 84.2 | 84 | 84.1 | 98.4 | 98.4 | 98.7 | *** | 61.7 | 63 | 63.4 |
| TCTV1 TC253 | 63.3 | 63.5 | 63.3 | 63.4 | 64 | 63.9 | 63.6 | 63.1 | *** | 62.3 | 61.7 |
| SXTV2 SXO338nairoV | 66 | 66.2 | 66 | 66.1 | 66.1 | 66.2 | 65.9 | 65.5 | 63.7 | *** | 66.4 |
| HNTV HNO321nairoV | 67.6 | 67.7 | 67.6 | 67.7 | 67.7 | 67.7 | 67.4 | 67 | 64.1 | 71.5 | *** |

^*^ Abbreviations: JANV, Ji’an nariovirus; SGLV, Songling virus; TCTV1, Tacheng tick virus 1; SXTV2, Shanxi tick virus 2; HNTV, Henan tick virus.
