# Supplementary material for: Extensive diversity of RNA viruses in ticks revealed by metagenomics in northeastern China
Source: PLoS Negl Trop Dis. 2022 Dec 21;16(12):e0011017. doi: 10.1371/journal.pntd.0011017 (PMC9836300; doi:10.1371/journal.pntd.0011017)
Supplement: S9 Table — (DOCX) [file pntd.0011017.s009.docx]

S9 Table. Nucleotide sequence similarity of M (upper right) and S (lower left) segments of JANV and SGLV^*^

|  | JANV MDJ1 | JANV YC1 | JANV JA | JANV DH1 | SGLV TH3 | SGLV TH4 | SGLV YC585 | SGLV HLJ1202 | TCTV1 TC253 | SXTV2 SXO338nairoV | HNTV HNO321nairoV |
| --- | --- | --- | --- | --- | --- | --- | --- | --- | --- | --- | --- |
| JANV MDJ1 | *** | 99.3 | 99.7 | 99.2 | 71.2 | 71.6 | 71.1 | 71.1 | 54.6 | 57.5 | 60.3 |
| JANV YC1 | 99.1 | *** | 99.3 | 99.8 | 71.1 | 71.5 | 71 | 70.9 | 54.7 | 57.5 | 60 |
| JANV JA | 99 | 98.8 | *** | 99.5 | 71.2 | 71.5 | 71.1 | 71 | 54.6 | 57.5 | 60.2 |
| JANV DH1 | 97.8 | 97.4 | 97.5 | *** | 71.1 | 71.4 | 71 | 70.9 | 54.7 | 57.4 | 60 |
| SGLV TH3 | 71.3 | 71.4 | 71.1 | 71.7 | *** | 92.4 | 98.1 | 97.6 | 55.4 | 57.3 | 61.4 |
| SGLV TH4 | 71.1 | 71.1 | 71.1 | 71.5 | 99.1 | *** | 92.3 | 92.1 | 54.9 | 57.3 | 61.8 |
| SGLV YC585 | 71.3 | 71.3 | 71.1 | 71.7 | 99.2 | 99.1 | *** | 98.9 | 55.3 | 57.4 | 61.6 |
| SGLV HLJ1202 | 70.9 | 70.9 | 70.7 | 71 | 98.5 | 98.4 | 99 | *** | 55.1 | 57.1 | 61.5 |
| TCTV1 TC253 | 59.7 | 59.7 | 59.9 | 60.4 | 59.2 | 59.2 | 59 | 58.7 | *** | 56.1 | 56.9 |
| SXTV2 SXO338nairoV | 61.5 | 61.5 | 61.5 | 61.5 | 62.1 | 62.2 | 61.9 | 61.4 | 58 | *** | 59.4 |
| HNTV HNO321nairoV | 61.6 | 61.3 | 61.5 | 61.7 | 62.3 | 62.5 | 62.3 | 61.7 | 60.9 | 66.7 | *** |

^*^ Abbreviations: JANV, Ji’an nariovirus; SGLV, Songling virus; TCTV1, Tacheng tick virus 1; SXTV2, Shanxi tick virus 2; HNTV, Henan tick virus.
