# Supplementary material for: Extensive diversity of RNA viruses in ticks revealed by metagenomics in northeastern China
Source: PLoS Negl Trop Dis. 2022 Dec 21;16(12):e0011017. doi: 10.1371/journal.pntd.0011017 (PMC9836300; doi:10.1371/journal.pntd.0011017)
Supplement: S10 Table — (DOCX) [file pntd.0011017.s010.docx]

S10 Table. Nucleotide sequence similarity of the L segment (upper right) and amino acid sequence similarity of RdRp (lower left) of YCNV and BJNV^*^

|  | YCNV YC4 | YCNV YC3 | YCNV FZ3 | BJNV SL4 | BJNV SL3 | BJNV TH3 | BJNV TH4 | BJNV YC4 | BJNV YC3 | BJNV DH3 | BJNV H1603 | BJNV H160 | BJNV H801 | BJNV H39 | BJNV H56 | BJNV H59 | GKTV | PTV | NWNV1 | GTV |
| --- | --- | --- | --- | --- | --- | --- | --- | --- | --- | --- | --- | --- | --- | --- | --- | --- | --- | --- | --- | --- |
| YCNV YC4 | *** | 98.6 | 98.7 | 83.1 | 83 | 83 | 83 | 83.1 | 83.1 | 83.1 | 82.9 | 82.9 | 82.9 | 82.9 | 82.5 | 82.5 | 82.7 | 77.8 | 77.7 | 77.5 |
| YCNV YC3 | 99.2 | *** | 99.4 | 83 | 83 | 83 | 83 | 83 | 83.1 | 83.1 | 82.8 | 82.8 | 82.8 | 82.8 | 82.5 | 82.5 | 82.7 | 77.7 | 77.7 | 77.4 |
| YCNV FZ3 | 99.2 | 99.6 | *** | 83.1 | 83 | 83 | 83 | 83.1 | 83.1 | 83.1 | 82.9 | 82.9 | 82.9 | 82.9 | 82.6 | 82.5 | 82.7 | 77.7 | 77.7 | 77.4 |
| BJNV SL4 | 88.4 | 88.5 | 88.4 | *** | 99.7 | 99.8 | 99.7 | 99.5 | 99.7 | 98.6 | 99.1 | 99.1 | 99.1 | 97.7 | 98.2 | 98.1 | 97.4 | 77.7 | 77.7 | 77.4 |
| BJNV SL3 | 88.4 | 88.5 | 88.4 | 99.8 | *** | 99.8 | 99.7 | 99.5 | 99.7 | 98.6 | 99.1 | 99 | 99 | 97.7 | 98.1 | 98.1 | 97.4 | 77.7 | 77.7 | 77.5 |
| BJNV TH3 | 88.4 | 88.5 | 88.4 | 99.9 | 99.8 | *** | 99.8 | 99.5 | 99.7 | 98.7 | 99.1 | 99.1 | 99.1 | 97.8 | 98.2 | 98.1 | 97.3 | 77.6 | 77.7 | 77.4 |
| BJNV TH4 | 88.4 | 88.5 | 88.4 | 99.9 | 99.8 | 99.9 | *** | 99.6 | 99.7 | 98.7 | 99.2 | 99.1 | 99.1 | 97.8 | 98.2 | 98.1 | 97.4 | 77.7 | 77.7 | 77.5 |
| BJNV YC4 | 88.5 | 88.5 | 88.5 | 99.8 | 99.7 | 99.8 | 99.8 | *** | 99.6 | 98.7 | 99.3 | 99.1 | 99.2 | 97.8 | 98.1 | 98.1 | 97.4 | 77.7 | 77.7 | 77.4 |
| BJNV YC3 | 88.5 | 88.5 | 88.4 | 99.9 | 99.8 | 99.9 | 99.8 | 99.8 | *** | 98.8 | 99.2 | 99.2 | 99.2 | 97.9 | 98.2 | 98.2 | 97.5 | 77.7 | 77.7 | 77.5 |
| BJNV DH3 | 88.5 | 88.7 | 88.5 | 98.6 | 98.5 | 98.5 | 98.5 | 98.5 | 98.7 | *** | 98.4 | 98.3 | 98.3 | 98 | 97.6 | 97.5 | 97.5 | 77.6 | 77.6 | 77.4 |
| BJNV H1603 | 88.3 | 88.4 | 88.3 | 99.4 | 99.3 | 99.4 | 99.4 | 99.5 | 99.5 | 98.3 | *** | 99.2 | 99.9 | 97.8 | 98 | 98 | 97 | 77.6 | 77.6 | 77.3 |
| BJNV H160 | 88.2 | 88.3 | 88.2 | 99.3 | 99.3 | 99.3 | 99.3 | 99.3 | 99.3 | 98.2 | 99.4 | *** | 99.1 | 97.7 | 97.9 | 97.9 | 97 | 77.5 | 77.5 | 77.2 |
| BJNV H801 | 88.2 | 88.3 | 88.2 | 99.3 | 99.2 | 99.3 | 99.3 | 99.4 | 99.4 | 98.2 | 99.9 | 99.3 | *** | 97.7 | 97.9 | 97.9 | 97 | 77.5 | 77.6 | 77.3 |
| BJNV H39 | 88.2 | 88.3 | 88.2 | 98.5 | 98.5 | 98.5 | 98.5 | 98.5 | 98.5 | 98.2 | 98.6 | 98.5 | 98.5 | *** | 97.2 | 97.2 | 96.9 | 77.4 | 77.4 | 77.2 |
| BJNV H56 | 87.8 | 87.8 | 87.7 | 98.5 | 98.5 | 98.6 | 98.5 | 98.5 | 98.6 | 97.5 | 98.4 | 98.5 | 98.3 | 97.8 | *** | 99.9 | 96.6 | 77.4 | 77.4 | 77.2 |
| BJNV H59 | 87.7 | 87.8 | 87.7 | 98.4 | 98.4 | 98.4 | 98.4 | 98.4 | 98.5 | 97.4 | 98.4 | 98.3 | 98.3 | 97.8 | 99.9 | *** | 96.5 | 77.4 | 77.4 | 77.1 |
| GKTV | 88.3 | 88.4 | 88.3 | 98.2 | 98.3 | 98.2 | 98.2 | 98.3 | 98.3 | 98 | 97.9 | 97.8 | 97.8 | 97.7 | 97.1 | 97 | *** | 77.6 | 77.6 | 77.4 |
| PTV | 84.8 | 84.9 | 84.8 | 84 | 84.1 | 84 | 84 | 84 | 84.1 | 84.1 | 83.9 | 83.8 | 83.8 | 83.6 | 83.3 | 83.3 | 83.9 | *** | 97.4 | 98.8 |
| NWNV1 | 84.7 | 84.8 | 84.8 | 83.8 | 83.9 | 83.8 | 83.8 | 83.8 | 83.9 | 83.9 | 83.7 | 83.7 | 83.7 | 83.4 | 83.1 | 83.1 | 83.8 | 98.3 | *** | 96.6 |
| GTV | 84.3 | 84.4 | 84.3 | 83.5 | 83.5 | 83.5 | 83.5 | 83.5 | 83.5 | 83.5 | 83.3 | 83.2 | 83.2 | 83 | 82.8 | 82.8 | 83.3 | 98.6 | 97.3 | *** |

^*^ Abbreviations: YCNV, Yichun nariovirus; BJNV, Beiji nariovirus; GKTV, Gakugsa tick virus; PTV, Pustyn virus; NWNV1, Norway nairovirus 1; GTV, Grotenhout virus.
