# Supplementary material for: Extensive diversity of RNA viruses in ticks revealed by metagenomics in northeastern China
Source: PLoS Negl Trop Dis. 2022 Dec 21;16(12):e0011017. doi: 10.1371/journal.pntd.0011017 (PMC9836300; doi:10.1371/journal.pntd.0011017)
Supplement: S11 Table — (DOCX) [file pntd.0011017.s011.docx]

S11 Table. Nucleotide sequence similarity of the S segment (upper right) of YCNV and BJNV^*^

|  | YCNV YC4 | YCNV YC3 | YCNV FZ3 | BJNV SL4 | BJNV SL3 | BJNV TH3 | BJNV TH4 | BJNV YC4 | BJNV YC3 | BJNV DH3 | BJNV YKS44 | BJNV H1603 | BJNV H160 | BJNV H801 | BJNV H39 | BJNV H56 | BJNV H59 | GKTV | PTV | NWNV1 | GTV |
| --- | --- | --- | --- | --- | --- | --- | --- | --- | --- | --- | --- | --- | --- | --- | --- | --- | --- | --- | --- | --- | --- |
| YCNV YC4 | *** | 98.7 | 98.7 | 83.3 | 83 | 83.2 | 83.2 | 83.2 | 83.2 | 83.2 | 83.4 | 83.6 | 83.3 | 83 | 83 | 82.7 | 82.3 | 83.5 | 76.1 | 79.4 | 78.9 |
| YCNV YC3 | *** | *** | 98.7 | 83.5 | 83.3 | 83.5 | 83.5 | 83.5 | 83.5 | 83.4 | 83.6 | 83.9 | 83.5 | 83.2 | 83.2 | 82.9 | 82.6 | 83.8 | 76 | 79.4 | 78.8 |
| YCNV FZ3 | *** | *** | *** | 83.5 | 83.3 | 83.5 | 83.5 | 83.3 | 83.3 | 83.4 | 83.5 | 83.8 | 83.5 | 83.2 | 83.2 | 82.9 | 82.4 | 83.8 | 76.2 | 79.6 | 79 |
| BJNV SL4 | *** | *** | *** | *** | 99.4 | 99.9 | 99.9 | 99.7 | 99.6 | 97.6 | 99.4 | 98.6 | 99 | 99 | 99 | 98.6 | 98.3 | 98.4 | 76.3 | 79.3 | 79.2 |
| BJNV SL3 | *** | *** | *** | *** | *** | 99.5 | 99.5 | 99.3 | 99.5 | 97.4 | 98.9 | 98.4 | 98.9 | 98.9 | 98.9 | 98.1 | 97.8 | 98.1 | 76.3 | 79.3 | 79.2 |
| BJNV TH3 | *** | *** | *** | *** | *** | *** | 100 | 99.8 | 99.7 | 97.7 | 99.5 | 98.6 | 99.1 | 99.1 | 99.1 | 98.6 | 98.4 | 98.3 | 76.3 | 79.3 | 79.2 |
| BJNV TH4 | *** | *** | *** | *** | *** | *** | *** | 99.8 | 99.7 | 97.7 | 99.5 | 98.6 | 99.1 | 99.1 | 99.1 | 98.6 | 98.4 | 98.3 | 76.3 | 79.3 | 79.2 |
| BJNV YC4 | *** | *** | *** | *** | *** | *** | *** | *** | 99.8 | 97.7 | 99.6 | 98.7 | 99 | 99 | 99 | 98.7 | 98.5 | 98.2 | 76.3 | 79.3 | 79.2 |
| BJNV YC3 | *** | *** | *** | *** | *** | *** | *** | *** | *** | 97.7 | 99.4 | 98.8 | 99.2 | 99.2 | 99.2 | 98.6 | 98.3 | 98.4 | 76.3 | 79.3 | 79.3 |
| BJNV DH3 | *** | *** | *** | *** | *** | *** | *** | *** | *** | *** | 97.3 | 97.7 | 97.3 | 97.4 | 97.4 | 96.6 | 96.2 | 96.8 | 76.1 | 79 | 79.1 |
| BJNV YKS44 | *** | *** | *** | *** | *** | *** | *** | *** | *** | *** | *** | 98.4 | 98.7 | 98.6 | 98.6 | 99.2 | 98.9 | 97.9 | 76.2 | 79.2 | 79.1 |
| BJNV H1603 | *** | *** | *** | *** | *** | *** | *** | *** | *** | *** | *** | *** | 98.4 | 98.4 | 98.4 | 97.7 | 97.4 | 98 | 76 | 79 | 79 |
| BJNV H160 | *** | *** | *** | *** | *** | *** | *** | *** | *** | *** | *** | *** | *** | 98.9 | 98.9 | 97.8 | 97.6 | 98.1 | 76.2 | 79.2 | 79.1 |
| BJNV H801 | *** | *** | *** | *** | *** | *** | *** | *** | *** | *** | *** | *** | *** | *** | 99.8 | 97.8 | 97.5 | 98.1 | 76 | 79 | 78.9 |
| BJNV H39 | *** | *** | *** | *** | *** | *** | *** | *** | *** | *** | *** | *** | *** | *** | *** | 97.8 | 97.5 | 98.2 | 76.1 | 79.1 | 79 |
| BJNV H56 | *** | *** | *** | *** | *** | *** | *** | *** | *** | *** | *** | *** | *** | *** | *** | *** | 99.3 | 97.1 | 75.6 | 78.6 | 78.5 |
| BJNV H59 | *** | *** | *** | *** | *** | *** | *** | *** | *** | *** | *** | *** | *** | *** | *** | *** | *** | 96.8 | 75.4 | 78.3 | 78.3 |
| GKTV | *** | *** | *** | *** | *** | *** | *** | *** | *** | *** | *** | *** | *** | *** | *** | *** | *** | *** | 76 | 79 | 79 |
| PTV | *** | *** | *** | *** | *** | *** | *** | *** | *** | *** | *** | *** | *** | *** | *** | *** | *** | *** | *** | 95.1 | 95.9 |
| NWNV1 | *** | *** | *** | *** | *** | *** | *** | *** | *** | *** | *** | *** | *** | *** | *** | *** | *** | *** | *** | *** | 98.6 |
| GTV | *** | *** | *** | *** | *** | *** | *** | *** | *** | *** | *** | *** | *** | *** | *** | *** | *** | *** | *** | *** | *** |

^*^ Abbreviations: YCNV, Yichun nariovirus; BJNV, Beiji nariovirus; GKTV, Gakugsa tick virus; PTV, Pustyn virus; NWNV1, Norway nairovirus 1; GTV, Grotenhout virus.
