# Supplementary material for: Extensive diversity of RNA viruses in ticks revealed by metagenomics in northeastern China
Source: PLoS Negl Trop Dis. 2022 Dec 21;16(12):e0011017. doi: 10.1371/journal.pntd.0011017 (PMC9836300; doi:10.1371/journal.pntd.0011017)
Supplement: S12 Table — (DOCX) [file pntd.0011017.s012.docx]

S12 Table. Nucleotide sequence similarity of the L segment (upper right) and amino acid sequence similarity of the RdRp (lower left) of MKV and MJPV^*^

|  | MKV MKW73 | MKV TH3 | MKV YC4 | MKV FZ2 | MKV FZ3 | MKV ShL2 | MKV DH3 | KYV CZCT80Q | MJPV FZ3 | MJPV MDJ2 |
| --- | --- | --- | --- | --- | --- | --- | --- | --- | --- | --- |
| MKV MKW73 | *** | 93.1 | 92.5 | 92.4 | 92.4 | 92.5 | 92.9 | 83 | 76.2 | 76.1 |
| MKV TH3 | 99.1 | *** | 95.8 | 95.7 | 95.7 | 95.8 | 94.3 | 83.2 | 75.9 | 76 |
| MKV YC4 | 99.1 | 99.2 | *** | 96.2 | 96.2 | 96.3 | 94.3 | 82.7 | 76.1 | 75.9 |
| MKV FZ2 | 99.1 | 99.2 | 99.6 | *** | 100 | 98.3 | 95.4 | 82.5 | 76 | 76 |
| MKV FZ3 | 99.1 | 99.2 | 99.6 | 100 | *** | 98.3 | 95.4 | 82.5 | 76 | 76 |
| MKV ShL2 | 99 | 99.1 | 99.5 | 99.7 | 99.7 | *** | 95.7 | 82.6 | 75.9 | 75.8 |
| MKV DH3 | 99 | 98.9 | 99.1 | 99.3 | 99.3 | 99.4 | *** | 82.8 | 76.1 | 76.2 |
| KYV CZCT80Q | 95.3 | 95.3 | 95.3 | 95.3 | 95.3 | 95.2 | 95.1 | *** | 77.4 | 77.3 |
| MJPV FZ3 | 88.7 | 88.8 | 88.7 | 88.7 | 88.7 | 88.6 | 88.6 | 88.2 | *** | 95.2 |
| MJPV MDJ2 | 88.9 | 89.1 | 88.9 | 88.9 | 88.9 | 88.8 | 88.8 | 88.4 | 99.5 | *** |

^*^ Abbreviations: MKV, Mukawa virus; KYV, Kuriyama virus; MJPV, Mudanjiang phlebovirus.
