# Supplementary material for: Extensive diversity of RNA viruses in ticks revealed by metagenomics in northeastern China
Source: PLoS Negl Trop Dis. 2022 Dec 21;16(12):e0011017. doi: 10.1371/journal.pntd.0011017 (PMC9836300; doi:10.1371/journal.pntd.0011017)
Supplement: S13 Table — (DOCX) [file pntd.0011017.s013.docx]

S13 Table. Nucleotide sequence similarity of the S (upper right) and M segments (lower left) of MKV and MJPV^*^

|  | MKV MKW73 | MKV TH3 | MKV YC4 | MKV FZ2 | MKV FZ3 | MKV ShL2 | MKV DH3 | KYV CZCT80Q | MJPV FZ3 | MJPV MDJ2 |
| --- | --- | --- | --- | --- | --- | --- | --- | --- | --- | --- |
| MKV MKW73 | *** | 93.7 | 93.5 | 93.5 | 93.5 | 93.6 | 94 | 80.1 | 70 | 69.8 |
| MKV TH3 | 90.7 | *** | 95.1 | 95 | 95.1 | 96.6 | 94.9 | 80.7 | 70.8 | 70.6 |
| MKV YC4 | 90.9 | 96.1 | *** | 95.8 | 95.9 | 94.8 | 96.8 | 80.3 | 71.1 | 70.4 |
| MKV FZ2 | 92.3 | 91.5 | 91.8 | *** | 99.9 | 94.6 | 95.1 | 80.3 | 70.6 | 70.2 |
| MKV FZ3 | 92.3 | 91.4 | 91.8 | 100 | *** | 94.7 | 95.2 | 80.3 | 70.7 | 70.3 |
| MKV ShL2 | 92.1 | 91.3 | 91.6 | 96.3 | 96.3 | *** | 95.2 | 80.5 | 70.5 | 70.4 |
| MKV DH3 | 92.2 | 91.6 | 92 | 98.3 | 98.3 | 96.8 | *** | 79.5 | 70.5 | 70.3 |
| KYV CZCT80Q | 83.5 | 84.1 | 84.4 | 84.3 | 84.3 | 83.7 | 84.1 | *** | 70.4 | 70.8 |
| MJPV FZ3 | 67.6 | 67.6 | 67.8 | 68.1 | 68.1 | 67.6 | 68 | 68.7 | *** | 96.2 |
| MJPV MDJ2 | 67.6 | 67.8 | 67.8 | 68.2 | 68.2 | 67.8 | 68.1 | 68.4 | 98.8 | *** |

^*^ Abbreviations: MKV, Mukawa virus; KYV, Kuriyama virus; MJPV, Mudanjiang phlebovirus.
