# Supplementary material for: Extensive diversity of RNA viruses in ticks revealed by metagenomics in northeastern China
Source: PLoS Negl Trop Dis. 2022 Dec 21;16(12):e0011017. doi: 10.1371/journal.pntd.0011017 (PMC9836300; doi:10.1371/journal.pntd.0011017)
Supplement: S14 Table — (DOCX) [file pntd.0011017.s014.docx]

S14 Table. Nucleotide sequence similarity of the L segment (upper right) and amino acid sequence similarity of RdRp (lower left) of STPV and OTPV^*^

|  | BTPV1 | NWPV1 | STPV Russia | STPV SL3 | STPV SL4 | STPV TH4 | STPV TH3 | STPV YC4 | STPV YC3 | OTPV Russia | OTPV TH4 | OTPV TH3 | OTPV SL3 | OTPV SL4 | OTPV YC3 | OTPV YC4 |
| --- | --- | --- | --- | --- | --- | --- | --- | --- | --- | --- | --- | --- | --- | --- | --- | --- |
| BTPV1 | *** | 70.6 | 70.1 | 70.2 | 70.1 | 70.1 | 70.1 | 70.3 | 70.2 | 55.9 | 55.9 | 55.7 | 55.9 | 55.8 | 55.8 | 55.9 |
| NWPV1 | 74.4 | *** | 78 | 77.9 | 77.9 | 78 | 77.9 | 78 | 77.9 | 56.7 | 56.8 | 56.8 | 56.7 | 56.7 | 56.8 | 56.8 |
| STPV Russia | 73.2 | 87 | *** | 98.1 | 98.1 | 98.1 | 98.1 | 98 | 98 | 57 | 56.8 | 56.8 | 56.8 | 56.8 | 56.9 | 56.8 |
| STPV SL3 | 73.2 | 87.3 | 99.4 | *** | 99.8 | 99.9 | 99.8 | 99.4 | 99.4 | 57 | 56.7 | 56.8 | 56.8 | 56.7 | 56.8 | 56.8 |
| STPV SL4 | 73.2 | 87.3 | 99.4 | 100 | *** | 99.9 | 99.9 | 99.4 | 99.4 | 57 | 56.8 | 56.8 | 56.8 | 56.7 | 56.8 | 56.8 |
| STPV TH4 | 73.2 | 87.3 | 99.4 | 100 | 100 | *** | 99.9 | 99.4 | 99.5 | 57 | 56.7 | 56.8 | 56.8 | 56.7 | 56.8 | 56.8 |
| STPV TH3 | 73.2 | 87.3 | 99.4 | 100 | 100 | 100 | *** | 99.4 | 99.4 | 57 | 56.7 | 56.8 | 56.8 | 56.7 | 56.8 | 56.8 |
| STPV YC4 | 73.2 | 87.4 | 99.5 | 99.9 | 99.8 | 99.9 | 99.9 | *** | 99.4 | 57 | 56.8 | 56.8 | 56.8 | 56.7 | 56.8 | 56.8 |
| STPV YC3 | 73.2 | 87.3 | 99.5 | 100 | 99.9 | 100 | 100 | 99.9 | *** | 57.1 | 56.8 | 56.8 | 56.8 | 56.8 | 56.9 | 56.8 |
| OTPV Russia | 50 | 50.2 | 50.6 | 50.9 | 50.9 | 50.9 | 50.9 | 50.8 | 50.8 | *** | 99.1 | 98.6 | 98.9 | 98.8 | 98.7 | 98.9 |
| OTPV TH4 | 50 | 50.3 | 50.7 | 50.9 | 50.9 | 50.9 | 50.9 | 50.9 | 50.9 | 99.8 | *** | 99.1 | 99.7 | 99.6 | 99.5 | 99.4 |
| OTPV TH3 | 50 | 50.2 | 50.6 | 50.9 | 50.9 | 50.9 | 50.9 | 50.8 | 50.8 | 99.8 | 99.9 | *** | 99 | 98.9 | 98.8 | 98.9 |
| OTPV SL3 | 50 | 50.3 | 50.7 | 50.9 | 50.9 | 50.9 | 50.9 | 50.9 | 50.9 | 99.8 | 99.9 | 99.9 | *** | 99.9 | 99.6 | 99.4 |
| OTPV SL4 | 50 | 50.2 | 50.6 | 50.8 | 50.9 | 50.8 | 50.8 | 50.8 | 50.8 | 99.6 | 99.7 | 99.7 | 99.8 | *** | 99.5 | 99.3 |
| OTPV YC3 | 50 | 50.3 | 50.7 | 50.9 | 50.9 | 50.9 | 50.9 | 50.9 | 50.9 | 99.6 | 99.7 | 99.7 | 99.8 | 99.6 | *** | 99.4 |
| OTPV YC4 | 50 | 50.2 | 50.6 | 50.9 | 50.9 | 50.9 | 50.9 | 50.8 | 50.8 | 99.6 | 99.7 | 99.7 | 99.8 | 99.6 | 99.9 | *** |

^*^ Abbreviations: BTPV1, Blacklegged tick phlebovirus-1; NWPV1, Norway phlebovirus 1; STPV, Sara tick phlebovirus; OTPV, Onega tick phlebovirus.
