# Supplementary material for: Extensive diversity of RNA viruses in ticks revealed by metagenomics in northeastern China
Source: PLoS Negl Trop Dis. 2022 Dec 21;16(12):e0011017. doi: 10.1371/journal.pntd.0011017 (PMC9836300; doi:10.1371/journal.pntd.0011017)
Supplement: S15 Table — (DOCX) [file pntd.0011017.s015.docx]

S15 Table. Nucleotide sequence similarity of the S segment (upper right) of STPV and OTPV^*^

|  | BTPV1 | NWPV1 | STPV Russia | STPV SL3 | STPV SL4 | STPV TH4 | STPV TH3 | STPV YC4 | STPV YC3 | OTPV Russia | OTPV TH4 | OTPV TH3 | OTPV SL3 | OTPV SL4 | OTPV YC3 | OTPV YC4 |
| --- | --- | --- | --- | --- | --- | --- | --- | --- | --- | --- | --- | --- | --- | --- | --- | --- |
| BTPV1 | *** | 53.3 | 54.8 | 55.1 | 55 | 55 | 55 | 55 | 55 | 38.9 | 39.1 | 39 | 39 | 39 | 38.9 | 39.2 |
| NWPV1 | *** | *** | 83.8 | 83.6 | 83.5 | 83.5 | 83.5 | 83.5 | 83.5 | 53.6 | 53.8 | 53.7 | 53.6 | 53.8 | 53.6 | 53.4 |
| STPV Russia | *** | *** | *** | 99.2 | 99.1 | 99.1 | 99 | 99 | 99 | 56.4 | 56.8 | 56.9 | 56.7 | 56.8 | 57 | 56.8 |
| STPV SL3 | *** | *** | *** | *** | 99.8 | 99.8 | 99.9 | 99.8 | 99.8 | 56.8 | 57.2 | 57.3 | 57.1 | 57.2 | 57.3 | 57.1 |
| STPV SL4 | *** | *** | *** | *** | *** | 100 | 99.9 | 99.6 | 99.6 | 56.7 | 57.1 | 57.2 | 57 | 57.1 | 57.3 | 57.1 |
| STPV TH4 | *** | *** | *** | *** | *** | *** | 99.9 | 99.6 | 99.6 | 56.7 | 57.1 | 57.2 | 57 | 57.1 | 57.3 | 57.1 |
| STPV TH3 | *** | *** | *** | *** | *** | *** | *** | 99.6 | 99.6 | 56.7 | 57.1 | 57.2 | 57 | 57.1 | 57.3 | 57.1 |
| STPV YC4 | *** | *** | *** | *** | *** | *** | *** | *** | 100 | 56.8 | 57.2 | 57.3 | 57.1 | 57.2 | 57.3 | 57.2 |
| STPV YC3 | *** | *** | *** | *** | *** | *** | *** | *** | *** | 56.8 | 57.2 | 57.3 | 57.1 | 57.2 | 57.3 | 57.2 |
| OTPV Russia | *** | *** | *** | *** | *** | *** | *** | *** | *** | *** | 98.8 | 98.8 | 98.8 | 98.4 | 98.6 | 98.5 |
| OTPV TH4 | *** | *** | *** | *** | *** | *** | *** | *** | *** | *** | *** | 99.5 | 99.9 | 99.3 | 99 | 98.8 |
| OTPV TH3 | *** | *** | *** | *** | *** | *** | *** | *** | *** | *** | *** | *** | 99.5 | 99.4 | 99.5 | 99.3 |
| OTPV SL3 | *** | *** | *** | *** | *** | *** | *** | *** | *** | *** | *** | *** | *** | 99.2 | 99 | 98.8 |
| OTPV SL4 | *** | *** | *** | *** | *** | *** | *** | *** | *** | *** | *** | *** | *** | *** | 99 | 98.8 |
| OTPV YC3 | *** | *** | *** | *** | *** | *** | *** | *** | *** | *** | *** | *** | *** | *** | *** | 99.5 |
| OTPV YC4 | *** | *** | *** | *** | *** | *** | *** | *** | *** | *** | *** | *** | *** | *** | *** | *** |

^*^ Abbreviations: BTPV1, Blacklegged tick phlebovirus-1; NWPV1, Norway phlebovirus 1; STPV, Sara tick phlebovirus; OTPV, Onega tick phlebovirus.
