# Supplementary material for: Extensive diversity of RNA viruses in ticks revealed by metagenomics in northeastern China
Source: PLoS Negl Trop Dis. 2022 Dec 21;16(12):e0011017. doi: 10.1371/journal.pntd.0011017 (PMC9836300; doi:10.1371/journal.pntd.0011017)
Supplement: S16 Table — (DOCX) [file pntd.0011017.s016.docx]

S16 Table. Nucleotide sequence similarity of the complete genome (upper right) and amino acid sequence similarity of RdRp (lower left) of THRV1, THRV2, THRV3^*^

|  | THRV2 TH3 | THRV2 TH4 | THRV3 TH3 | THRV1 TH1 | THRV1 TH2 | THRV1 SL1 | THRV1 SL2 | THRV1 DH1 | THRV1 YC2 | THRV1 ShL3 | THRV1 JA | WVMV1 | MLV | HPTV3 | BLTV2 | TCTV3 | WHTV1 |
| --- | --- | --- | --- | --- | --- | --- | --- | --- | --- | --- | --- | --- | --- | --- | --- | --- | --- |
| THRV2 TH3 | *** | 98.9 | 45.9 | 48.7 | 48.6 | 48.7 | 48.6 | 49.4 | 49.3 | 49.3 | 49.3 | 69 | 35.8 | 45.5 | 45.6 | 44.4 | 44.9 |
| THRV2 TH4 | 99.6 | *** | 46.1 | 48.7 | 48.6 | 48.7 | 48.6 | 49.3 | 49.3 | 49.3 | 49.2 | 69.1 | 35.9 | 45.6 | 45.6 | 44.3 | 44.9 |
| THRV3 TH3 | 47.7 | 47.7 | *** | 44.9 | 44.9 | 44.9 | 44.9 | 44.9 | 44.9 | 44.9 | 44.9 | 45.9 | 32.1 | 43 | 42.4 | 45.9 | 43.8 |
| THRV1 TH1 | 50.1 | 50.1 | 44.4 | *** | 99.3 | 100 | 99.3 | 81.4 | 81.4 | 81.4 | 81.4 | 49 | 30.6 | 45.3 | 49.3 | 50.1 | 50.6 |
| THRV1 TH2 | 50.1 | 50.1 | 44.4 | 100 | *** | 99.3 | 100 | 81.4 | 81.4 | 81.4 | 81.4 | 49 | 30.6 | 45.4 | 49.4 | 50.1 | 50.5 |
| THRV1 SL1 | 50.1 | 50.1 | 44.4 | 100 | 100 | *** | 99.3 | 81.4 | 81.4 | 81.4 | 81.4 | 49 | 30.6 | 45.3 | 49.3 | 50.1 | 50.6 |
| THRV1 SL2 | 50.1 | 50.1 | 44.4 | 100 | 100 | 100 | *** | 81.4 | 81.4 | 81.4 | 81.4 | 49 | 30.6 | 45.4 | 49.4 | 50.1 | 50.5 |
| THRV1 DH1 | 50.6 | 50.6 | 44.4 | 93.7 | 93.7 | 93.7 | 93.7 | *** | 99.7 | 99.4 | 99.5 | 48.8 | 30.5 | 45.3 | 49 | 50 | 50.5 |
| THRV1 YC2 | 50.6 | 50.6 | 44.4 | 93.7 | 93.7 | 93.7 | 93.7 | 100 | *** | 99.1 | 99.2 | 48.8 | 30.6 | 45.3 | 49 | 50 | 50.6 |
| THRV1 ShL3 | 50.6 | 50.6 | 44.4 | 93.7 | 93.7 | 93.7 | 93.7 | 99.9 | 99.9 | *** | 99.3 | 48.8 | 30.5 | 45.3 | 49.1 | 49.9 | 50.6 |
| THRV1 JA | 50.6 | 50.6 | 44.4 | 93.6 | 93.6 | 93.6 | 93.6 | 99.7 | 99.7 | 99.7 | *** | 48.8 | 30.4 | 45.3 | 49 | 50 | 50.5 |
| NWMV1 | 79.8 | 79.7 | 48.1 | 51.2 | 51.2 | 51.2 | 51.2 | 51 | 51 | 51 | 51 | *** | 35.5 | 46 | 45.8 | 44.9 | 45.2 |
| MLV | 50.7 | 50.7 | 44.4 | 70.7 | 70.7 | 70.7 | 70.7 | 70.3 | 70.3 | 70.3 | 70.2 | 50.9 | *** | 30.1 | 29.9 | 29.1 | 29.7 |
| HPTV3 | 46.8 | 46.9 | 43.8 | 43.7 | 43.7 | 43.7 | 43.7 | 43.7 | 43.7 | 43.7 | 43.7 | 47.3 | 44.7 | *** | 44 | 43.6 | 44.2 |
| BLTV2 | 45.9 | 45.9 | 41.4 | 49.9 | 49.9 | 49.9 | 49.9 | 49.9 | 49.9 | 49.9 | 49.9 | 46.1 | 50.7 | 41.7 | *** | 46.2 | 46.5 |
| TCTV3 | 45.7 | 45.7 | 43 | 52.3 | 52.3 | 52.3 | 52.3 | 51.8 | 51.8 | 51.8 | 51.8 | 46.8 | 51.5 | 43 | 49 | *** | 58.3 |
| WHTV1 | 45.2 | 45.3 | 41.2 | 49.5 | 49.5 | 49.5 | 49.5 | 49.5 | 49.5 | 49.5 | 49.5 | 45.4 | 50.4 | 41.9 | 47.1 | 64.3 | *** |

^*^ Abbreviations: THRV1, Tahe rhabdovirus 1; THRV2, Tahe rhabdovirus 2; THRV3, Tahe rhabdovirus 3; OTPV, Onega tick phlebovirus; NWMV1: Norway mononegavirus 1; MLV: Manly virus; HPTV3: Huangpi tick virus 3; BLTV2: Bole tick virus 2; Tacheng tick virus 3: TCTV3; WHTV1: Wuhan tick virus 1.
