# Supplementary material for: Extensive diversity of RNA viruses in ticks revealed by metagenomics in northeastern China
Source: PLoS Negl Trop Dis. 2022 Dec 21;16(12):e0011017. doi: 10.1371/journal.pntd.0011017 (PMC9836300; doi:10.1371/journal.pntd.0011017)
Supplement: S17 Table — (DOCX) [file pntd.0011017.s017.docx]

S17 Table. Nucleotide sequence similarity of the complete genome (upper right) and amino acid sequence similarity of RdRp (lower left) of NUMV^*^

|  | DTMV | SFV | LMV | H109 | H43 | H141 | T43 | H159 | H145 | H160 | NE-MDJ2 | NE-FZ3 | NE-FZ4 | NE-SL3 | NE-SL4 | NE-TH4 | NE-YC4 | NE-YC3 | NE-TH3 | NE-DH3 |
| --- | --- | --- | --- | --- | --- | --- | --- | --- | --- | --- | --- | --- | --- | --- | --- | --- | --- | --- | --- | --- |
| DTMV | *** | 99.9 | 64.8 | 64.9 | 65 | 64.8 | 64.9 | 64.6 | 64.5 | 64.7 | 64.9 | 64.9 | 64.9 | 65 | 65 | 65 | 64.9 | 64.9 | 64.9 | 64.9 |
| SFV | 99.9 | *** | 64.8 | 64.9 | 65.1 | 64.9 | 64.9 | 64.7 | 64.6 | 64.8 | 65 | 64.9 | 64.9 | 65 | 65 | 65 | 65 | 64.9 | 64.9 | 64.9 |
| LMV | 76.5 | 76.4 | *** | 96.8 | 96.6 | 97.1 | 97.5 | 96.7 | 96.7 | 96.6 | 96.6 | 96.6 | 96.6 | 98.2 | 98.3 | 96.9 | 97 | 96.7 | 96.8 | 96.8 |
| H109 | 76.6 | 76.6 | 99.4 | *** | 98.5 | 97.6 | 98.1 | 97.5 | 97.4 | 97.4 | 98.8 | 98.9 | 98.9 | 97.9 | 97.8 | 98.9 | 98.7 | 99 | 99.1 | 98.2 |
| H43 | 76.6 | 76.6 | 99.4 | 99.9 | *** | 97.6 | 97.4 | 97.4 | 97.4 | 97.3 | 98.4 | 98.4 | 98.4 | 97.6 | 97.5 | 98.5 | 98.5 | 98.5 | 98.6 | 98.3 |
| H141 | 75.9 | 75.9 | 99 | 98.7 | 98.7 | *** | 99.1 | 97.5 | 97.6 | 97.6 | 97.5 | 97.6 | 97.6 | 98.5 | 98.3 | 97.8 | 97.8 | 97.7 | 97.8 | 97.5 |
| T43 | 75.9 | 75.9 | 99 | 98.7 | 98.6 | 100 | *** | 97.6 | 97.3 | 97.3 | 97.4 | 97.5 | 97.4 | 98.9 | 98.9 | 97.7 | 97.6 | 97.6 | 97.6 | 97.3 |
| H159 | 76.5 | 76.4 | 99.2 | 99.3 | 99.4 | 98.5 | 98.5 | *** | 99.2 | 99 | 97.3 | 97.3 | 97.3 | 97.7 | 97.7 | 97.5 | 97.8 | 97.5 | 97.5 | 97.3 |
| H145 | 76.4 | 76.3 | 99.3 | 99.3 | 99.3 | 98.6 | 98.6 | 99.8 | *** | 99.1 | 97.2 | 97.2 | 97.2 | 97.7 | 97.6 | 97.5 | 97.8 | 97.4 | 97.4 | 97.2 |
| H160 | 76.4 | 76.3 | 99.4 | 99.4 | 99.3 | 98.7 | 98.6 | 99.8 | 99.8 | *** | 97.2 | 97.2 | 97.2 | 97.7 | 97.5 | 97.5 | 97.8 | 97.5 | 97.4 | 97.3 |
| NE-MDJ2 | 76.6 | 76.6 | 99.3 | 99.8 | 99.8 | 98.6 | 98.5 | 99.2 | 99.1 | 99.2 | *** | 99.5 | 99.5 | 97.5 | 97.4 | 99.5 | 99.3 | 99.6 | 99.7 | 98.6 |
| NE-FZ3 | 76.6 | 76.6 | 99.3 | 99.8 | 99.8 | 98.6 | 98.5 | 99.2 | 99.1 | 99.2 | 99.7 | *** | 100 | 97.6 | 97.5 | 99.5 | 99.3 | 99.7 | 99.7 | 98.7 |
| NE-FZ4 | 76.6 | 76.5 | 99.2 | 99.8 | 99.8 | 98.5 | 98.5 | 99.1 | 99.1 | 99.2 | 99.7 | 99.9 | *** | 97.6 | 97.4 | 99.5 | 99.3 | 99.7 | 99.7 | 98.7 |
| NE-SL3 | 76.5 | 76.4 | 99.7 | 99.4 | 99.4 | 99.2 | 99.1 | 99.2 | 99.3 | 99.3 | 99.3 | 99.3 | 99.2 | *** | 99.7 | 97.9 | 97.9 | 97.7 | 97.8 | 97.7 |
| NE-SL4 | 76.5 | 76.4 | 99.8 | 99.4 | 99.4 | 99.3 | 99.2 | 99.3 | 99.4 | 99.4 | 99.3 | 99.3 | 99.3 | 99.9 | *** | 97.8 | 97.8 | 97.6 | 97.7 | 97.6 |
| NE-TH4 | 76.6 | 76.6 | 99.4 | 99.9 | 99.9 | 98.8 | 98.7 | 99.3 | 99.3 | 99.4 | 99.8 | 99.8 | 99.8 | 99.4 | 99.5 | *** | 99.4 | 99.6 | 99.7 | 98.8 |
| NE-YC4 | 76.6 | 76.6 | 99.3 | 99.8 | 99.8 | 98.6 | 98.6 | 99.5 | 99.4 | 99.5 | 99.7 | 99.7 | 99.6 | 99.3 | 99.4 | 99.8 | *** | 99.4 | 99.5 | 98.9 |
| NE-YC3 | 76.6 | 76.6 | 99.4 | 99.9 | 99.8 | 98.8 | 98.7 | 99.4 | 99.3 | 99.4 | 99.7 | 99.8 | 99.8 | 99.4 | 99.5 | 99.8 | 99.7 | *** | 99.9 | 98.8 |
| NE-TH3 | 76.6 | 76.6 | 99.4 | 99.9 | 99.9 | 98.7 | 98.7 | 99.3 | 99.3 | 99.4 | 99.8 | 99.9 | 99.8 | 99.4 | 99.4 | 99.9 | 99.7 | 100 | *** | 98.9 |
| NE-DH3 | 76.6 | 76.5 | 99.5 | 99.8 | 99.7 | 98.8 | 98.8 | 99.4 | 99.4 | 99.5 | 99.6 | 99.6 | 99.6 | 99.5 | 99.6 | 99.7 | 99.7 | 99.8 | 99.8 | *** |

^*^ Abbreviations: NUMV, Nuomin virus; DTMV, Deer tick mononegavirales; SFV, Suffolk virus; LMV, Lesnoe mivirus. NUMV was omitted from the table and replaced by the strain name.
