# Supplementary material for: Extensive diversity of RNA viruses in ticks revealed by metagenomics in northeastern China
Source: PLoS Negl Trop Dis. 2022 Dec 21;16(12):e0011017. doi: 10.1371/journal.pntd.0011017 (PMC9836300; doi:10.1371/journal.pntd.0011017)
Supplement: S18 Table — (DOCX) [file pntd.0011017.s018.docx]

S18 Table. Nucleotide sequence similarity of RdRp genome (upper right) and amino acid sequence similarity of RdRp (lower left) of JPLV1^*^

|  | JPLV1 YC3 | JPLV1 YC4 | JPLV1 SL3 | JPLV1 DH3 | JPLV1 MDJ2 | JPLV1 FZ4 | JPLV1 FZ3 | JPLV1 QG-4 | JPLV1 QG-1 | JPLV1 QG-2 | NPLV1 NOR/S5/Kilen/2014 | NPLV1 NOR/H3/Kilen/2014 |
| --- | --- | --- | --- | --- | --- | --- | --- | --- | --- | --- | --- | --- |
| JPLV1 YC3 | *** | 99.5 | 99.3 | 99.7 | 99.5 | 99.4 | 99.5 | 99.7 | 99.5 | 99.5 | 91.9 | 91.6 |
| JPLV1 YC4 | 100 | *** | 99.8 | 99.7 | 99.6 | 99.7 | 99.5 | 99.5 | 99.4 | 99.4 | 91.9 | 91.5 |
| JPLV1 SL3 | 99.8 | 99.8 | *** | 99.5 | 99.4 | 99.9 | 99.6 | 99.3 | 99.3 | 99.2 | 91.9 | 91.5 |
| JPLV1 DH3 | 99.8 | 99.8 | 99.5 | *** | 99.6 | 99.4 | 99.5 | 99.5 | 99.4 | 99.4 | 91.8 | 91.3 |
| JPLV1 MDJ2 | 100 | 100 | 99.8 | 99.8 | *** | 99.3 | 99.5 | 99.5 | 99.3 | 99.3 | 91.9 | 91.5 |
| JPLV1 FZ4 | 99.8 | 99.8 | 100 | 99.5 | 99.8 | *** | 99.7 | 99.4 | 99.4 | 99.2 | 91.9 | 91.6 |
| JPLV1 FZ3 | 99.8 | 99.8 | 100 | 99.5 | 99.8 | 100 | *** | 99.5 | 99.4 | 99.4 | 91.9 | 91.6 |
| JPLV1 QG-4 | 100 | 100 | 99.8 | 99.8 | 100 | 99.8 | 99.8 | *** | 99.7 | 99.8 | 91.9 | 91.6 |
| JPLV1 QG-1 | 100 | 100 | 99.8 | 99.8 | 100 | 99.8 | 99.8 | 100 | *** | 99.8 | 91.9 | 91.8 |
| JPLV1 QG-2 | 100 | 100 | 99.8 | 99.8 | 100 | 99.8 | 99.8 | 100 | 100 | *** | 91.9 | 91.8 |
| NPLV1 NOR/S5/Kilen/2014 | 94.3 | 94.3 | 94.3 | 94.1 | 94.3 | 94.3 | 94.3 | 94.3 | 94.3 | 94.3 | *** | 98 |
| NPLV1 NOR/H3/Kilen/2014 | 94.1 | 94.1 | 94.1 | 93.9 | 94.1 | 94.1 | 94.1 | 94.1 | 94.1 | 94.1 | 98.6 | *** |

^*^ Abbreviations: JPLV1, Jilin partiti-like virus 1; NPLV1, Norway partiti-like virus 1.
