# Supplementary material for: Extensive diversity of RNA viruses in ticks revealed by metagenomics in northeastern China
Source: PLoS Negl Trop Dis. 2022 Dec 21;16(12):e0011017. doi: 10.1371/journal.pntd.0011017 (PMC9836300; doi:10.1371/journal.pntd.0011017)
Supplement: S19 Table — (DOCX) [file pntd.0011017.s019.docx]

S19 Table. Nucleotide sequence similarity of the complete CDS (upper right) and amino acid sequence similarity of RdRp (lower left) of FLTV^*^

|  | FTLV YC3 | FTLV FZ4 | FTLV FZ3 | FTLV DH3 | UMV | STTLV |
| --- | --- | --- | --- | --- | --- | --- |
| FTLV YC3 | *** | 92.9 | 92.7 | 89.8 | 73.6 | 37 |
| FTLV FZ4 | 98.8 | *** | 93.1 | 92.9 | 73.6 | 37.2 |
| FTLV FZ3 | 94.9 | 95.8 | *** | 92.9 | 73.5 | 36.9 |
| FTLV DH3 | 95.6 | 96.5 | 97.9 | *** | 73.4 | 36.9 |
| UMV | 81.1 | 81.3 | 80.6 | 80.6 | *** | 37.8 |
| STTLV | 30.3 | 30.5 | 30 | 30 | 33.3 | *** |

^*^ Abbreviations: FTLV, Fangzheng tombus-like virus; UMV, Upmeje virus; STTLV, Soybean thrips tombus-like virus; CDS, coding sequence.
