# Supplementary material for: Extensive diversity of RNA viruses in ticks revealed by metagenomics in northeastern China
Source: PLoS Negl Trop Dis. 2022 Dec 21;16(12):e0011017. doi: 10.1371/journal.pntd.0011017 (PMC9836300; doi:10.1371/journal.pntd.0011017)
Supplement: S20 Table — (DOCX) [file pntd.0011017.s020.docx]

S20 Table. Nucleotide sequence identities of complete cds (upper right) and amino acid sequence identities of RdRp (lower left) of ISAV1 and XTAV1*.

|  | ISAV1 ISE6 | NLLV3 | ISAV1 SL4 | ISAV1 SL3 | ISAV1 TH4 | ISAV1 YC4 | ISAV1 YC3 | ISAV1 DH3 | ADTV1 | XTAV1 14-YG | XTAV1 16-T2 | XTAV1 ShL1 | XTAV1 ShL2 | XTAV1 ShL3 | XTAV1 DH2 |
| --- | --- | --- | --- | --- | --- | --- | --- | --- | --- | --- | --- | --- | --- | --- | --- |
| ISAV1 ISE6 | *** | 82.1 | 81.8 | 81.8 | 82.1 | 81.5 | 81.6 | 81.6 | 62.4 | 61.6 | 61.7 | 61.9 | 61.6 | 61.8 | 62.1 |
| NLLV3 | 88.5 | *** | 85.5 | 85.5 | 85.7 | 85.4 | 85.7 | 85.7 | 61.9 | 62.3 | 62.3 | 62.7 | 62.4 | 62.5 | 62.5 |
| ISAV1 SL4 | 87.7 | 93.4 | *** | 100 | 98.6 | 98.8 | 99.1 | 99.2 | 61.7 | 62.1 | 62 | 62.4 | 61.9 | 62.3 | 62.3 |
| ISAV1 SL3 | 87.7 | 93.4 | 100 | *** | 98.6 | 98.8 | 99.1 | 99.2 | 61.7 | 62.1 | 62 | 62.4 | 61.9 | 62.3 | 62.3 |
| ISAV1 TH4 | 87.7 | 93.4 | 100 | 100 | *** | 98.1 | 98.8 | 98.6 | 62.3 | 62.3 | 62.4 | 62.8 | 62.3 | 62.8 | 62.6 |
| ISAV1 YC4 | 87.7 | 93.4 | 100 | 100 | 100 | *** | 99 | 98.7 | 62 | 62 | 62.1 | 62.6 | 62.1 | 62.4 | 62.4 |
| ISAV1 YC3 | 87.7 | 93.4 | 100 | 100 | 100 | 100 | *** | 99 | 61.9 | 62 | 62.1 | 62.7 | 62.2 | 62.5 | 62.4 |
| ISAV1 DH3 | 87.3 | 93 | 99.6 | 99.6 | 99.6 | 99.6 | 99.6 | *** | 62 | 62.1 | 62 | 62.4 | 61.9 | 62.3 | 62.2 |
| ADTV1 | 70.5 | 70.5 | 72.1 | 72.1 | 72.1 | 72.1 | 72.1 | 71.7 | *** | 66.7 | 66.8 | 67.1 | 67.1 | 66.7 | 67 |
| XTAV1 14-YG | 67.9 | 70 | 69.1 | 69.1 | 69.1 | 69.1 | 69.1 | 68.7 | 78.2 | *** | 99.3 | 96.1 | 96.2 | 95.9 | 96.1 |
| XTAV1 16-T2 | 67.9 | 70 | 69.1 | 69.1 | 69.1 | 69.1 | 69.1 | 68.7 | 78.2 | 100 | *** | 96.2 | 96.2 | 96 | 96 |
| XTAV1 ShL1 | 67.9 | 70 | 69.1 | 69.1 | 69.1 | 69.1 | 69.1 | 68.7 | 78.2 | 99.2 | 99.2 | *** | 97.5 | 97.1 | 98.3 |
| XTAV1 ShL2 | 67.9 | 70 | 69.1 | 69.1 | 69.1 | 69.1 | 69.1 | 68.7 | 78.6 | 98.8 | 98.8 | 99.6 | *** | 98.3 | 97.8 |
| XTAV1 ShL3 | 67.9 | 70 | 69.1 | 69.1 | 69.1 | 69.1 | 69.1 | 68.7 | 77.4 | 97.9 | 97.9 | 98.8 | 98.4 | *** | 97.4 |
| XTAV1 DH2 | 67.9 | 70 | 69.1 | 69.1 | 69.1 | 69.1 | 69.1 | 68.7 | 78.6 | 98.8 | 98.8 | 99.6 | 100 | 98.4 | *** |

* Abbreviations: ISAV1, *Ixodes scapularis* associated virus 1; XTAV1, Xinjiang tick associated virus 1; NLLV3, Norway luteo-like virus 3; ADTV1, American dog tick associated virus1.
