# Supplementary material for: Extensive diversity of RNA viruses in ticks revealed by metagenomics in northeastern China
Source: PLoS Negl Trop Dis. 2022 Dec 21;16(12):e0011017. doi: 10.1371/journal.pntd.0011017 (PMC9836300; doi:10.1371/journal.pntd.0011017)
Supplement: S21 Table — (DOCX) [file pntd.0011017.s021.docx]

S21 Table. Nucleotide sequence identities of complete cds (upper right) and amino acid sequence identities of RdRp (lower left) of JLLV2^*^

|  | JLLV2 SL4 | JLLV2 TH3 | JLLV2 YC4 | JLLV2 YC3 | JLLV2 FZ4 | JLLV2 DH3 | JLLV2 QG-2 | JLLV2 YQG-3 | NLLV2 H3 | NLLV2 A2 |
| --- | --- | --- | --- | --- | --- | --- | --- | --- | --- | --- |
| JLLV2 SL4 | *** | 98.3 | 98.7 | 98.6 | 98.6 | 99 | 98.7 | 98.5 | 87.7 | 87.7 |
| JLLV2 TH3 | 99.5 | *** | 98.3 | 98.2 | 98.1 | 98.6 | 98.4 | 98.3 | 87.9 | 87.9 |
| JLLV2 YC4 | 99.8 | 99.8 | *** | 100 | 98.9 | 99.3 | 98.6 | 98.5 | 87.9 | 87.9 |
| JLLV2 YC3 | 99.8 | 99.8 | 100 | *** | 98.9 | 99.2 | 98.6 | 98.4 | 88 | 88 |
| JLLV2 FZ4 | 99.5 | 99.5 | 99.8 | 99.8 | *** | 99.2 | 98.7 | 98.4 | 87.9 | 87.9 |
| JLLV2 DH3 | 99.9 | 99.6 | 99.9 | 99.9 | 99.6 | *** | 99 | 98.8 | 88.2 | 88.2 |
| JLLV2 QG-2 | 99.6 | 99.6 | 99.9 | 99.9 | 99.6 | 99.8 | *** | 99.8 | 87.9 | 87.9 |
| JLLV2 YQG-3 | 99.5 | 99.8 | 99.8 | 99.8 | 99.5 | 99.6 | 99.9 | *** | 87.9 | 87.9 |
| NLLV2 H3 | 91.7 | 91.5 | 91.5 | 91.5 | 91.3 | 91.7 | 91.4 | 91.5 | *** | 100 |
| NLLV2 A2 | 91.7 | 91.5 | 91.5 | 91.5 | 91.3 | 91.7 | 91.4 | 91.5 | 100 | *** |

* Abbreviations: JLLV2, Jilin luteo-like virus 2; NLLV2, Norway luteo-like virus 2.
