# Supplementary material for: Extensive diversity of RNA viruses in ticks revealed by metagenomics in northeastern China
Source: PLoS Negl Trop Dis. 2022 Dec 21;16(12):e0011017. doi: 10.1371/journal.pntd.0011017 (PMC9836300; doi:10.1371/journal.pntd.0011017)
Supplement: S22 Table — (DOCX) [file pntd.0011017.s022.docx]

S22 Table. Nucleotide sequence identities of complete genome (upper right) of ISAV3*.

|  | ISAV3 TH4 | ISAV3 SL4 | ISAV3 YC4 | ISAV3 YC3 | ISAV3 ISE6 | ISAV4 RTS-11 | XTAV2 15-CYFC39 | XTAV2 381unRc | ADTV2 RTS-100 | ADTV2 RTS-1100 |
| --- | --- | --- | --- | --- | --- | --- | --- | --- | --- | --- |
| ISAV3 SL3 | 94.5 | 99.8 | 94.8 | 93.7 | 87.5 | 88 | 66.4 | 65.3 | 67.9 | 67.9 |
| ISAV3 TH4 | *** | 94.6 | 99.3 | 98.9 | 87.1 | 87.3 | 66.2 | 65.3 | 67.5 | 67.5 |
| ISAV3 SL4 | *** | *** | 95 | 93.9 | 87.6 | 88.2 | 66.4 | 65.5 | 68.1 | 68.1 |
| ISAV3 YC4 | *** | *** | *** | 98.9 | 87.5 | 87.5 | 66.6 | 65.7 | 67.7 | 67.7 |
| ISAV3 YC3 | *** | *** | *** | *** | 86.7 | 86.7 | 66.4 | 66.1 | 67.9 | 67.9 |
| ISAV3 ISE6 | *** | *** | *** | *** | *** | 98.3 | 66.4 | 67.3 | 69.4 | 69.4 |
| ISAV4 RTS-11 | *** | *** | *** | *** | *** | *** | 67 | 67.3 | 69.7 | 69.7 |
| XTAV2 15-CYFC39 | *** | *** | *** | *** | *** | *** | *** | 81.9 | 65.5 | 65.5 |
| XTAV2 381unRc | *** | *** | *** | *** | *** | *** | *** | *** | 66.6 | 66.6 |
| ADTV2 RTS-100 | *** | *** | *** | *** | *** | *** | *** | *** | *** | 100 |

* Abbreviations: ISAV3, *Ixodes scapularis* associated virus 3; ISAV4, *Ixodes scapularis* associated virus 4; XTAV2, Xinjiang tick associated virus 2; ADTV2, American dog tick associated virus 2.
